# Computational and immunoinformatics approaches for designing phytocompound-based drugs and a multi-epitope vaccine targeting FemA, a cell wall protein of *Staphylococcus aureus*

**DOI:** 10.1371/journal.pone.0346271

**Published:** 2026-04-07

**Authors:** Md. Nazmussakib Shuvo, Tawsif Al Arian, Farhan Fuad, Mahmood Hasan Noman, Nuhu Alam, Mahbubul Kabir Himel, Aparna Shil

**Affiliations:** 1 Department of Botany, Jahangirnagar University, Dhaka, Bangladesh; 2 Department of Pharmacy, Jahangirnagar University, Dhaka, Bangladesh; 3 Microbiology program, Mathematics and Natural Science Department, BRAC University, Dhaka, Bangladesh; 4 Department of Statistics, Shahjalal University of Science and Technology, Sylhet, Bangladesh; Albert Einstein College of Medicine, UNITED STATES OF AMERICA

## Abstract

*Staphylococcus aureus*, a bacterial pathogen, is increasingly linked to severe healthcare-associated diseases, from mild skin infections to toxic shock syndrome. Due to limitations in conventional methods, we use computational techniques to screen potential phytocompounds for new drug development and to construct a multiepitope vaccine. Aminoacyltransferase FemA, essential for peptidoglycan formation, was chosen as the target protein for drug and vaccine development. Meanwhile, 1100 phytocompounds were retrieved from 54 plants using the NPASS database and analyzed for drug-likeness and ADMET properties. Paulownin was selected for its higher binding affinity (−7.78 Kcal/mol) than the control drug, Doxycycline (−7.5 Kcal/mol) in molecular docking. It also showed lower RMSD, RMSF, and stronger hydrogen bonding (1.11/0.8 Å, 1.594/1.613 Å, and 3/2, respectively) in molecular dynamics simulations. It demonstrated potential higher affinity, scoring −39.74 ± 6.05 Kcal/mol, outperforming Doxycycline's −28.15 ± 5.90 Kcal/mol in MM-GBSA. For the vaccine, 12 selected epitopes were compiled utilizing GPGPG, two KK peptides, and AAY linkers. The N-flanking immunogenicity of the vaccine was enhanced by adding a lipoprotein adjuvant, LprG. The vaccine alone and the vaccine with the receptor molecule TLR2 showed RMSF (4.247 Å/2.42 Å), RMSD (12.9 Å/7.52 Å), SASA (228.48 nm²/220.29 nm²), Rg (33.76 Å/28.48 Å), and hydrogen bonds (171/165), indicating the vaccine’s predicted immune response patterns. These findings computationally prioritize Paulownin and the multi-epitope construct as candidates for further experimental evaluation. As the study is entirely in silico, experimental validation is required to confirm biological activity and immunogenicity.

## 1. Introduction

In 1880, *Staphylococcus aureus* was first observed by the Scottish surgeon Alexander Ogston from a patient with ulcerated sores [1]. It colonizes the perineum, the principal site of the skin, and can transform into an opportunistic bacterium that can cause normal skin infections to life-threatening conditions [2]. Skin infections like carbuncle, cellulitis, impetigo bullosa wound infection, or furuncle are the initial manifestations of staphylococcal infection, then progress to systemic dissemination such as osteomyelitis, endocarditis, brain abscesses, pneumonia, meningitis, and bacteremia [3,4]. According to studies, mortality rates from *S. aureus* bacteremia (SAB) range from 10 to 30 percent [5]. The pathogenic activity of *S. aureus* is mainly due to its ability to produce a wide variety of virulence factors. Nevertheless, other virulence factors, such as peptidoglycan, teichoic acids, capsules, enzymes, and various extracellular toxins, are responsible for pathogenicity [6].

*Staphylococcus* species are among the leading causes of both community- and hospital-acquired infections, and the rapid emergence of antimicrobial resistance has significantly reduced the effectiveness of existing therapeutic options. Immunoinformatics-driven reverse vaccinology enables systematic screening of large protein datasets, including virulence factors and essential gene products, to identify antigenic targets capable of stimulating multiple arms of the immune system. Previous large-scale studies have demonstrated that integrating predictions of T-cell epitopes, B-cell epitopes, MHC binders, cytokine-inducing regions, and self-tolerance filtering against the human proteome significantly enhances the biological relevance and safety of candidate vaccines [7]. Such strategies have been successfully applied across multiple pathogenic bacteria, highlighting proteins that are both essential for bacterial survival and virulence as particularly promising vaccine targets. However, most previous studies have focused primarily on antigen prediction and have not explored whether the same targets could be exploited simultaneously for therapeutic drug development.

The aminoacyltransferase FemA enzyme plays a vital role in peptidoglycan biosynthesis in the cell wall of *Staphylococcus aureus*. It catalyzes the formation of the pentaglycine interpeptide bridge, therefore, blocking it weakens the cell wall and makes it harder for bacteria to survive [8,9]. It combines glycine 2 and 3, utilizing glycyl-tRNA (Gly) as a donor. Furthermore, it is recognized for its pivotal role in the manifestation of methicillin resistance in the organism [9,10]. The alarming rise of resistance to antibiotics is a growing public health crisis that is responsible for the deaths of millions of individuals. According to reports, the first decade of the 21st century witnessed a notable decline in the effectiveness of antibiotics and the progressive emergence of multidrug-resistance (MDR) in combating bacteria. Moreover, these bacteria were designated by the World Health Organization (WHO) in 2017 as superbugs of significant concern to human health due to their invasive nature, resistance to antibiotics, and toxin-mediated pathogenesis [11,12]. Since the FemA is responsible for the process of linking peptide strands together, which is crucial for the stability of the cell wall and is accountable for the expression of methicillin resistance, it might be a promising target for developing novel drugs against the bacteria. While the FemA has been investigated in the context of bacterial physiology and resistance mechanisms, its potential as a dual-purpose target for both immunization and small-molecule inhibition remains underexplored [8].

Recent advances in immunoinformatics have substantially improved epitope-based vaccine design by incorporating immune-response modeling and cytokine prediction. Studies by Usmani et al. (2018), Nagpal et al. (2017), and Dhall et al. (2023; 2021) have demonstrated that integrating cytokine-induction potential—such as IFN-γ, IL-4, IL-6, and IL-10 responses—enhances the biological relevance and predictive accuracy of computational vaccine constructs [13–16]. Similarly, modern machine learning–based frameworks have enabled more robust evaluation of host immune activation profiles, strengthening confidence in in silico vaccine candidates [17]. Parallel to vaccine development, plant-derived phytochemicals have gained attention as a rich source of antimicrobial agents due to their structural diversity, biological activity, and generally favorable safety profiles. Consequently, phytocompounds have emerged as highly promising candidates for treating multidrug-resistant bacterial infections and are considered among the most effective alternatives. These compounds bind to the cell wall-synthesizing protein, forming a complex that disrupts or inactivates the organism's cell wall. Additionally, 80% of human illnesses, including immunological disorders, cancer, and bacterial infection, are managed using plant-originated natural products and their derivatives, which also account for one-fourth of all prescribed drugs worldwide [18,19]. Either these drugs can be produced through the conventional drug design approach, which necessitates millions of dollars and an effort of ten to fifteen years and a success rate of only 13%, or using computer-aided drug discovery (CADD) tools that hasten the discovery of drugs and the process of development, thus lowering expenses and failures in the last phase [20,21]. While docking and molecular dynamics simulations cannot substitute for biochemical validation, layered computational filtering—combining docking, trajectory stability, and binding free-energy rescoring—can reduce false-positive prioritization in early-stage discovery. Nevertheless, systematic and large-scale integration of phytochemical screening with reverse vaccinology targets is still limited.

Furthermore, a multi-epitope vaccine may be a viable solution for combating *S. aureus.* However, it is challenging to develop an effective vaccine against the bacterium due to its ability to produce a wide array of virulence factors. Epitopes that elicit humoral and cellular immune responses may be utilized to circumvent the problem of human vaccine failure [22]. Prolonged advancements in the domain of immunoinformatics over the last decade, in conjunction with computational tools, have improved and expedited the development of vaccines against pathogens that have previously proven difficult to target or only partially successful, such as *Listeria monocytogenes* and *Mycobacterium tuberculosis* [23,24]. Compared to conventional vaccine design, immunoinformatic methods are more accurate, economical, dependable, and time-efficient. By employing contemporary techniques, in silico assessments of human antigen-based allergenicity and toxicity can provide comprehensive insights while also simplifying the resolution of challenges associated with these criteria [25]. Previous research has validated the safety and effectiveness of bacterial immunizations targeting specific toxins or antigens, particularly capsular polysaccharides. Tetanus toxoid and pneumococcal conjugate vaccines are well-known examples [26]. According to those assessments, the FemA protein has the potential to serve as a target for the development of a multi-epitope vaccine against *S. aureus.*

Although several reverse vaccinology–based vaccine design studies against *Staphylococcus aureus* have been reported that targeted surface adhesins, toxins, or capsular polysaccharides and failed in clinical trials (e.g., due to insufficient functional immunity, strain variability escape, or immune skewing) [27–31], most focus on pan-genomic antigen screening or surface-exposed proteins without integrating cytokine-level immune modulation or receptor-level dynamic validation. In contrast, the present study specifically targets the FemA, an essential enzyme involved in peptidoglycan biosynthesis and methicillin resistance, and applies an expanded immunoinformatics framework incorporating IFN-γ, IL-4, and IL-10 cytokine prediction, Toll-like receptor docking, molecular dynamics simulation, and immune response modeling.

MRSA control requires both improved therapeutics and durable prevention. In the context of the urgent need for both improved antimicrobial agents and more effective preventive strategies against *S. aureus*, the present study investigates the FemA protein as a biologically validated and mechanistically central target within the bacterial cell wall biosynthesis pathway. Because FemA is essential for pentaglycine bridge formation and contributes to methicillin resistance expression, it represents a functionally constrained enzymatic node whose disruption may compromise bacterial survival. At the same time, sequence-derived regions of FemA may contain immunogenic determinants capable of stimulating adaptive immune responses when appropriately engineered within a multi-epitope framework. Rather than implying direct clinical synergy, we approach FemA as a single experimentally characterized target that enables parallel computational exploration of therapeutic inhibition and immunoinformatic vaccine design within a unified biological context.

Accordingly, this study was designed to evaluate whether FemA possesses structural features compatible with stable phytocompound binding and sequence regions predicted to elicit balanced cellular and humoral immune responses under rigorous in silico screening conditions. Structure-based virtual screening, MD simulations, and MM-GBSA rescoring were employed to prioritize phytocompounds with predicted stable engagement of the FemA active region, while reverse vaccinology and immunoinformatics tools were used to identify, filter, and assemble CTL, HTL, and B-cell epitopes into a computationally evaluated multi-epitope construct. By integrating these analyses within a single framework, the work aims to generate high-confidence computational candidates for downstream experimental validation, while acknowledging that predictive modeling does not substitute for biological confirmation.

## 2. Materials and methods

**Fig 1:**
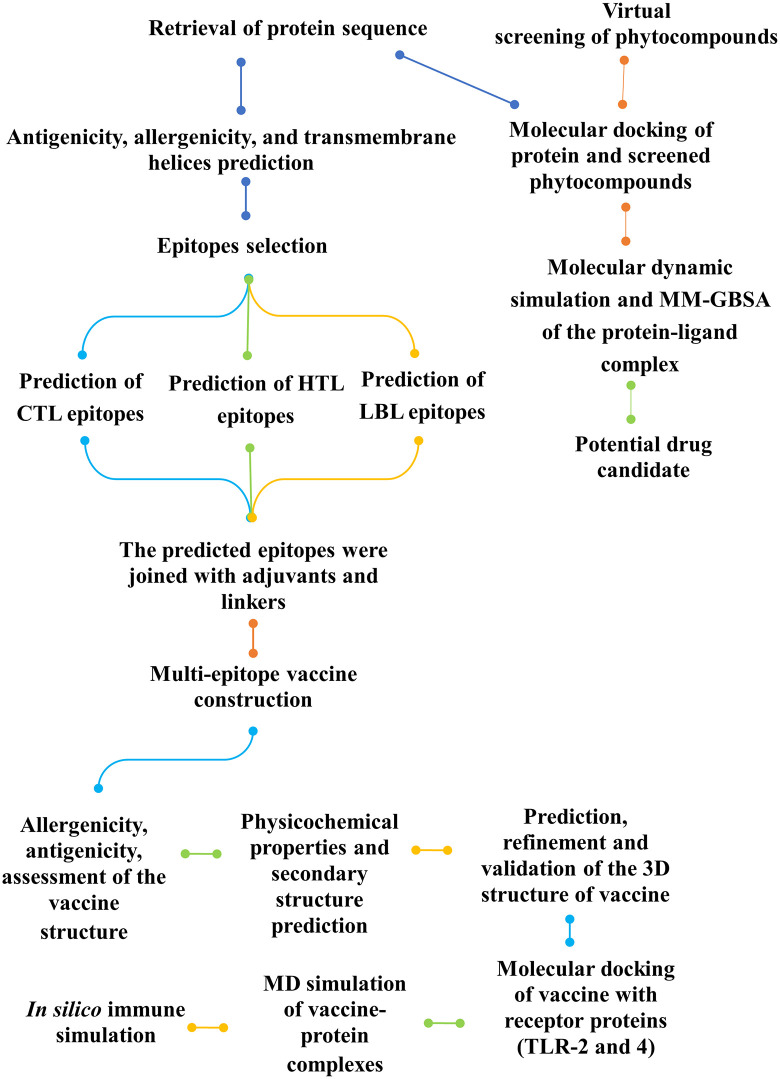
The comprehensive workflow of our research. The computational approach was used to develop phytocompound-based drugs and construct a multi-epitope vaccine.

A brief illustration of the methodology of our research work is depicted in Fig 1.

### 2.1 Drug design

#### 2.1.1 Selection of the target protein.

The aminoacyltransferase FemA of *Staphylococcus aureus* was selected as the target protein (Uniprot ID: P0A0A5) from the databank of proteins (PDB) (https://www.rcsb.org/) based on its functional role in the survival of the pathogen [9,32]. After retrieving the amino acid sequence from UniProt (https://www.uniprot.org/), the 3D structure was predicted through homology modeling using 1lrz template (identity: 100 percent and GMQE value: 0.98) with the SWISS-MODEL server (https://swissmodel.expasy.org/) [33,34]. Along with that, the model predicted by the AlphaFold server (https://alphafoldserver.com/) was downloaded from the UniProt database and compared with the models generated by SWISS-MODEL [35]. GalaxyWeb (https://galaxy.seoklab.org/cgi-bin/submit.cgi?type=REFINE) was employed to refine and validate the model using RMSD values, MolProbity scores, and clash scores [36]. For additional validation, the flexibility and stability of the model were assessed using molecular dynamics simulation [37]. Furthermore, the active site of the FemA protein was predicted using the Computed Atlas of Surface Topography of Proteins (CASTp 3.0; (http://sts.bioe.uic.edu/castp/index.html?2cpk)), which identifies surface pockets and cavities based on geometric and topological analyses of protein structures. CASTp calculates pocket area and volume using solvent-accessible surface mapping, enabling the identification of potential ligand-binding regions. The predicted binding pockets were further examined using BIOVIA Discovery Studio to visualize cavity architecture, residue composition, and spatial accessibility, thereby facilitating selection of an appropriate docking site [38,39].

#### 2.1.2 Phytocompound selection.

Phytocompounds of indigenous plants of Bangladesh were obtained from the NPASS (https://bidd.group/NPASS/) database, and the structures and Canonical SMILES of the phytocompounds were collected from the PubChem (https://pubchem.ncbi.nlm.nih.gov/) database [40,41]. Drug-like and ADMET properties were determined through several servers, including SwissADME (http://www.swissadme.ch/) and pkCSM (https://biosig.lab.uq.edu.au/pkcsm/), to ensure the non-toxicity of the phytocompounds [42]. SwissADME applies five Lipinski's rules, Muegge's rules, Ghose's rules, Veber's rules, and Egan’s rules to evaluate the compounds [32,43–46]. Posterior to the screening, the phytocompounds were prepared for the molecular docking using the Molecular Graphics Laboratory (MGL) tool [47]. pkCSM considers several parameters, including blood-brain barrier, human intestinal absorption, bioavailability, Caco-2 permeability, CYP1A2 inhibitor, CYP2D6 substrate, excretion, CYP3A4 inhibitor, CYP2C19 inhibitor, P-gp I and II inhibitor, and drug-drug interactions. To investigate toxicity, including AMES toxicity, hERG inhibitors, hepatotoxicity, and maximum acceptable dosage, several *in silico* techniques were employed [48].

#### 2.1.3 Molecular docking.

Molecular docking of the phytocompounds with the FemA protein is expected to be the phytocompound’s preferred mode of binding with the protein [49]. For protein–protein interaction analysis, docking was performed using a data-driven approach in which no predefined grid box was imposed; instead, the interacting interfaces were predicted automatically based on surface complementarity and physicochemical properties of the binding partners. The docking search space encompassed the entire protein surface, allowing unbiased identification of potential interaction regions. Autodock Vina was applied for the molecular docking [50]. The protein and ligands were prepared using the MGL tool, with water molecules and ions removed or added as needed, and saved in pdbqt format [51]. The docked conformations with the most favorable (lowest) binding free energy scores were selected, and the stability of the resulting protein–ligand and protein–protein complexes was subsequently assessed through molecular dynamics simulations to make sure the robustness of the interaction under dynamic conditions [46]. Based on the predicted binding site information, the grid box for the protein was centered at fixed Cartesian coordinates of X = 26.964  Å, Y = 52.754  Å, and Z = 96.692  Å. The grid box dimensions were set to 126  ×  126  × 126 Å along the X, Y, and Z axes, respectively, with a grid spacing of 0.5 Å between adjacent grid points. To select a control drug, three effective antibiotics (Doxycycline, Linezolid, and Trimethoprim) against the organism were docked with the protein [52–54]. The screening was performed three times, and the average value was taken. Following that, the SwissDock server (http://www.swissdock.ch/) was used for further evaluation to select compounds using blind docking that showed at least as good binding as the control. The postures with the highest negative affinity scores will be selected, and their stability will be determined by molecular dynamics simulation [55].

#### 2.1.4 Molecular dynamics (MD) simulation and post MM-GBSA.

To assess the folding properties of the protein, as well as the impact of motion on the binding complex, the compactness of the structure and the fluctuations of the residues, MD simulation was utilized [56]. The MD simulations were designed to evaluate comparative complex stability and interaction persistence rather than to capture full catalytic conformational transitions of FemA, which would require longer-timescale enhanced sampling approaches. The 100 ns MD simulation was performed with Desmond version 6.3 on the Linux operating system using the optimized potentials for liquid simulations (OPLS) force field. It focuses on bond enhancement, such as angle bending, bond stretching, and electrostatic interaction, which are known as non-bonded interactions [57]. Moreover, it was utilized to introduce hydrogen, protonate, and refine through energy constraints [58]. The intricate structures of protein-ligand complexes were solved by utilizing the system designer tool in the cubic TIP3P simulation, a three-point water model. The distance between the solvated region and protein-ligand complexes was a minimum of 10 Å. Following this, the model was normalized by adding sodium and chloride ions to a physiological salt concentration of 0.15 M. Isothermal-isobaric composition (NPT) was utilized in order to carry out the MDS at a temperature of 310 K and a pressure of 1.013 bar. The trajectory was simulated for 100 ns and captured for 100 ps, resulting in 1000 frames saved in memory. Finally, a simulation interaction diagram (SID) was used to analyze the trajectory. The stability of the complex will be assessed using root mean square deviation (RMSD) and root mean square fluctuation (RMSF) [59]. More stable phytocompounds will be considered as drugs and will be tested *in vitro* for further evaluation. Following the completion of the simulations, the MM-GBSA was assessed by MM-GBSA.py. Throughout the evaluation, a periodic frame ranging from 0 to 1000 was incorporated for the study [39].

### 2.2 Vaccine development

#### 2.2.1 Protein selection and Protein curation.

The amino acid sequence of the *Staphylococcus aureus* protein in FASTA format was retrieved from the National Center for Biotechnology Information (NCBI). The structural protein FemA has the Genbank ID: AIU84902.1.

#### 2.2.2 Prediction of the target protein's physicochemical and secondary structure characteristics.

The objective of developing the vaccine model is to stimulate the immune response in a recipient. As a result, the proposed vaccine must possess the following qualities: long-lasting properties, strong immunogenicity, lack of allergenic potential, safety, and improved solubility [60]. Using the ExPASy ProtParam Online server, the biophysical properties of the target proteins were investigated [61]. The current software application calculates physicochemical properties for a specific protein identified in Swiss-Prot or TrEMBL, as well as for a user-defined pattern [62]. The prediction incorporates theoretical variables including atomic mass and isoelectric point, amino acid and atom compositions, and parameters such as half-life, GRAVY (Grand average of hydropathicity), AI (Aliphatic Index), EC (Extinction Coefficients), and II (Index of instability).

#### 2.2.3 Prediction of the selected epitopes.

**Prediction of the B-cell epitopes:** B-cell epitopes are essential constituents of the humoral immune response, wherein the production of immunoglobulin (IgG) is the principal consequence. ABCPred predicted the Linear B Lymphocytes (LBL) epitopes of the FemA protein. ABCPred is accessible at http://crdd.osdd.net/raghava/abcpred/. It generates prognostications by integrating sophisticated artificial neural network techniques with predefined patterns. The default threshold for the 15mer epitope was set to 0.51, and the overlapping filter was initiated automatically. Based on B-cell epitopes, the immunogenicity of antigenic determinants was predicted utilizing the VaxiJen 2.0 webserver (http://www.ddg-pharmfac.net/vaxijen/VaxiJen/VaxiJen.html) [63]. The application of ACC (Auto Cross-Covariance) has enabled the development of a technique that capitalizes on knowledge of the translation of protein domains into uniform carriers that retain fundamental protein properties. The development of predictive models to assess the antigenicity of complete proteins involved the use of databases containing proteins from bacteria, viruses, and tumors. The target was identified as a bacterium, and a predetermined threshold of 0.5 was considered.

Similarly, the allergenic potential was predicted using the AllerTOP 2.0 server (https://www.ddg-pharmfac.net/AllerTOP/) [64]. The ToxinPred webserver (http://crdd.osdd.net/raghava/toxinpred/) was utilized for the purpose of toxicity prediction. The individual epitopes were inserted sequentially. The SVM methodology has been selected as the official predictive technique, and a ten-point E-value threshold has been set. For presentation purposes, the parameters hydrophobicity, hydrophilicity, charge, and molecular weight were selected [65]. The NCBI BLASTp tool, available at https://blast.ncbi.nlm.nih.gov, is a resource employed to distinguish homologous from non-homologous sequences. The calculation determines the statistical significance of the comparison between the provided nucleotide or protein sequences and the conserved sequences. The current inquiry entailed a comparative examination and characterization of the LBL sequence found in non-human organisms, in contrast to the corresponding sequences identified in humans.

**Cytotoxic T lymphocytes (CTL) epitope prediction:** The initial stage of the immune response to viral infections is the recognition of MHC-I antigens by CTL. The immunostimulatory CTL epitope, capable of inducing immunological memory, was identified using the NetCTL 1.2 webserver(http://www.cbs.dtu.dk/services/) [66]. Predictors of CTL antigen composition were generated by the server. In the investigation, threshold values of 0.05, 0.15, and 0.75 were used, respectively, for the antigen processing and transport system, C-terminal dissociation, and epitope identification. In addition to its ability to stimulate the immune system and elicit antibodies, the vaccine epitopes must be free of allergenic or toxic substances. An evaluation was conducted on the immunogenic, antigenic, toxic, and allergic characteristics of every CTL epitope. A hypothesis was formulated suggesting that epitopes with a standard score less than two would manifest effective binding to the HLA allele that corresponds to them. The immunogenicity and antigenicity assessments were conducted using the VaxiJen servers and the IEDB MHCI instrument, respectively. These tools were obtained from the following location: http://tools.iedb.org/immunogenicity [25]. The ToxinPred and AllerTOP v2.0 servers were utilized to evaluate the allergenic and toxicity characteristics. ToxinPred employs machine learning, whereas AllerTOP achieves 94% accuracy in predictions using a descriptor-based biometric technique that does not require any specific configuration [64,67]. The NCBI BLASTp tool was utilized to compare non-homologous and homologous sequences.

**Helper T-cell (HTL) epitope prediction:** Successful eradication of infections and elicitation of immune responses depend on the presence of functional helper T cells (HTCs) [68]. A methodology has been developed by the IEDB server to calculate 15-mer-long HTL epitopes for the most prevalent MHC Class II human genotypes. The appropriate URL for accessing this instrument is http://tools.iedb.org/mhcii/. In the beginning, four-protein sequences were submitted in FASTA format. The NN-align 2.3 (NetMHCII 2.3) method was selected for prediction [69,70]. The locus and species were assigned the designation Human HLA-DR. It was determined that the sequence comprised 15 nucleotides. Peptide classification was performed according to their adjacent ranks. By employing the SWISSPROT database, a percentile score is calculated for each of the five million 15-mer peptides selected at random. The percentage-ranking score assigned to each peptide length was modified in accordance with the frequency of peptide length. A significant correlation is indicated by a low percentile rank. Peptides with percentile ranks below two were selected. It is anticipated that peptides with a high binding capacity will manifest an inhibitory concentration (IC50) of 50 nanomolar. A numerical value below 500 nM signifies a reduced degree of binding affinity, whereas a value surpassing 5000 nM indicates a binding affinity of a moderate magnitude. The antigenicity of the epitope was predicted with the aid of the VaxiJen server [63]. To assess allergenicity and toxicity, the ToxinPred and AllerTOP v2.0 servers were implemented [64,67]. The ACC (Auto Cross-Covariance) method, which entails the translation of protein domains into uniform carriers of essential protein properties, was utilized in the development of AllerTOP v2.0. The ACC methodology is utilized to extract protein sequence. An analysis of QSAR sequences of varying lengths was conducted utilizing this methodology [64]. N-homologous and homologous sequences were distinguished by employing the NCBI Protein BLAST instrument. Utilizing the BLASTp server, similarities between biological sequences are identified.

**IFN-inducing epitope prediction:** It has been observed that the immunomodulatory cytokine interferon-gamma (IFN-γ) stimulates phagocytosis, natural killer (NK) cells, and neutrophils and is considered indispensable for the function of both innate and adaptive immune cells. IFN-gamma activation is an intrinsic quality of the human immune system [71]. Hence, the discovery of epitopes capable of stimulating IFN-γ could potentially augment the immunogenicity of any given vaccination. The prediction of the ability of IFN gamma, IL4, and IL10 to induce HTL epitopes was achieved by employing the IL4pred and IL10pred servers [15,72]. An assessment of the potential of the highest-ranked epitope to induce Th1 immunity via MHC class 2 epitopes, resulting in the production of IFN-γ, was conducted using the IFN epitope server [73,74]. Methods based on motif analysis and support vector machines (SVM) were selected to forecast IFN gamma epitopes. Consequently, the IFN epitope server was generated. It has been observed that IFN-gamma epitopes are present on each structural protein [75]. The service mentioned above provides a unique environment for generating and forecasting protein sequences induced by IFN-γ by utilizing sequence data. The researchers chose to employ a hybrid Support Vector Machine (SVM) and motif-based methods to determine whether the epitope can induce the production of Interferon-gamma (IFN-γ).

#### 2.2.4 Predicted epitope characterization.

**Epitope conservancy analysis:** Discontinuous and linear epitope sequence conservation analyses are two classifications that are discernible. The identified epitopes were analyzed using the epitope conservancy analysis instrument. The instrument was employed to ascertain the correlation between the sequence identities of one *Staphylococcus aureus* target protein, with a threshold of 80%. Each predicted epitope was used as an input against FemA, the target protein of *Staphylococcus aureus,* through the IEDB resource [75,76].

**Populace scope and autoimmunity identification:** HLA genotype frequencies are utilized by the platform to predict population coverage in the immune epitope database and approximate the immune response to a particular set of CTL and HTL overlapping epitopes. A value of 10 was ascribed to a number of epitopes. The user selected the “world” category from the available options in the “Select area(s) and population(s)” column. The merged inventory of Classes I and II was selected from the column labeled “Select calculation options.” In order to assess the capacity of epitopes to elicit autoimmunity, a BLASTp analysis was performed against the human proteome. Epitopes that demonstrated homology with any protein in the human organism were removed [77].

#### 2.2.5 Vaccine construct and analysis.

***Staphylococcus aureus* vaccine production:** For a vaccine to be deemed promising, its sequence must possess the ability to stimulate immune responses from both HTLs and CTLs [78]. Therefore, the vaccine formulation must incorporate suitable CTL and HTL epitopes that are covalently linked. The research utilized CTL and HTL screening methodologies in order to identify potential multiepitope vaccines that possess immune-stimulating characteristics, consistent antigenicity, and lack allergenicity. The present investigation employed GPGPG to establish the linkage between IFN gamma and HTL epitopes, while AAY was utilized to bind the CTL peptides. The N-flank immunogenicity of the vaccine was enhanced by the addition of a Lipoprotein LprG adjuvant. Finally, the model of the vaccine was constructed by the AlphaFold server [79].

**Vaccine physicochemical aspects, antigenicity, and allergenicity:** The evaluation of the modelled vaccine's antigenic properties was performed utilizing the VaxiJen server. Furthermore, the allergenic properties of the epitopes were predicted utilizing the AllerTOP webserver [63,64]. Using NCBI-Protein BLAST, the homology between the *Staphylococcus aureus* vaccine and the human proteome was verified. The physicochemical properties of a constructed vaccine were assessed through the ExPASy ProtParam web server. The evaluation incorporated several parameters of the vaccine, namely its GRAVY (grand average of hydropathicity), AI (Aliphatic Index), II (Instability Index), and EC (Extinction Coefficients), in addition to its half-life.

**Secondary structural analysis:** The current investigation utilized the SOPMA (https://npsa.lyon.inserm.fr/cgi-bin/npsa_automat.pl?page=/NPSA/npsa_sopma.html) and PSIPRED (http://bioinf.cs.ucl.ac.uk/psipred/) methodologies to assess the influence of the developed vaccine's secondary structure on its capacity for protein folding [80,81]. The SOPMA method, which depends on the protein's primary sequence, can be utilized to determine the secondary structure of the protein. An analogous secondary structure is generated by employing a concise homologous sequence of amino acids. The database contains 126 unique proteins, all of which are classified as non-homologous. The submitted protein sequence received a score of 70. The window width has been configured to 17, and the threshold for matching has been established at 8. The algorithm implemented in this particular context utilizes artificial neural networks for machine learning.

**mRNA secondary structure prediction:** The Mfold web server (http://www.bioinfo.rpi.edu/applications/mfold) is employed for the purpose of forecasting the secondary structure of the developed vaccine. The Mfold server effectively forecasts advantageous base pairs and structures that conform to the lower bound thermodynamic limit of ϔG [82].

**Modelling, refinement, and validation of vaccine construct:** The virtual prototype of the vaccine was developed utilizing the Robetta online application, available at https://robetta.bakerlab.org. The previously mentioned platform employs a deep learning-based methodology. Following that, the model was optimized through the utilization of the GalaxyRefine web server. ProSA-web (https://prosa.services.came.sbg.ac.at/prosa.php), the Ramachandran plot, and ERRAT (https://saves.mbi.ucla.edu/) were the three distinct methodologies employed to validate the 3D model. The ERRAT web server additionally evaluated non-covalent interactions. Amino acids that are energetically permissible or impermissible are assessed using the Ramachandran plot [83]. The vaccine model was graphically represented utilizing UCSF Chimera, an all-encompassing software application for the analysis and visualization of atomic structures and their associated data [84].

**Modelled vaccine immunological evaluation:** An evaluation of the vaccine architecture was conducted to identify the direct (continuous) and conformational (discontinuous) B-cell epitopes. Through the utilization of the BcePred webserver (http://crdd.osdd.net/raghava/bcepred/), persistent B-cell epitopes were identified [85]. Additionally, the determination of B-cell epitopes was performed utilizing ElliPro (http://tools.iedb.org/ellipro/31) [86].

#### 2.2.6 Vaccine model TLR4 receptor docking.

A molecular docking algorithm was implemented, incorporating a scoring function, to predict molecular interactions and binding energies. By means of prediction, the HADDOCK 2.2 web server (https://wenmr.science.uu.nl/haddock2.4/) has produced 10 models of the vaccine receptor TLR4 complex and 10 models of the vaccine and TLR2 complex. The ability of toll-like receptors (TLRs) to recognize microbial components within the human body has been demonstrated [87]. The top cluster model is the most reliable among the top ten models, as determined by the HADDOCK analysis. As the Z-score indicates the degree of deviation of the cluster from the mean, a more negative value corresponds to a higher level of performance. The model that exhibited the highest level of robustness among the models [88]. The binding affinity of the vaccine-TLR2 and vaccine-TLR4 complex was estimated by employing the PRODIGY webserver (http://milou.science.uu.nl/services/PRODIGY). It evaluates the physiological interactions between proteins and the intensity of binding in biological complexes on the basis of the structure of the protein-protein complex. Subsequently, COCOMAPS 2.0 webserver (https://aocdweb.com/BioTools/cocomaps2/) was used to perform a detailed analysis of interatomic contacts at the vaccine-TLR interface in our docked complex. This tool generated comprehensive color-coded 2D and 3D contact maps that highlight stable binding hotspots, enabling clear visualization of residue-residue interactions across the interface [89].

#### 2.2.7 Disulfide engineering modeled vaccine.

Geometric validations demonstrated that disulfide engineering stabilized the structure of the vaccine. By modifying cysteine residues in the dynamic core of the protein, disulfide engineering can generate novel three-dimensional protein crosslinks. Therefore, DbD2 (http://cptweb.cpt.wayne.edu/DbD2) was implemented [90].

#### 2.2.8 Molecular dynamics (MD) simulation.

**All-atom MD simulation on Desmond:** The effectiveness of the receptor-ligand interaction can be assessed using molecular dynamics (MD) modelling. [91]. The Desmond software was utilised to perform molecular dynamics simulations of the vaccination model, the vaccine-TLR4 complex, and the vaccine-TLR2 complex. During the 100-nanosecond duration of the experimental method, 1000 frames were attached to the trajectories within a 100-picosecond cycle interval. The SID application was utilised to examine the trajectory, yielding reported data that encompasses root-mean-square deviation (RMSD), root-mean-square fluctuation (RMSF), and secondary structural elements (SSE).

**All-atom MD simulation on GROMACS:** Protein and protein-ligand complex microscopic stability is assessed using molecular dynamics, an advanced automated simulation method. Structure, function, fluctuation, interaction, and behavior are demonstrated to do this. The behavior of the four complexes was investigated using GROMACS version 2023.1 for MD calculations [92]. CHARMM General Force Field parameterized protein content. The SwissParam server implemented ligand topologies [93]. The structures were vacuum minimized 2500 times using steepest descent to address steric issues. The SPC water model solvated the structure. After adding Na+ and Cl- ions, the gmx genion instrument balanced the system. The solvation process was done to ensure the system's electrical neutrality. After minimization, the MD simulation was conducted in production, NVT, and NPT ensembles. Two phases balanced the systems. First, a 100-picosecond NVT equilibration was done to maintain particle number, volume, and temperature. The procedure aimed to raise the system temperature to 300 kelvin. The second stage involved a precise 100-picosecond NPT equilibration to achieve homogeneity in temperature, pressure, and particle number. It was essential to maintain system density and pressure. The protein group's location was limited by bond limitations on all bonds during simulations. The system’s entropy decreased because NVT and NPT restricted the water molecules around the protein, relaxing them. Parrinello-Rahman barostat method and v-rescale thermostat for molecular dynamics [94]. The thermostat and barostat were adjusted for 100 picoseconds. To constrain covalent bonding, the Linear Constraint Solver application was used. Chemical bond interactions were handled using the sophisticated Particle-Mesh Ewald (PME) approach. Every system has a 100-ns production run after equilibrium.

#### 2.2.9 iMODS server MD simulation of vaccine-TLR4 complex.

Additional research was undertaken to examine the cooperative motions of the vaccine-TLR4 complex by employing the iMODS platform (https://imods.iqfr.csic.es/) [95]. By employing a unique NMA (Normal Mode Analysis) technique for dihedral design, an enhanced combined-phase representation, and multi-scale structure assembly, this web server's usability is increased. The iMODS software enables the execution of the standard mode analysis (NMA) model, which computes the intrinsic parameters of the vaccine-TLR4 and vaccine-TLR2 complexes to evaluate their stability. A range of parameters, including deformability, the main-chain elastic network model, the covariance matrix, the B-factor, and the variance, can provide valuable information regarding the stability of a protein [95].

#### 2.2.10 In silico vaccine cloning, codon optimization, and mRNA secondary structure prediction.

For codon optimization, the JCAT utility was utilized; a straightforward strategy was implemented to customize codon usage patterns for specific eukaryotic and bacterial species. The process of adaptation is directed by CAI values [96]. JCat determined that the nucleotide possessed a GC content of 53.095 and a CAI of 0.96. A higher level of MEV production was detected in the E. coli strain (K-12 Strain), as indicated by these results [97]. The CAI value should ideally exceed 0.8 or approach it. In order to attain maximum stability and transcription efficiency, it is possible to maintain a GC content between 30% and 70% [98]. The E. coli expression vector pET-28a (+) was utilized to duplicate the optimal gene sequence of the vaccine model. To accomplish this, restriction sites SbfI and BamHI were appended to the C- and N-termini of the code, respectively. The finalized vaccine structure was cloned into the pET-28a (+) vector using SnapGene software. The length of the vector undergoing replication was 443 base pairs of nucleotides [99].

#### 2.2.11 Simulations of the immune response to vaccine constructs.

To characterize the resistant patterns of the immune response elicited by the developed vaccine, the C-ImmSim webserver (https://kraken.iac.rm.cnr.it/C-IMMSIM/) is utilized. C-ImmSim employs two distinct methodologies to detect immunologic epitopes and autoimmune responses: machine learning and Position-Specific Scoring Matrix (PSSM) [100]. In order to perform the immunological simulation, three injections containing 1000 vaccine molecules were administered every 4 weeks. The determined values for the simulating volume, random seed, and simulation phase were 10 I, 12345, and 10 I, respectively. Multiple factors indicate a minimum interval of four weeks should elapse between two infusions. As a result, the temporal intervals of eight hours in the actual world were utilized to determine the duration of three infusions: 1, 85, and 170 [101]. A series of twelve injections was administered at 4-week intervals to examine the impact of exposure to *Staphylococcus aureus* vaccination.

## 3. Results

### 3.1 Drug-design

#### 3.1.1 Analysis of the target protein.

**Fig 2:**
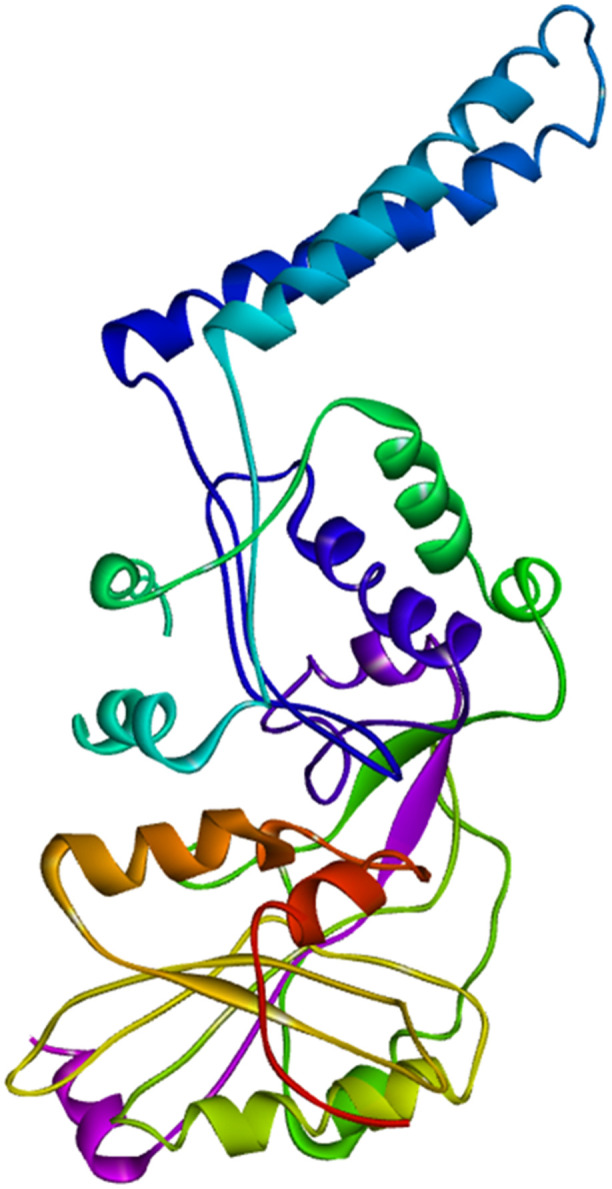
Homology model of the Aminoacyltransferase FemA protein.

**Fig 3:**
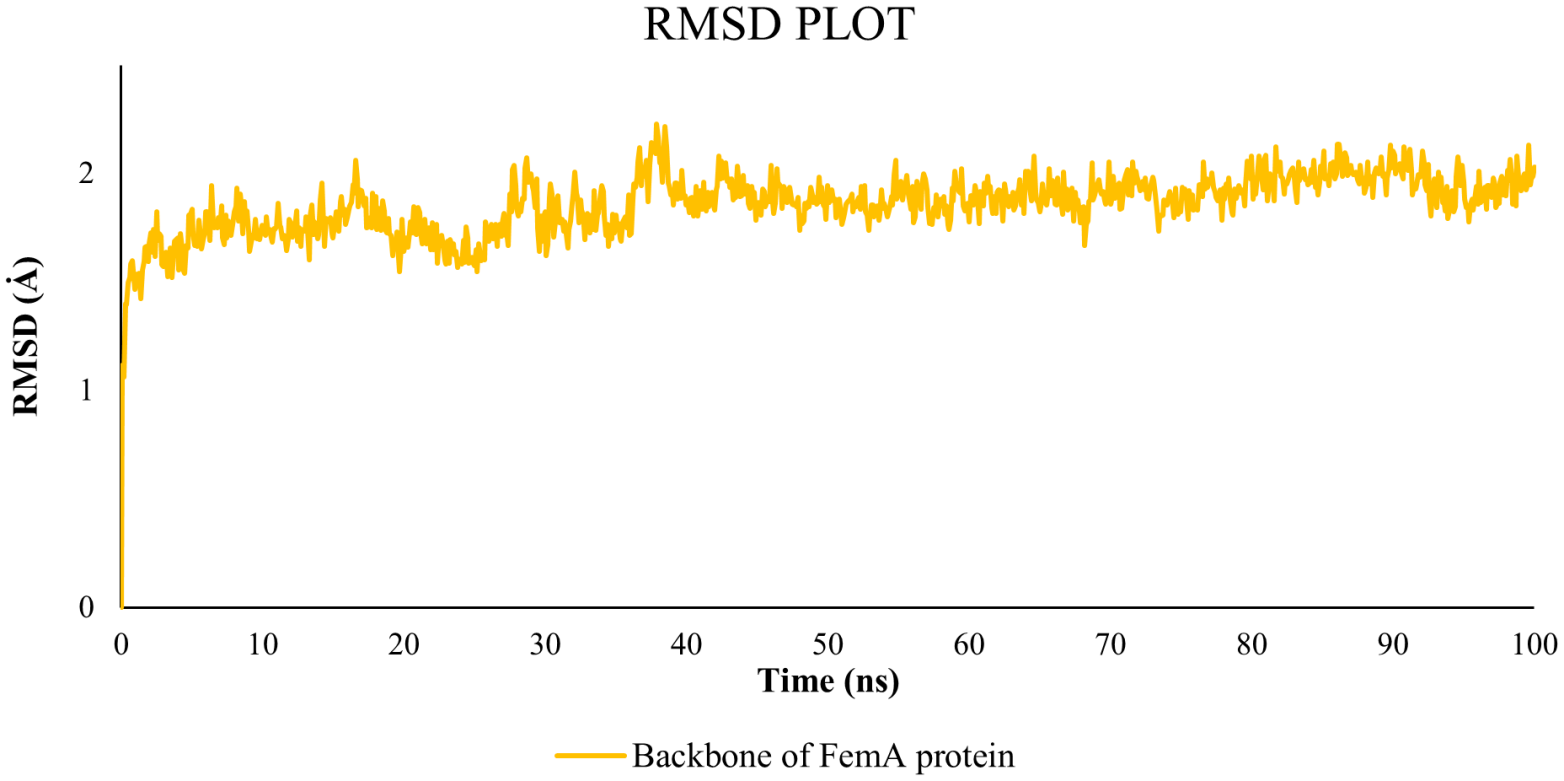
RMSD plot of the FemA protein. The graph demonstrated the deviation of the protein during the 100 ns MD simulation, generated by Desmond.

**Fig 4:**
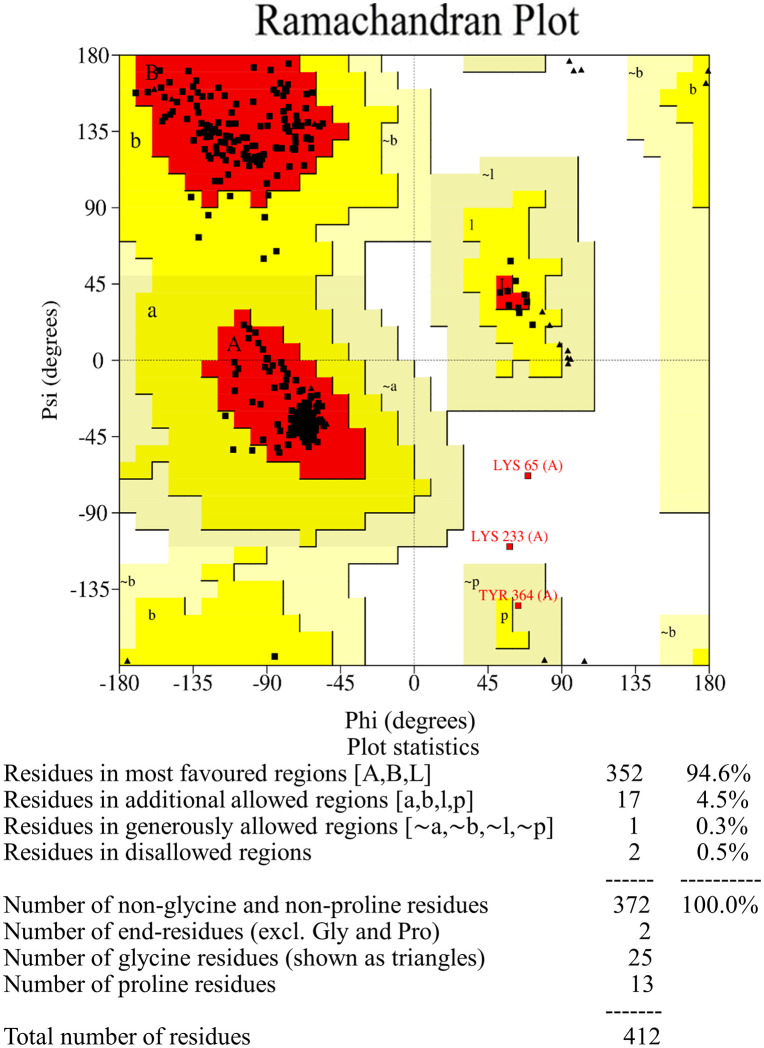
Ramachandran favours the refined model of the FemA protein. 94.6% of residues were located in the favoured region; 4.5% of residues remained in additional allowed regions; generously allowed regions contained 0.3% of residues; 0.5% of residues stayed in disallowed regions. 372 amino acids from 412 amino acid residues remained in the favored region.

**Fig 5:**
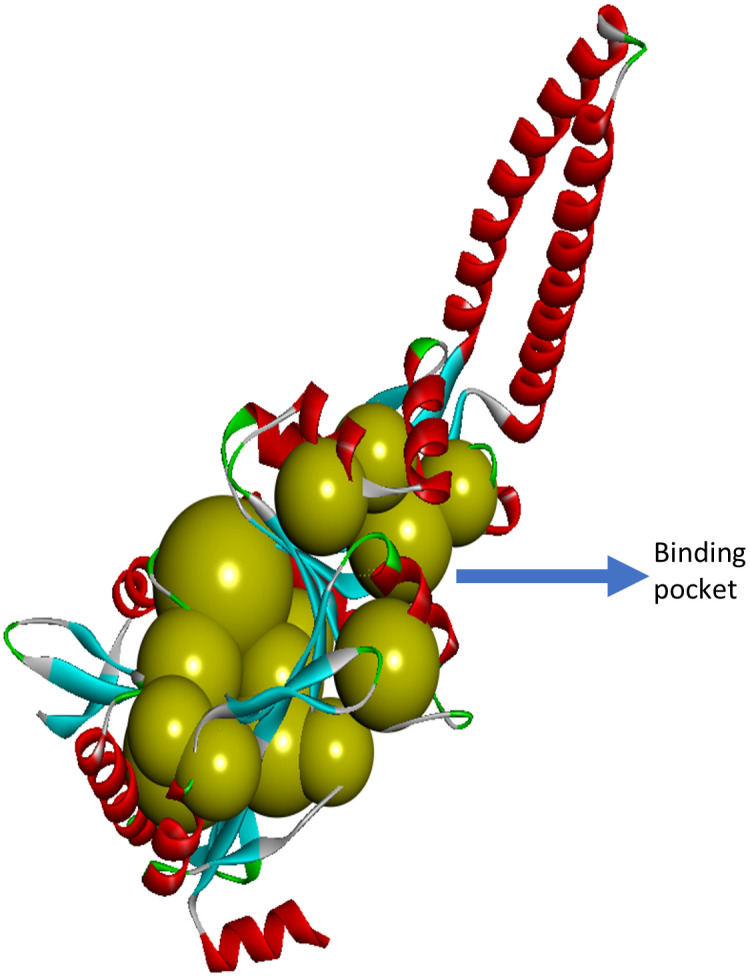
Binding pockets of the FemA protein. Binding pockets were determined and visualized by CASTp 3.0 and Biovia Discovery Studio.

A comparison between the generated model of the FemA protein using SWISS-MODEL and the predicted model of the same protein by the AlphaFold server is described in Table 1. The SWISS-MODEL-based structure was selected due to its well-established template (Fig 2) [102]. After letting the model for refinement, the GalaxyWeb server produced five refined models (Table 2). Among them, MODEL 1 showed a lower MolProbity score and a higher Ramachandran favoured region, and 0.682 Å was the RMSD value of the protein in the MD simulation (Figs 3 and 4). That is why MODEL 1 was selected for the preparatory process for the molecular docking, and it has several binding pockets for attaching the ligand (Fig 5).

**Table 1:** Structure assessment of the FemA protein.

Features	Generated model of the FemA protein using SWISS-MODEL	Predicted model of the FemA protein using AlphaFold
ERRAT	94.1	96.12
VERIFY3D	96.60	93.57
Ramachandran fovoured	91.1	93.4
QMEAN Value	0.55	0.91

**Table 2:** Refined models of the FemA protein generated by the GalaxyRefine server.

Model	MolProbity	Ramachandran favored
Initial	1.203	96.3
MODEL 1	1.483	99.0
MODEL 2	1.584	99.0
MODEL 3	1.618	98.3
MODEL 4	1.495	98.5
MODEL 5	1.546	98.3

### 3.2 Analysis of the phytocompounds

One thousand one hundred phytocompounds from 54 medicinal plants of Bangladesh were retrieved from the NPASS database, and their chemical structures were obtained from PubChem. Among them, 256 phytocompounds exhibited pharmacokinetic properties and violated none of the rules of Lipinski, Ghose, Veber, Egan, and Muegge (S1 Table). 111 of 256 phytocompounds were free from toxicity, including AMES toxicity and Hepatotoxicity. Additionally, all ligands were water-soluble and were able to pass through Caco-2 cell lines. In addition, nearly all compounds were able to pass through Caco-2 cells (S2 Table). These compounds were selected for the molecular docking.

### 3.3 Analysis of the result of molecular docking

The results of the molecular docking of antibiotics with the FemA protein are demonstrated in Table 3. The antibiotic (Doxycycline) with the maximum binding affinity score of −8.2 kcal/mol was determined as the control. A total of one hundred and eleven different phytocompounds were put through the Autodock-Vina screening process in order to identify the most effective binding relationships between ligand and protein, which are better than the control (S3 Table). Of the 111 compounds, only 7 compounds showed greater binding affinity than the control (Table 4). Furthermore, 4 compounds demonstrated better outcomes than the control, as indicated by full fitness and estimated ΔG scores. Moreover, all the ligands have more or the same number of hydrogen bonds compared to the control, except Nimbolide (Table 5). Those four compounds (17-Epi-17-Hydroxyazadiradione, Nimbolide, Pau-lownin, Nimbinin) were selected for the MD simulation.

**Table 3:** Docking scores between the FemA protein of *Staphylococcus aureus* and commercially available antibiotics for the organism.

Control	Read-1 (kcal/mol)	Read-2 (kcal/mol)	Read-3 (kcal/mol)	Average (kcal/mol)
Doxycycline	−8.3	−8.2	−8.1	−8.2
Linezolid (Zyvox)	−6.5	−6.7	−6.6	−6.6
Trimethoprim	−5.3	−5.7	−5.4	−5.47

**Table 4:** Docking scores for the FemA protein of *Staphylococcus aureus* and phytocompounds by AutodockVina.

SL	Phytocompounds	Read-1 (kcal/mol)	Read-2 (kcal/mol)	Read-3 (kcal/mol)	Average (kcal/mol)
1	Nimbinin	−8.6	−8.6	−8.6	−8.6
2	Epoxyazadiradione	−8.6	−8.6	−8.6	−8.6
3	Dehydrodeguelin	−8.2	−8.7	−8.5	−8.47
4	17-Hydroxyazadiradione	−8.5	−8.3	−8.5	−8.43
5	17-Epi-17-Hydroxyazadiradione	−8.4	−8.3	−8.4	−8.37
6	Nimbolide	−8.2	−8.2	−8.3	−8.23
7	Paulownin	−8.3	−8.2	−8.1	−8.2

**Table 5:** Docking scores for the FemA protein of *Staphylococcus aureus* and phytocompounds using Swissdock.

SL	Phytocompounds	Full Fitness (kcal/mol)	Estimated ΔG (kcal/mol)	Hydrogen bonds	Interacting amino acids
1	17-Epi-17-Hydroxyazadiradione	−2744.51	−8.21	2	VAL236, SER197
2	Nimbolide	−2750.08	−7.89	1	ARG360
3	Paulownin	−2778.26	−7.78	3	ARG337, TYR327, TYR343
4	Nimbinin	−2531.66	−7.6	2	ASP150, GLN154

Although the absolute docking score differences were modest (<0.5–1.0 kcal/mol), post-docking refinement through MD stability assessment and MM-GBSA rescoring provided additional discriminatory power, which was therefore weighted more heavily in candidate prioritization.

### 3.4 Analysis of the result of the molecular dynamics (MD) simulation and post-MD simulation MM-GBSA

**Fig 6:**
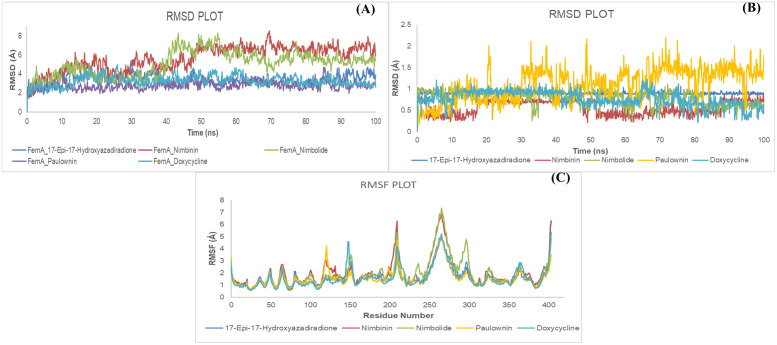
Illustration of simulation curves. The graphs show the characteristics of **(A)** RMSD values of the protein backbone relative to ligands, **(B)** RMSD of ligands, and **(C)** RMSF at 100 ns of MD simulation using Desmond software.

The analysis of the MD simulation yielded data concerning the RMSD and RMSF values, along with insights into ligand behavior and protein-ligand interactions. The analysis of the RMSD result shows how stable the protein-ligand complexes are. The average RMSD plots for the backbone of FemA in its interactions with 17-Epi-17-Hydroxyazadiradione, Nimbinin, Nimbolide, Paulownin, and Doxycycline showed values of 3.413 Å, 5.623 Å, 5.05 Å, 2.78 Å, and 3.255 Å, respectively (Fig 6A). Over the entire 100-nanosecond simulation period, the curve change remained below 3.00 Å, demonstrating a stable protein-ligand interaction [39]. The ligands 17-Epi-17-Hydroxyazadiradione, Nimbinin, Nimbolide, Paulownin, and Doxycycline exhibited a stable conformation with protein, as indicated by the average RMSD values of 0.89, 0.56, 0.8, 1.11, and 0.8 Å, respectively, as depicted in Fig 6B. In addition, most of the ligands showed lower average RMSD values than the control. Similarly, FemA_17-Epi-17-Hydroxyazadiradione, FemA_Nimbinin, FemA_Nimbolide, FemA_Paulownin, and FemA_Doxycycline had average RMSF values of 1.634, 1.947, 1.912, 1.594, and 1.613 Å, respectively (Fig 6C).

**Fig 7:**
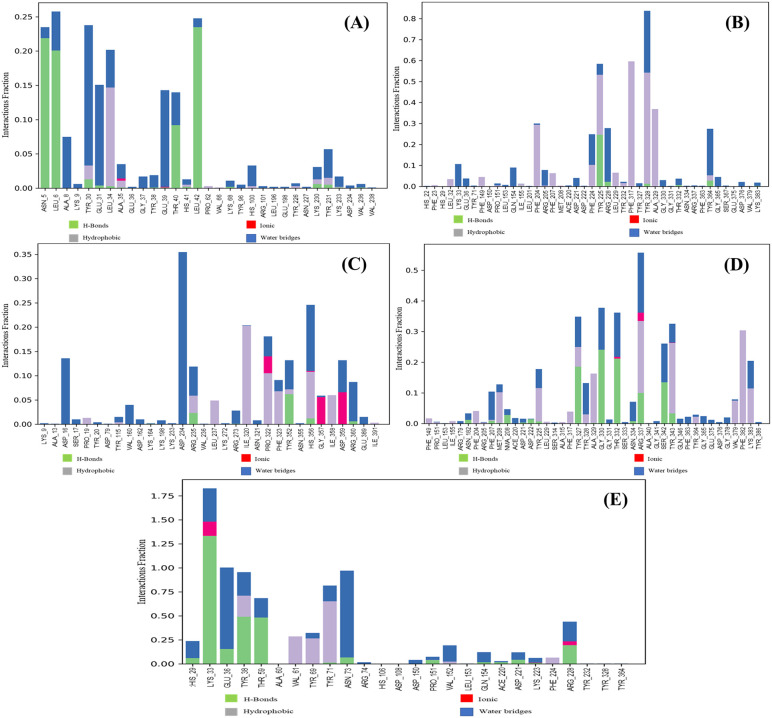
Histogram of the interaction of the FemA and ligands. **(A)**, **(B)**, **(C)**, **(D)**, and (**E**) indicated the complex of the FemA with 17-epi-17-Hydroxyazadiradione, Nimbinin, Nimbolide, Paulownin, and Doxycycline, respectively.

Over the course of 100 ns, non-covalent protein-ligand interactions were assessed (Fig 7). 17-epi-17-Hydroxyazadiradione interacted with the FemA, comprising ASN5, LEU6, TYR30, LEU34, and LEU42, etc., and these interactions persisted for more than 20% of the 100 ns time frame. Another ligand, Nimbinin, showed interactions with TYR225, PHE317, TYR328, etc., and these interactions persisted for more than 60% of the 100 ns time of the simulation. Regarding Nimbolide and Paulownin, 2 (ASP234, HIS356) and 6 (TYR327, GLY330, THR332, ARG337, TYR343, and PHE382) interactions stayed more than 25% of the total simulation time. The control showed more non-covalent interactions. The highest interaction occurred at 175% of the simulation time (LYS33), and it is normal when some protein residues can make multiple ligand-protein contacts. Three interactions (GLU36, TYR38, ASN73) existed for more than 90% of the simulation period. In post-simulation MMGBSA, the average scores of FemA_17-epi-17-Hydroxyazadiradione, FemA_ Nimbinin, FemA_ Nimbolide, FemA_ Paulownin, and FemA_ Doxycycline were −11.0582 ± 9.55, −42.7148 ± 5.03, −34.0305 ± 4.98, −39.7368 ± 6.05, and −28.1492 ± 5.90, respectively. The range of the ΔG Bind scores was also demonstrated in Table 6.

**Table 6:** Post-simulation MMGBSA-based binding free energy. All selected ligands showed higher affinity except 17-epi-17-Hydroxyazadiradione.

Complex	MM-GBSA (kcal/mol)	
	ΔG _Bind_	ΔG _Bind_ range
FemA_17-epi-17-Hydroxyazadiradione	−11.0582 ± 9.55	−25.2136 to 1.6529
FemA_ Nimbinin	−42.7148 ± 5.03	−55.1166 to −32.5269
FemA_ Nimbolide	−34.0305 ± 4.98	−42.4812 to −19.7569
FemA_ Paulownin	−39.7368 ± 6.05	−52.2117 to −28.2645
FemA_ Doxycycline	−28.1492 ± 5.90	−46.6840 to −14.7327

The key residues involved in Paulownin binding (ARG337, TYR327, TYR343, and PHE382) are located within the predicted active pocket of FemA identified by CASTp (Fig 5). Persistent hydrogen bonding and π-interactions within this cavity during MD simulation suggest structurally anchored engagement rather than nonspecific surface association. In contrast, the control drug exhibited a broader interaction distribution, including residues outside the central pocket. This difference in interaction topology may contribute to the comparatively favorable MM-GBSA profile observed for Paulownin.

### 3.2 Vaccine design

#### 3.2.1 Pre-vaccine design analysis.

**Protein sequence collection and antigenicity evaluation:** From the NCBI GenBank (https://www.ncbi.nlm.nih.gov/genbank/), we obtained the FemA protein sequence of *Staphylococcus aureus*.

**Evaluation of physicochemical properties of the selected protein:** An antigenic property score of 0.86 was utilized to identify epitopes by the VaxiJen server [63]. A range of parameters was examined utilizing ProtParam. The results indicated that the protein, comprising 420 amino acids and having an atomic weight of 49123.66 Dalton, possessed favorable antigenic properties. It was determined that the stability of every protein associated with nucleocapsid glycoprotein is below 40, suggesting that every grouping demonstrates stability. A comprehensive summary of the physicochemical properties is provided in Table 7.

**Table 7:** Antigenic and physicochemical properties of the FemA protein. The parameters were predicted using the ExPASy ProtParam tool and the VaxiJen v2.0 server.

Target Protein	Molecular Weight	Instability Index	Aliphatic Index	Theoretical Isoelectric Point	No. of Amino acids	Extinction Co-Efficient	Estimated half-life:	GRAVY
FemA	49123.66	38.67	74.52	6.94	420	52845	30 hours (mammalian reticulocytes, in vitro). >20 hours (yeast, in vivo). >10 hours (Escherichia coli, in vivo).	−0.615

**Prediction of CTL epitopes:** Cytotoxic T lymphocytes are capable of eradicating cells that are infected with viruses and bacteria, while also eliciting a robust cellular immune response. Immunogenic CTL epitopes were predicted by the NetCTL 1.2 server [66]. An analysis was conducted on the allergenic, antigenic, and immunogenic properties of the sequences. A total of twenty-four epitopes (S4 Table), from which five epitopes were determined to be suitable, underwent screening by a series of important criteria, such as antigenicity, allergenicity, toxicity, homology, and immunogenicity using online servers, to be utilized in vaccine modeling (Table 8).

**Table 8:** Final predicted CTL epitopes and their physicochemical properties.

SL No	Epitope	Supertypes	Combined Score	Antigenicity	Allergenicity	Toxicity	Homology	Immunogenicity
1	GYNAEIIEY	A1	0.6871	0.8839	No	Non-toxin	Non-homologue	0.43087
2	NQELVHFFF	A1	0.6114	1.1277	No	Non-toxin	Non-homologue	0.25919
3	FFINPFEVV	B8	0.6915	1.3766	No	Non-toxin	Non-homologue	0.24471
4	LQEEHGNEL	B39	1.3922	1.1759	No	Non-Toxin	Non-homologue	0.24969
5	TEDAEDAGV	B44	0.7266	1.1469	No	Non-Toxin	Non-homologue	0.21777

**Prediction of HTL epitopes:** Helper T-lymphocytes are part of both innate and adaptive immunity [103,104]. These entities are distinguished by the presence of an HTL epitope specific to their receptor. The FemA protein sequence was uploaded to the NetMHCII pan 3.2 servers at the time of writing [105]. Epitopes with decreased immunogenicity are distinguished by a reduced IC50 values and percentile ranks, as detailed in Table 9. The affirmative score obtained from the IFN server suggests that the epitopes specified possess the capability to induce IFN-gamma stimulation [71]. A series of important criteria was maintained, such as antigenicity, allergenicity, toxicity, homology, immunogenicity, IL-4, and IL-10 online servers. A total of forty-eight epitopes (S5 Table) were selected, of which only five epitopes were finalized for vaccine model production in accordance with the established criteria for epitope selection as a whole (Table 9).

**Table 9:** Predicted final HTL epitopes and their physicochemical properties.

SLNo.	Epitope	Antigenicity	Allergenicity	Toxicity	Homology	IL-4	IL-10	Immunogenicity
1	NELPISAGFFFINPF	0.7032	No	Non-toxin	Non-homologue	Yes	Yes	0.075788993
2	LPISAGFFFINPFEV	0.9504	No	Non-toxin	Non-homologue	Yes	Yes	0.60314136
3	ELPISAGFFFINPFE	0.6442	No	Non-toxin	Non-homologue	Yes	Yes	0.36339264
4	ISAGFFFINPFEVVY	0.8806	No	Non-toxin	Non-homologue	Yes	Yes	0.89980367

**Identification of B-cell epitopes:** B-cells are essential components of the humoral immune response. The interatomic interactions among LBL receptors lead to the formation of antibodies and the progression towards sustained resistance [106]. On the plane of supplementary proteins chosen for their non-allergenic and antigenic characteristics, which align with the MHC epitopes, the LBL is prominently displayed. The prediction of B-cell epitopes in the FemA protein was performed using the BepiPred server. Potential B-cell epitopes were identified at the individual level for epitopes that exceeded a score threshold of 0.50. Seven FemA protein epitopes (S6 Table) were identified according to the predetermined criteria, and two were selected based on their high antigenic scores (Table 10).

**Table 10:** Predicted LBL epitopes and their physicochemical properties.

SL No	Epitope	Probability Score	Antigenicity	Allergenicity	Toxicity	Homology	Immunogenicity
1	KNMDGLRKRNTK	0.8546	1.444	No	Non-Toxin	Non-Homologue	0.50804562
2	DGLRKRNTKKVK	0.8186	1.587	No	Non-Toxin	Non-Homologue	0.65322953

**Fig 8:**
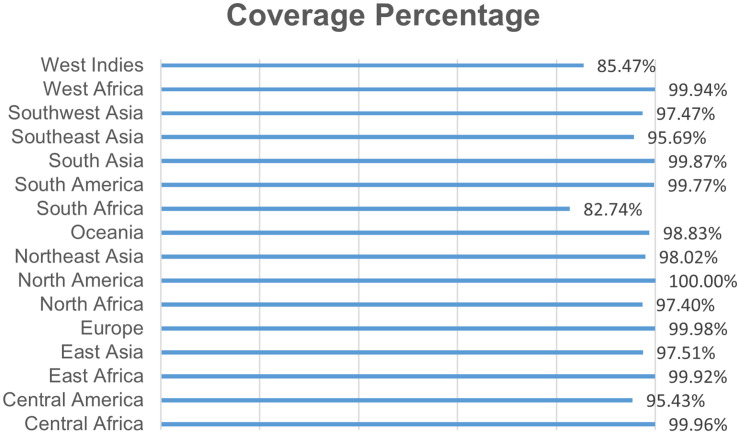
Population coverage of the constructed vaccine.

**Autoimmunity, population, and preservation analysis:** All selected CTL, HTL, and B-cell epitopes showed no significant similarity to human proteome sequences in BLASTp screening, reducing predicted cross-reactivity risk. The preservation evaluation revealed that the auxiliary proteins of interest encompassed all specified T-cell and LBL epitopes at 100%. Given the global prevalence of *S. aureus* infections across hospital and community settings, broad HLA population coverage was prioritized rather than region-specific tailoring. The population coverage analysis revealed that T-cell epitopes represented more than fifty percent of the global population. [77]. All identified epitopes with population coverage over 50% demonstrated no similarity with proteins present in humans. The distribution of MHC HLA alleles varies across numerous geographical regions globally. Therefore, it is essential to incorporate population coverage into the development of a prospective operational vaccination program. As shown in Fig 8, the highest population exposure to the vaccine was observed in North America (100%), Central Africa (99.96%), West Africa (99.94%), South Asia (99.87%), and South America (99.77%). This level of population coverage accounts for approximately 80% of the world’s population.

#### 3.2.2 Vaccine design and post-vaccine analysis.

**Fig 9:**
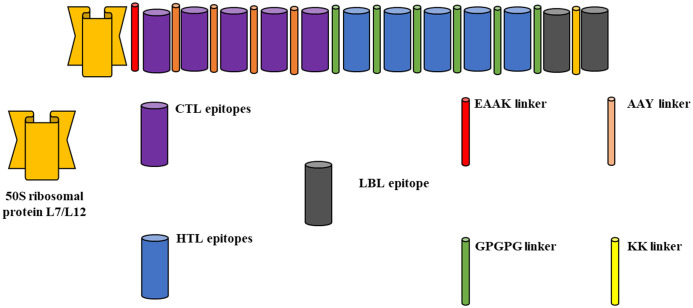
Schematic diagram of the final vaccine construct.

**3.2.2.1 Construction of final multiepitope vaccine.** The coupling of HTL epitopes with GPGPG linkers and the conjugation of CTL epitopes with AAY linker molecules and KK linkers to bind with the B-cell epitopes are described in the present study. Furthermore, by employing linker EAAAK, the association with the adjuvant Lipoprotein LprG was accomplished. Following the development of a multiepitope immunization comprising 420 amino acid residues, its antigenic, allergenic, and physicochemical properties were assessed (Fig 9).

**Fig 10:**
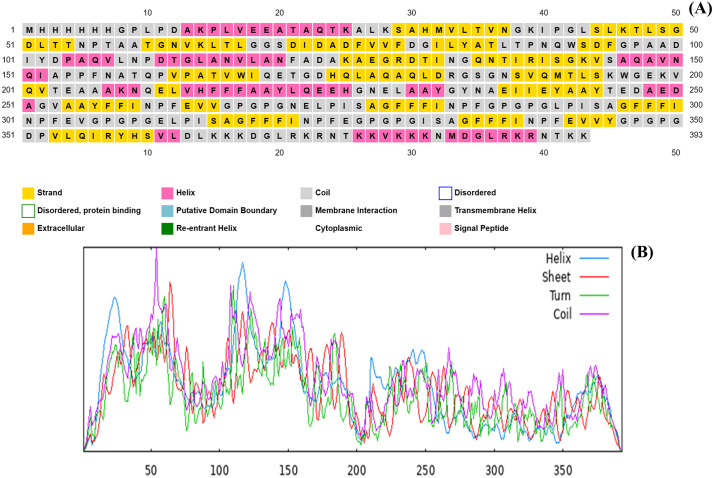
Secondary structure prediction of the constructed vaccine. (**A**) describes the outcome of PSIPRED and (**B**) illustrates the result of the SOPMA server.

**3.2.2.2 Secondary vaccine structure prediction.** The vaccine prototype simulated immune activation dynamics and was devoid of allergenic properties; its molecular weight was recorded as 49123.66 Daltons. The PSIPRED algorithm, illustrated in Fig 10A, has predicted a theoretical isoelectric point (pI) of 6.94, an aliphatic index score of 74.52, and an instability index of 38.67 [80,81]. The half-life of human reticulocytes in vitro is expected to be 30 hours, while those of yeast and Escherichia coli in vivo are expected to exceed 20 hours and 10 hours, respectively. The prediction of the secondary structure was obtained by employing SOPMA. As depicted in Fig 10B, the constructed vaccine comprised an alpha helix of 21.65%, an extended strand of 24.94%, a beta-turn of 6.87%, and a random coil of 45.65% as shown in Table 11.

**Table 11:** Structural properties of the constructed vaccine from SOPMA.

Region Name	Percentage
Alpha Index	21.65%
3_10_ helix	0%
Pi helix	0%
Beta bridge	0%
Extended strand	24.94%
Beta turn	6.87%
Beta region	0%
Random coil	45.65%
Ambiguous state	0%
Other States	0%

**Fig 11:**
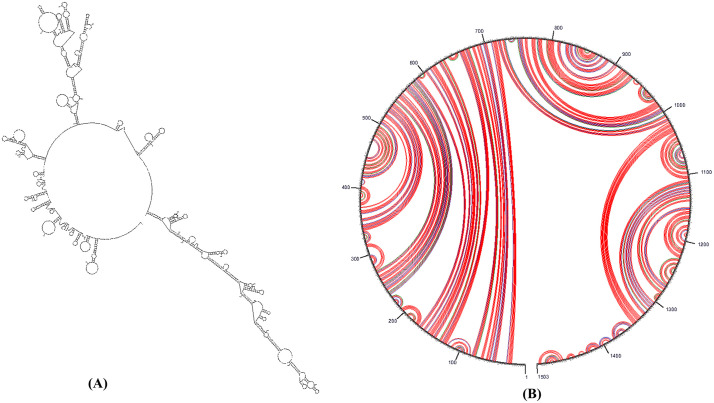
Schematic of the constructed mRNA. (**A**) describes the prediction of the RNA secondary structure of the multiepitope vaccine construct gene by the Mfold server. (**B**) shows the circle graph structure, which displays the base pairs of the structure.

**3.2.2.3 mRNA structure prediction.** As shown in Table 12, predicting mRNA secondary structure is a critical factor that can aid in the computation and interpretation of mRNA initiation, elongation, and biogenesis. Utilizing the Mfold online server, data regarding the free energy associated with the complete mRNA structure were obtained Fig 11. The score serves as an indicator of the effectiveness and resilience of the protein's analysis [107].

**Table 12:** Thermodynamics of folding of the constructed vaccine.

Structural element	δG	Information
External loop	−0.2	498 ss bases & 1 closing helices.
Stack	−0.3	External closing pair is A ^279^-V ^288^
Stack	−1.20	External closing pair is A ^280^-U ^287^
Helix	−2.40	3 base pairs.
Hairpin loop	4.12	Closing pair is V ^281^-A ^286^

**3.2.2.4 HLA allele-epitope interaction study.** After evaluating the interaction between individually chosen HTL and CTL epitopes and distinct HLA alleles, eleven bound complexes were identified. Epitopes that demonstrated an appropriate combined prediction score were recognized as binding epitopes with the corresponding HLA allele and were thus selected for incorporation into the ultimate vaccine design.

**Fig 12:**
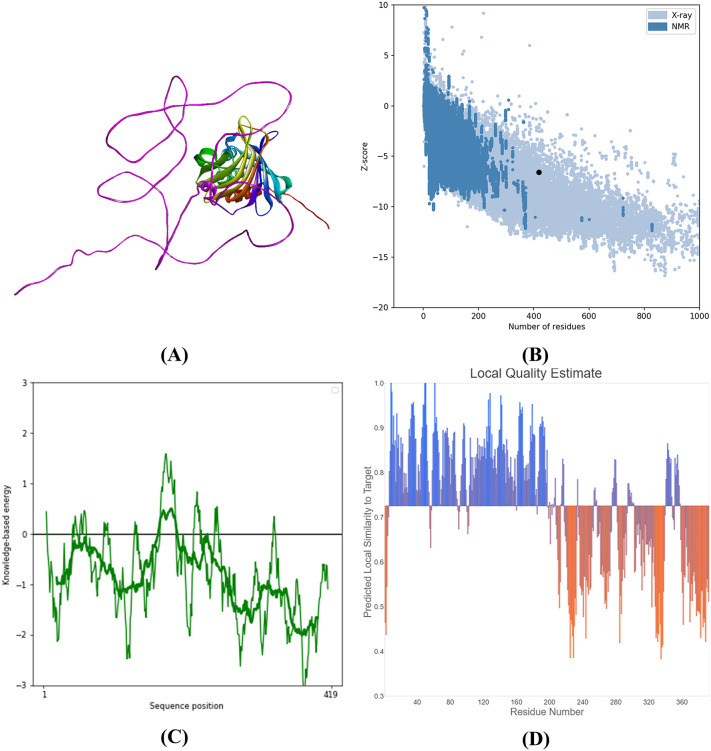
The constructed vaccine. (**A**) displays the 3-dimensional structure of the vaccine constructed by the AlphaFold server. **(B)** & (**C**) describe the ProS-web validation of the predicted vaccine construct and the ProSA-web plot of residue scores of the vaccine construct, respectively. (**D**) depicts the QMEAN4 score of the predicted vaccine model.

**Fig 13:**
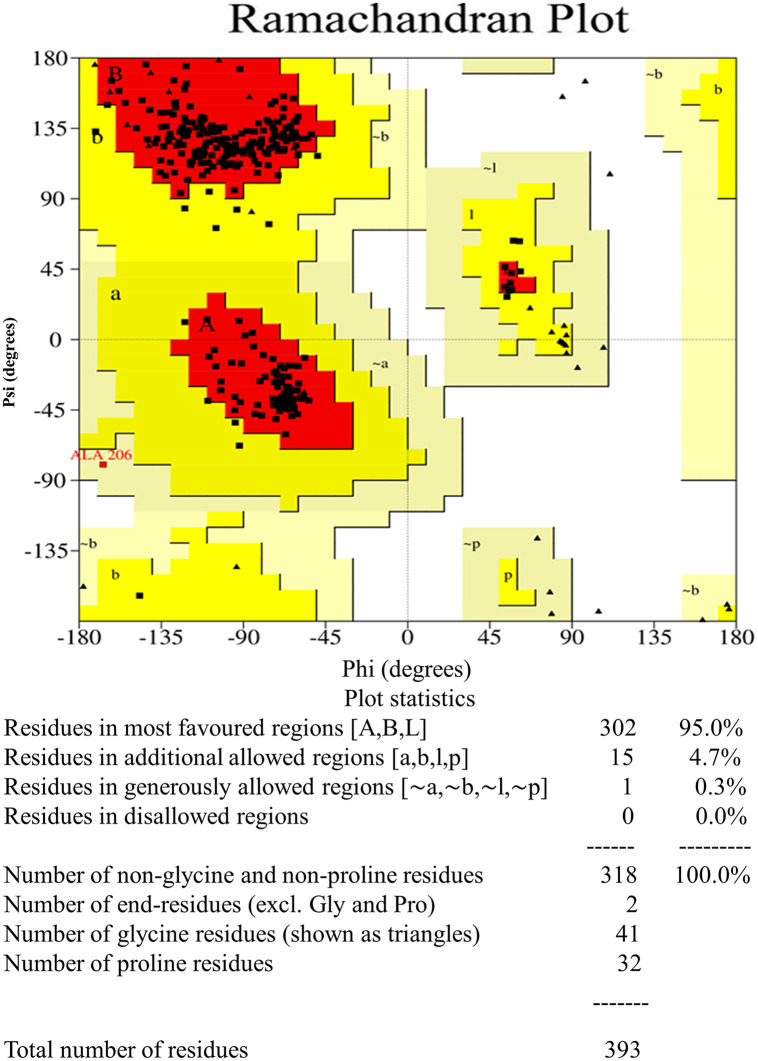
Ramachandran plot analysis of the constructed vaccine. 95.0% of residues remained in favored regions; 4.7% of residues stayed in additional allowed regions; generously allowed regions contained 0.3% of residues; 0.0% of residues remained in disallowed regions. 318 amino acids from 393 amino acid residues remained in the favored region.

**3.2.2.5 Vaccine build modelling, optimization, and verification.** After undergoing refinement on the GalaxyRefine platform, the model was validated using ProSA-web. The Z-score assigned to the model is illustrated in Fig 12, where it was −5.45. The Ramachandran plot analysis performed on the server indicated that 93.5% of the accumulations were in the favored region, 4.7% were in the permitted region, and 1.1% were exceptions, as shown in Fig 13. The physicochemical characteristics of the vaccine formulation are detailed in Table 13.

**Table 13:** Physico-chemical properties of the final multiepitope vaccine construct.

Parameter	Result
Number of amino acids	393
Molecular weight (MW)	42.184 kD
Positive residues (Arg + Lys)	31
Negative residues (Asp + Glu)	40
Theoretical isoelectric point (Theoretical PI)	5.66
Extinction coefficient (at 280 nm in H_2_O)	31400 M^-1 cm-1^
Estimated half-life (mammalian reticulocytes, in vitro)	30 hours
Estimated half-life (Yeast cells, in vivo)	>20 hours
Estimated half-life (*Escherichia coli*, in vivo)	>10 hours
Instability index (II)	20.84
Aliphatic index (AI)	78.75
Grand average of hydropathicity (GRAVY)	−0.211
Immunogenicity	Immunogenic
Antigenicity	0.7937
Allergenicity	Non-allergen
QMEAN4 value	−1.01

**3.2.2.6 Immunological property assessment.** The developed vaccine relies heavily on the effectiveness of B-cell epitopes to stimulate an immune response [108]. B-cell epitope prediction was conducted utilizing the default parameters of the BcePred online server. The factors considered in this investigation consist of hydrophilicity, antigenicity, availability, extremity, and adaptability. The conformational epitope that is found in the formulated vaccine was obtained by employing the Ellipro instrument. Analysis of persistent and atypical B-cell epitopes within the specified vaccination construct revealed potential interactions with antibodies and their versatility.

#### 3.2.3 Evaluation of the molecular docking of vaccine-TLR proteins.

**Fig 14:**
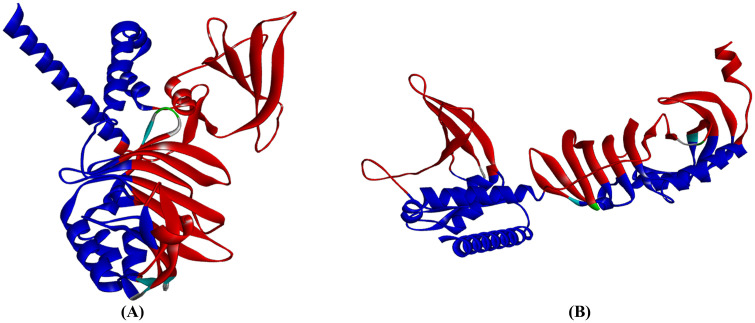
The interaction pattern of the designed vaccine with TLR2 and TLR4. Vaccine (Red) docked with receptor TLR (Blue). (**A**) showed Vaccine-TLR2 and (**B**) demonstrated Vaccine-TLR4.

**Fig 15:**
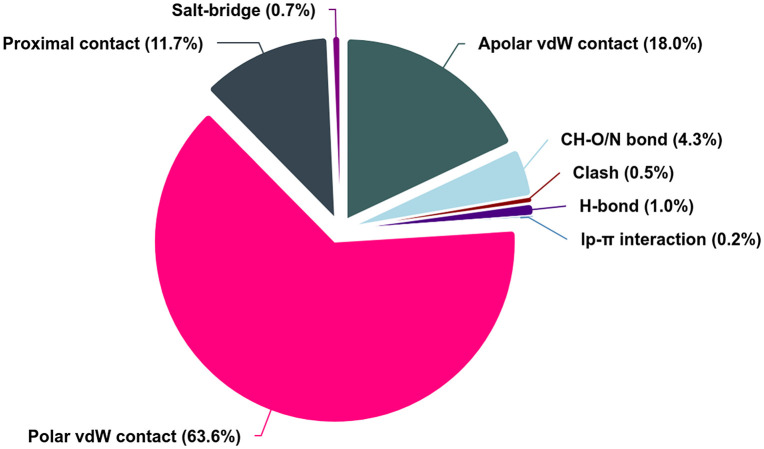
Atomic interaction distribution between TLR proteins and the vaccine.

HADDOCK 2.4 predicted models of the vaccine and TLR proteins were depicted in Fig 14. The bonding interaction between the TLR4, TLR2, and the constructed vaccine is illustrated in Tables 14 and 15. The PRODIGY server estimates that the designed vaccine and TLR2, and the designed vaccine and TLR4, have binding affinities of −16.4 kcal mol^-1^ and −14.1 kcal mol^-1^, respectively, as shown in Tables 16 and 17. The atomic interaction distribution between the TLRs and the vaccine is depicted in Fig 15. It revealed a predominantly van der Waals (vdW)-driven binding mode, with polar vdW contacts constituting the largest proportion (63.6%), followed by apolar vdW contacts (18.0%) and proximal contacts (11.7%). Specific directional interactions were present but less abundant, including CH—O/N bonds (4.3%), conventional hydrogen bonds (1.0%), salt bridges (0.7%), and minor contributions from lp-П interactions (0.2%) and clashes (0.5%).

**Table 14:** HADDOCK 2.4 server score of the vaccine-TLR2 docking.

HADDOCK score	Cluster size	RMSD from the overall lowest-energy structure	Van der Waals energy	Electrostatic energy	Desolation energy	Restraints violation energy	Buried Surface Area	Z- Score	HADDOCK score
−126.8 + /- 4.1	52	0.8 + /- 0.5	−72.6 + /- 2.9	−291.8 + /- 32.5	−7.0 + /- 2.5	112.0 + /- 39.5	2629.9 + /- 146.3	−2.3	−126.8 + /- 4.1

**Table 15:** The vaccine-TLR4 docking's HADDOCK 2.4 server score.

HADDOCK score	Cluster size	RMSD from the overall lowest-energy structure	Van der Waals energy	Electrostatic energy	Desolation energy	Restraints violation energy	Buried Surface Area	Z- Score	HADDOCK score
−115.9 + /- 23.1	9	0.8 + /- 0.6	−64.6 + /- 6.6	−482.6 + /- 89.0	23.3 + /- 3.1	219.4 + /- 26.8	2636.7 + /- 325.8	−1.7	−115.9 + /- 23.1

**Table 16:** Binding affinity and Kd prediction of vaccine-TLR2 model.

Protein-protein Complex	G (kcal mol^-1^)	K_d_ (M) at Celsius	ICs charged-charged	ICs charged-polar	ICs charged-apolar	ICs polar-polar	ICs polar-apolar	ICs apolar-apolar	NIS charger
**vaccine-TLR4**	−16.4	1e-12	6	10	19	10	47	18	24.57

**Table 17:** Binding affinity and Kd prediction of vaccine-TLR4 model.

Protein-protein Complex	G (kcal mol^-1^)	K_d_ (M) at Celsius	ICs charged-charged	ICs charged-polar	ICs charged-apolar	ICs polar-polar	ICs polar-apolar	ICs apolar-apolar	NIS charger
**vaccine-TLR4**	−14.1	4.6e-11	11	26	45	4	18	16	22.66

Complementing this, COCOMAPS 2.0 quantified the interface metrics, showing a total buried surface area upon complex formation of 3494.9 Å² (corresponding to a standard interface area of 1747.45 Å²), with 8.11% of the total accessible surface area of the isolated components becoming buried. The interface exhibited a balanced yet slightly non-polar-leaning composition, with non-polar buried area at 1912.7 Å² (54.73% of the interface) and polar buried area at 1582.3 Å² (45.27%).

#### 3.2.4 Vaccine protein disulfide engineering.

**Fig 16:**
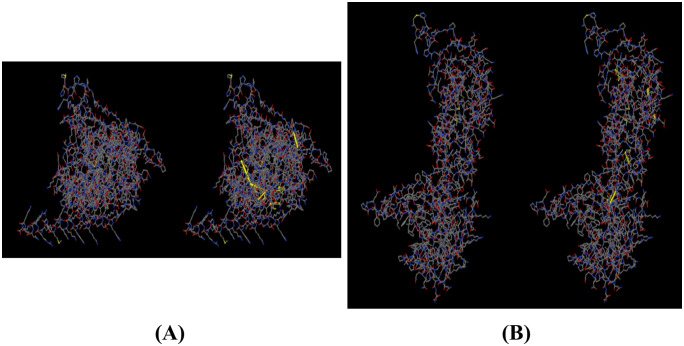
Disulfide engineering of the vaccine construct of (A) vaccine-TLR2 and (B) vaccine-TLR4 complex. The first picture exhibits the initial model without disulfide bonds, and the second shows the mutant model. The yellow stick within the circle represents the formation of a disulfide bond.

Disulfide Engineering was utilized to enhance the stability of the vaccine structure through the implementation of an inherent geometric optimization technique [90,108]. The DbD2 webserver has proposed several potential disulfide linkages involving various pairs of amino acid residues, including VAL32-THR37, PHE45-ASN64, PHE77-LEU80, ASP95-GLY120, ALA118-ALA121, PHE221-LEU253, ARG289-SER314, ARG365-PHE377, ALA581-VAL590, THR21-ALA295, THR35-VAL36, and LEU48-ASN62. Merely six of the amino acid residues, namely VAL32-THR37, PHE45-ASN64, and PHE77-LEU80, were subjected to cysteine substitution at their respective positions. Disulfide linkage formation can be initiated by assessing residues using chi3 and B-factor energy metrics, as illustrated in Fig 16. The selection of amino acids was based on a chi3 range of −87 to +97 and an energy threshold of 2.5.

#### 3.2.5 MD simulation trajectory analysis.

**Fig 17:**
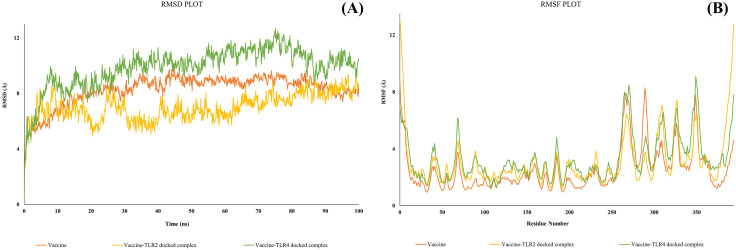
MD simulation of the constructed vaccine and docked complex of the vaccine and receptor protein. (**A**) designated as the RMSD value and (**B**) demonstrated as the RMSF value during the 100 ns simulation period.

**Fig 18:**
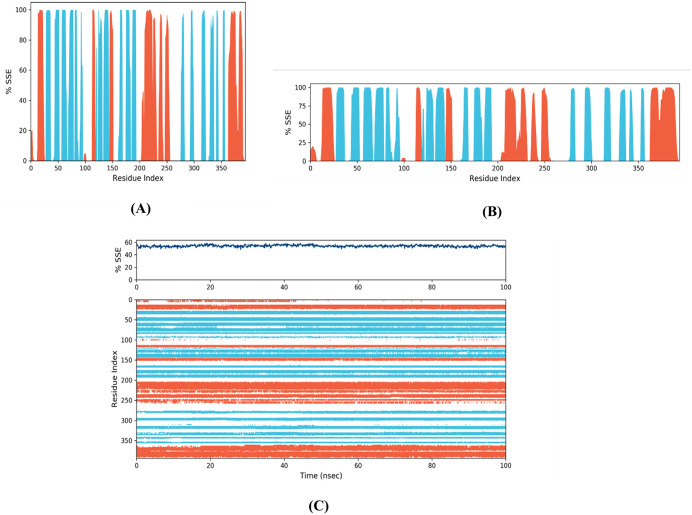
SSE analysis of the newly designed (A) vaccine-TLR4, (B) vaccine-TLR2 docked complex, and(C) Vaccine.

**3.2.5.1 MD simulation trajectory analysis of vaccine-TLR4 and vaccine-TLR2 on DESMOND.** This study conducted a molecular dynamics simulation lasting 100 nanoseconds to showcase the robustness and dynamic characteristics of the proposed vaccine alone and the vaccine model with its binding sites for TLR4 and TLR2. The vaccine-TLR4 docked complex exhibited an average RMSD of 9.863 Å, the vaccine-TLR2 complex exhibited an average RMSD of 7.014 Å, and only the vaccine model exhibited an average RMSD of 8.265 Å. Following a 40-nanoseconds simulation, the root mean square deviation (RMSD) exhibited a consistent value, providing evidence that the protein-receptor complex had achieved equilibrium (Fig 17A). In order to quantify the extent and significance of deviation in binding interaction, the root mean square fluctuation (RMSF) was computed. The vaccine-TLR4, vaccine-TLR2, and only vaccine complex exhibited an average root-mean-square fluctuation (RMSF) of 3.288 Å, 3.0811 Å, and 2.521 Å, respectively, as reported in the study, indicating the stability of the structure (Fig 17B). Furthermore, the maintenance of the vaccine TLR4 complex's stable configuration was demonstrated by monitoring its secondary structural elements (SSE) throughout the computational analysis (Fig 18). The plot of the study indicated that the structure of the vaccine exhibited prolonged interaction with the TLR4 receptor, potentially enhancing the elicitation of an immunological response. These simulations suggest a theoretical capacity to stimulate coordinated immune components under modeled conditions.

**3.2.5.2 MD simulation trajectory analysis of vaccine-TLR4 and vaccine-TLR2 on GROMACS.** The molecular dynamics trajectory parameters were evaluated via the XmGrace software. [109]. A specified initial point in the simulation is employed to compute the RMSD across all subsequent frames [110]. A comparison of values is shown in Table 18.

**Table 18:** Average values of RMSD, RMSF, SASA, Rg, and Hydrogen Bond.

Complex Name	Radius of gyration (Å)	Hydrogen bonds	RMSD (Å)	SASA (Å^2^)	RMSF (Å)
**Vaccine-TLR2**	28.48270466	165	7.52485046	22029.71075	2.42420017
**Vaccine-TLR4**	29.84898574	162	9.87407091	22692.63377	3.92988804
**Vaccine**	33.75917959	171	12.90459147	22848.36837	4.24713232

RMSD is a metric that quantifies the distance between frames. It is determined for every profile frame. The root mean square deviation of frame x is calculated.


$${RMSD}_{X}=\sqrt{\frac{\text{1}}{N}\sum_{I=\text{1}}^{N}{\left(\overset{\prime }{\underset{i}{r}}\left(t_{x}\right)\right)-r_{i}\left(t_{ref}\right)}^{\text{2}}}$$

The chosen set contains N atoms. The reference time, t ref, is typically set to the first frame, t = 0. The position of the chosen atoms in frame x, after reference frame alignment, is denoted as r.’ Frame x is associated with capture time t x. All simulation trajectory frames are handled with expertise [109]. The MD trajectory parameters were analyzed using XmGrace [110].

**Fig 19:**
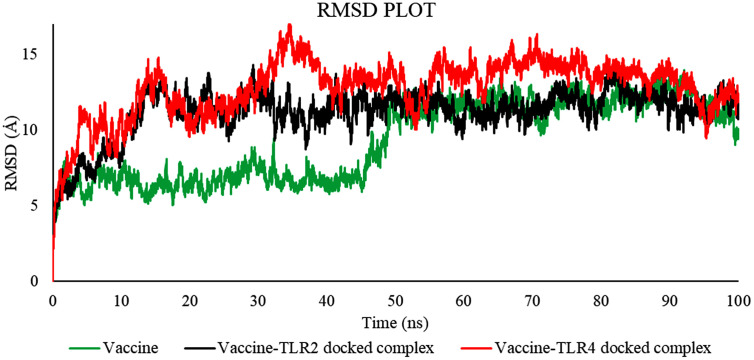
RMSD value of the vaccine-TLR2 (black), vaccine-TLR4 (red), and vaccine (green).

Fig 19 indicates that the average RMSD for vaccine-TLR2 was 7.52485046 Å, for vaccine-TLR4 was 9.87407091 Å, and for the vaccine was 12.90459147 Å, as established by the results analysis. The graph indicates that the RMSD for the vaccine-TLR2 complex ranged from 22 to 50 nanoseconds, for the vaccine-TLR4 complex from 30 to 80 nanoseconds, and for the vaccine from 25 to 70 nanoseconds.

The RMSD is useful in analyzing the time‐dependent motion of a given structure throughout the simulation [111]. Thus, a plateau in the RMSD values indicates that the structure varies around a stable average conformation, as observed in all MD simulations.

RMSF values can be used to determine local flexibility from residue displacements during the MD simulation [109]. The Root Mean Square Fluctuation (RMSF) is useful for characterizing local fluctuations along the protein chain. The RMSF for the residue is:


$${RMSF}_{I}=\sqrt{\frac{\text{1}}{T}\sum_{I=\text{1}}^{T}{\left(\overset{\prime }{\underset{i}{r}}(t)\right)-r_{i}\left(t_{ref}\right)}^{\text{2}}}$$

RMSF is determined over trajectory time T. The reference time is t ref. The position of residue i is ri, and its atoms are r’ after superposition on the reference. The angle-bracketed average square distance is calculated based on residue atom selection. Protein regions with the highest simulated fluctuation are shown by the peaks on this map. N- and C-terminal protein tails vary more than other regions.

Local flexibility can be characterized by RMSF values, derived from residue displacements during MD simulations [109].

**Fig 20:**
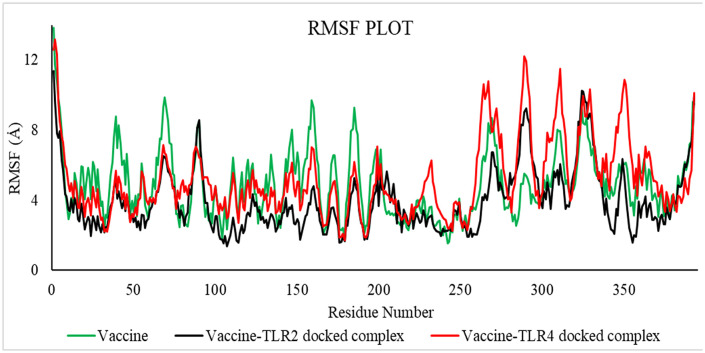
RMSF value of the vaccine-TLR2 (black), vaccine-TLR4 (red), and vaccine (green).

The RMSF is calculated from a designated simulation starting point to all consecutive frames throughout the MD session. Table 18 indicates that the average RMSF value for vaccine-TLR2 was 2.42420017 Å, for vaccine-TLR4 was 3.92988804 nm, and for the vaccine was 4.24713232 nm, as established by the results analysis. The RMSF value for the vaccine-TLR2 complex remained consistent across the residue ranges 800–900 and 3500–3700, as demonstrated in the graph. The vaccine-TLR4 complex exhibited stability within the residue range of 1000–2700, whereas the vaccine complex demonstrated stability within the residue range of 2000–2500 (Fig 20).

Movements that exhibit greater restriction concerning average positions during simulation are indicated by lower RMSF values, whilst those that demonstrate increased flexibility are indicated by higher RMSF values [111].

When mass weighting is considered, the Rg is generally defined as the root mean square distance of an atomic group from its shared center of mass [112]. The compactness of a molecular structure is indicated by the Rg, which evaluates the proximity of the atoms to the center of mass. This analysis is crucial for assessing the system's overall stability and folding characteristics over time [112].

**Fig 21:**
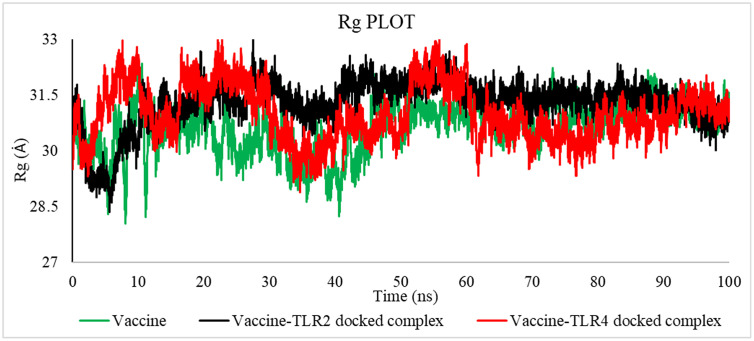
Rg of the vaccine-TLR2 (black), vaccine-TLR4 (red), and vaccine (green).

The graph indicates that the Rg values for the vaccine-TLR2 complex remained stable from 20 to 50 nanoseconds, for the vaccine-TLR4 complex from 40 to 80 nanoseconds, and for the vaccine complex from 30 to 60 nanoseconds (Fig 21). The vaccine-TLR2 exhibited an average Rg value of 28.48270466 Å, the vaccine-TLR4 displayed an average Rg value of 29.84898574 Å, and the vaccine presented an average Rg value of 33.75917959 Å, as in Fig 21.

**Fig 22:**
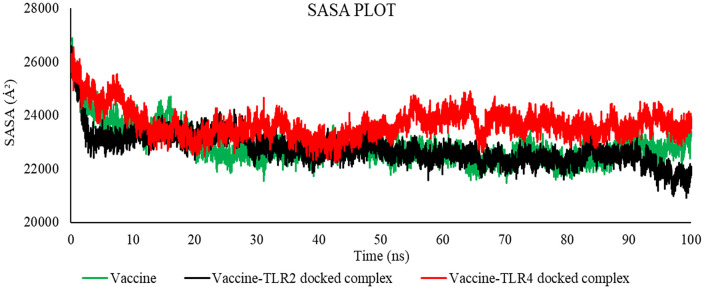
SASA of vaccine-TLR2 (black), vaccine-TLR4 (red), and vaccine (green).

The solvent-accessible surface area (SASA) refers to the fraction of a protein that is exposed to the surrounding solvent. The SASA analysis methodology establishes a framework for comprehending a protein's ability to engage in molecular interactions [112]. The SASA values for the vaccine-TLR2 complex remained stable for 40–50 nanoseconds, for the vaccine-TLR4 complex for 20–40 nanoseconds, and for the vaccine for 19–49 nanoseconds, as depicted in Fig 22. Fig 22 indicates that the vaccine-TLR2 exhibited an average SASA of 22029.71075 Å², the vaccine-TLR4 demonstrated an average SASA of 22692.63377 Å², and the vaccine presented an average SASA value of 22848.36837 Å².

**Fig 23:**
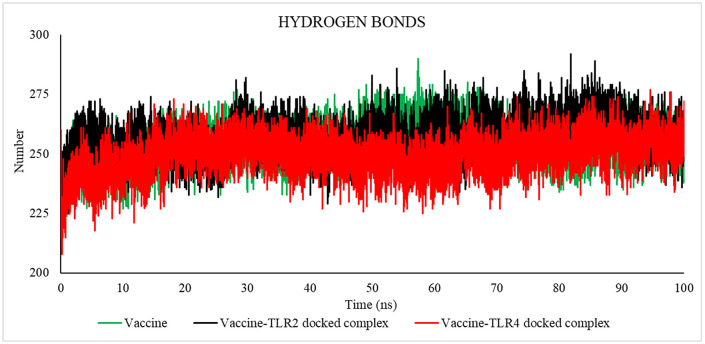
Hydrogen bond of the vaccine-TLR2 (black), Vaccine-TLR4 (red), and vaccine (green).

Hydrogen bond research can guide the modification of a lead molecule to boost activity and provide crucial information on the stability of a ligand-protein complex. H-bonds are important for the binding of ligands. Because hydrogen bond formation significantly impacts drug selectivity, metabolism, and adsorption, it is vital to consider it. Four subtypes of hydrogen bonds exist between a ligand and a protein: side-chain donor, side-chain acceptor, backbone acceptor, and side-chain acceptor. Protein-ligand H-bonds are currently defined by the following geometric parameters: 3.5 Å between the donor and acceptor atoms (D—H···A); ≥ 120° for the donor-hydrogen-acceptor atoms (D—H···A); and ≥90° for the acceptor angle between the hydrogen-acceptor-bonded atoms (H···A—X) (Fig 23).

#### 3.2.6 iMODS server MD simulation vaccine-TLR-4 complex analysis.

**Fig 24:**
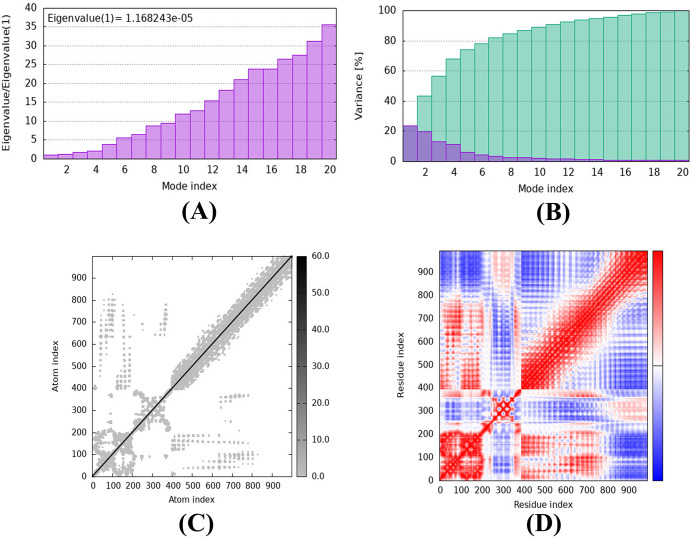
iMODS tool to analyze the MD simulation of the vaccine-TLR2 complex with (A) Eigenvalue, (B) Variance, (C) Atom Index, and (D) Residue Index.

**Fig 25:**
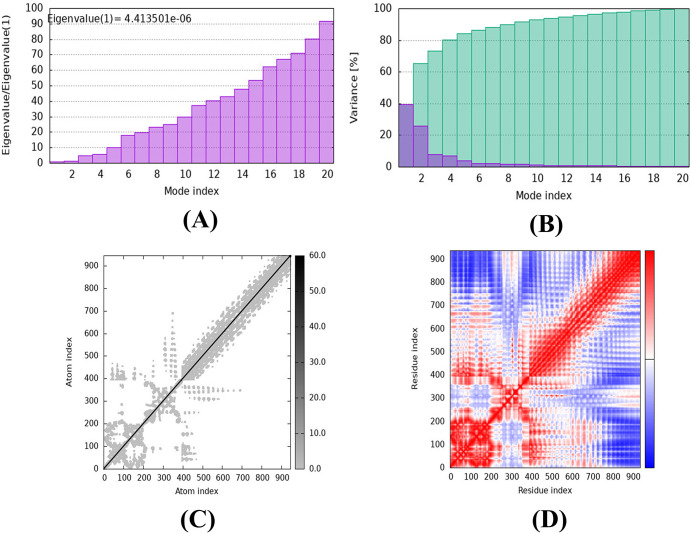
iMODS tool to analyze the MD simulation of the vaccine-TLR4 complex with (A) Eigenvalue, (B) Variance, (C) Atom Index, and (D) Residue Index.

The iMODS tool was utilized to examine the molecular dynamics (MD) simulation of the vaccine-TLR2 and TLR4 complex as part of its dynamics analysis. The Eigenvalues function quantifies the mobility of a protein, which is directly correlated with the energy required for protein elongation [113]. There is a specific energy threshold at which conformational changes in protein structures can be induced. Eigenvalues of 1.168243e-05 and 4.413501e-06 were determined to correspond to vaccine-TLR4 and vaccine-TLR2, respectively. The elastic network model and the intermolecular covariance matrix provide valuable insights into the interatomic linkages and spring-like connections that exist within a specific molecule. A steady increase in the hue of red is illustrated in Fig 24 and Fig 25, which signifies a robust correlation between amino acids linked in the vaccine-TLR-4 complex [95].

#### 3.2.7 Codon optimization and in-silico cloning.

**Fig 26:**
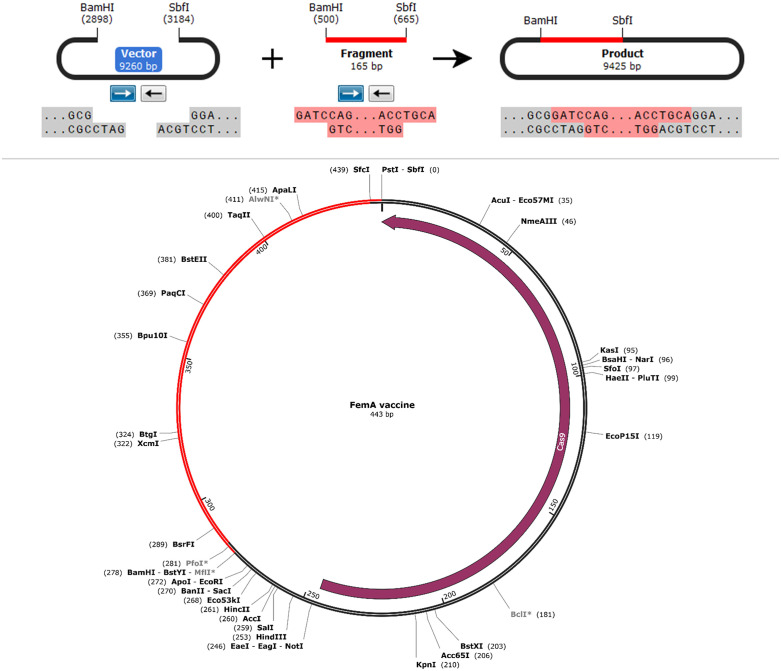
In silico cloning of vaccine. The segment represented in red is the multiepitope vaccine insert in pET-28(+) expression vector.

The codon of the novel antibody construct was optimized utilizing the Java Codon Adjustment software, which generated a codon sequence comprising 443 nucleotides [114]. The optimized sequences exhibited significantly higher expression levels, as evidenced by their CAI of 0.96 and GC content of 53.095%. By fusing the N and C termini of the modified codon sequence, the restriction enzyme restriction sequences Sbf I and Bam HI were subsequently introduced. The modified sequence-containing pET-28a(+) vector was cloned utilizing the SnapGene tool (Fig 26) [115].

#### 3.2.8 Immune simulations of vaccine construct.

**Fig 27:**
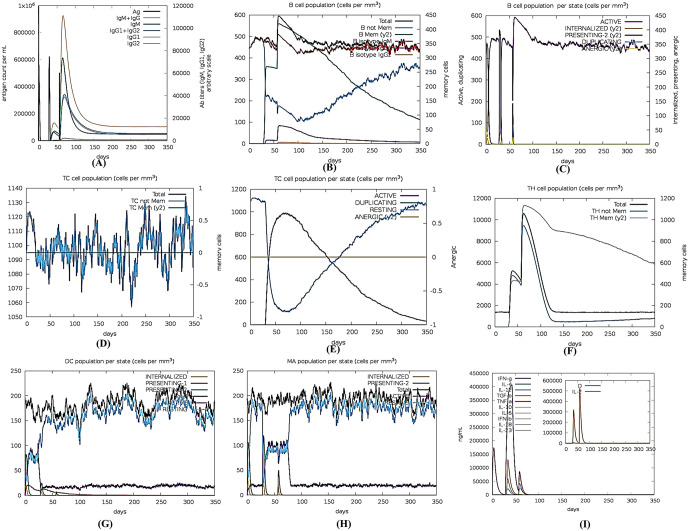
In silico simulation of immune response using the vaccine as an antigen after three subsequent injections. (**A**) Antigen and Immunoglobulins, (**B**) B-cell population, (**C**) B-cell population per state, (**D**) Cytotoxic T-cell population, (**E**) Cytotoxic T-cell population per state, (**F**) Helper T-cell population, (**G**) Dendritic cell population per state, (**H**) Macrophages population per state, and (**I**) Cytokine production.

The injection of three doses of the vaccine has demonstrated the capacity to enhance the synthesis of various immunoglobulins. Increased concentrations of IgM, IgG1, IgG2, and B-cell populations characterised secondary reactions. Antigen levels diminished following the administration of three vaccination doses. The vaccine's immunogenicity is indicated by the heightened responses of the CTL and HTL subsets, along with their corresponding memory cells, to the T-cell epitopes of the vaccine. The investigation results, depicted in Fig 27 (I-IX), demonstrate that macrophage activity escalated with each exposure, although NK-cell activity remained stable throughout the study. Subsequent exposure to the drug under investigation has resulted in higher levels of interleukin 12, interleukin 10, and interleukin 23. The antibody level peaks were similar when the vaccine was administered in doses, suggesting a potential increase in IgM + IgG and IgG1 + IgG2 levels. The graphs depicting B-cells and T-cells exhibited an increase, although IFN-gamma levels persisted at elevated levels throughout the study. The graph depicts the frequencies of B-cells and T-cells, indicating that vaccination elicited a vigorous immune response following a brief exposure period. Each successive exposure led to an enhancement in the degree of protection.

## 4. Discussion

*Staphylococcus aureus* is so dangerous that, in 2019, it caused the second-highest number of deaths from antibiotic-resistant infections [116]. It exhibits well-documented immune evasion mechanisms that coevolves with the immune system of the host and neutralizes phagocytes by inducing apoptosis or necrosis [117]. In addition, the pathogenic activities of the organism are mediated by some virulence factors. Peptidoglycan, a component of the *S. aureus* cell wall, is one of them [118]. Given the increasing prevalence of antibiotic resistance, FemA—an essential catalyst in pentaglycine bridge synthesis—was selected as a biologically validated target for both inhibitor screening and epitope prioritization [9]. Moreover, conventional methods of drug and vaccine production were expensive, time-consuming, and prone to failure [119,120]. Computational approaches provide a systematic and cost-efficient framework for hypothesis generation and early-stage prioritization in both drug discovery and vaccine design [121]. The findings of this study support the growing consensus that essential and virulence-associated bacterial proteins represent optimal targets for rational vaccine design. Previous large-scale immunoinformatics studies have shown that prioritizing proteins that are indispensable for bacterial survival, while simultaneously filtering for immunogenicity and host self-tolerance, yields highly promising vaccine candidates [7]. Our selection of the FemA is consistent with this evolutionary and functional rationale. The objective of our investigation is to utilize a structure-based drug development approach to select prospective tiny drug-like compounds and a multiepitope vaccine that could be effective against *Staphylococcus aureus* [25,122].

The use of a computer-based drug design approach is one of the most efficient methods for screening databases and identifying novel therapeutic compounds effective against multidrug-resistant *S. aureus* [123]. Additionally, identifying novel pharmacological targets is a vital step in this process. By examining the structural and functional roles of key proteins, as well as the identification of potential therapeutic targets, it is possible to develop a novel drug against the targeted protein. Furthermore, the safety, acceptability, and efficacy are improved by a variety of characteristics, including permeability, screening, and Lipinski's rule of five. A stable target protein-drug-like molecule has more chances of working on a target [124]. The rate of oral drug absorption can be accurately predicted using Caco-2 cell models derived from carcinoma cells of the colon [125]. All selected phytocompounds met the ADMET properties; showed predicted acceptable pharmacokinetic profiles with no major rule violations detection.

Identification of potential drug targets is pivotal to the drug design approach [126]. For this purpose, the FemA was considered as a target protein, and the amino acid sequences were retrieved from the Uniprot database [9]. The structural reliability of FemA was further strengthened by incorporating quantitative validation metrics and comparative modeling. The SWISS-MODEL–derived structure achieved a QMEAN value of 0.55, indicating acceptable global model quality, while the AlphaFold-predicted FemA structure exhibited a high confidence score of 0.91, supporting the robustness of the predicted fold. Among the generated models, MODEL 1 was selected for downstream docking analyses based on its superior stereochemical quality, as reflected by a lower MolProbity score, a higher proportion of residues in the Ramachandran favored region, and stable conformational behavior during molecular dynamics simulation, with an RMSD of 0.682 Å. The convergence of these validation parameters, together with the presence of multiple surface-accessible binding pockets identified by CASTp 3.0, supports the suitability of MODEL 1 for structure-based inhibitor screening, as CASTp has been widely applied for active site identification in structure-based drug discovery [127].

Additionally, the use of SWISS-MODEL templates, which preserve annotated oligomeric states and conserved ligands or cofactors, likely contributed to more realistic binding-site geometry and improved protein–ligand interactions [121], thereby providing a reliable structural framework for subsequent phytocompound docking and interaction analyses. A comprehensive phytocompound screening workflow was employed to identify potential FemA inhibitors. Initially, a total of 1,100 phytocompounds with reported antimicrobial activity from 54 medicinal plants of Bangladesh were retrieved from the NPASS database, and their chemical structures were obtained from PubChem. These compounds were first evaluated for drug-likeness using Lipinski’s rule of five, which includes the following criteria: hydrogen bond donors ≤5, hydrogen bond acceptors ≤10, molecular weight <500 Da, logP < 5, number of rotatable bonds <10, and polar surface area ≤ 140 Å² [128]. Following this initial filtering, 256 phytocompounds exhibited pharmacokinetic properties and violated none of the rules of Lipinski, Ghose, Veber, Egan, and Muegge (S1 Table) [129]. Based on acceptable pharmacokinetic and toxicity parameters, 111 of 256 phytocompounds did not indicate high toxicity risk, including AMES toxicity and Hepatotoxicity. After sequential drug-likeness and toxicity screening, compounds meeting established pharmacokinetic criteria were advanced to structure-based docking analyses. To note, ADMET predictions are probabilistic screening tools and do not substitute for pharmacological validation. Overall, of the initial 1,100 phytocompounds, approximately 19–22% passed combined drug-likeness and ADMET filters. These phytocompounds were retained and subsequently subjected to the molecular docking studies against the FemA protein.

Molecular docking was performed using AutoDock Vina, a widely used computational approach for predicting ligand–protein binding affinity and interaction modes [130]. The docking results revealed that 7 (4%) out of the 111 screened phytocompounds exhibited higher binding affinity toward the FemA than the reference drug doxycycline, which showed a binding energy of −8.2 kcal/mol. The difference in docking scores between Paulownin (−7.78 kcal/mol) and the control drug Doxycycline (−7.5 kcal/mol) is modest (0.28 kcal/mol), and docking algorithms like AutoDock Vina have inherent uncertainties (typically ±1–2 kcal/mol due to approximations in scoring functions, conformational sampling, and solvation effects) [50]. Isolated docking scores alone may not reliably predict biological activity, as they represent static snapshots and do not account for dynamic interactions or entropic contributions. Therefore, we did not rely solely on docking for candidate selection; instead, it served as an initial screening step, followed by orthogonal validation through molecular dynamics (MD) simulations (100 ns) and MM-GBSA free energy calculations to assess stability and binding energetics in a more physiologically relevant context. Several phytocompounds exhibited favorable docking scores but unstable MD trajectories or weaker MM-GBSA energies, leading to their deprioritization. This highlights that docking affinity alone was insufficient for advancement. Depending on the score of full fitness and estimated free energy (ΔG), four compounds (17-Epi-17-Hydroxyazadiradione, Nimbolide, Paulownin, and Nimbinin) were taken to the MD simulation. The simulations focused on interaction stability metrics (RMSD, RMSF, H-bond persistence) rather than detailed conformational transition pathways. Among them, two compounds, 17-Epi-17-Hydroxyazadiradione and Paulownin, showed stability, as indicated by RMSD (3.413, 2.78 Å) and RMSF (1.634, 1.594 Å) results, compared to the RMSD (3.255 Å) and RMSF (1.613 Å) results of Doxycycline. There is a consistent correlation between the hydrogen bond count and protein-ligand complex stability [131]. Moreover, the complex of FemA_17-Epi-17-Hydroxyazadiradione and FemA_Paulownin contained the same or more hydrogen bonds (2,3) than Doxycycline (2). Furthermore, post-simulation MMGBSA was performed to validate the free binding energy after simulation [39]. Nimbinin, Nimbolide, and Paulownin demonstrated higher binding energy than the control drug Doxycycline, but 17-epi-17-hydroxyazadiradione showed lower binding energy than the control. Nevertheless, additional research is required to validate the effectiveness and potency of the *in silico-*assessed phytocompounds.

While new drug development is important, numerous scientists are interested in preventing infections by developing vaccines through computational approaches [132–135]. Immediate development of an efficacious *S. aureus* vaccine is imperative due to the escalating resistance of the bacterium to existing antibiotics and the absence of a vaccine at this time. The *S. aureus* proteome was comprehensively analyzed through subtractive proteomics filters to pinpoint the most important proteins for identifying the crucial epitopes and developing the most effective multiepitope vaccines (MEVs). It is possible to develop computational vaccines that specifically target pathogenic, antigenic proteins. Pathogens require virulence proteins to successfully invade their host [136]. Homologs of human proteins were identified and eradicated in order to prevent an autoimmune reaction. Additionally, paralogous proteins, cytoplasmic proteins, and superfluous proteins were omitted [137]. For epitope selection and MEV design, extracellular and membrane proteins that are involved in pathogen adherence and pathogenicity to host cells were given preference [136]. Prominent candidates for vaccination include the FemA, which is essential for the survival of the pathogen and possesses antigenic properties. The selected protein served as the basis for epitope prediction. Importantly, previous *S. aureus* vaccine candidates targeting capsular polysaccharides, ClfA, IsdB, and surface adhesins failed in clinical trials due to insufficient functional immunity or strain variability. These strategies focused primarily on antigen identification, and relied on conventional screening pipelines that lacked cytokine-based functional validation and receptor-level interaction analysis [138]. In contrast, the present approach targets an essential intracellular enzyme involved in methicillin resistance and incorporates cytokine-induction prediction (IFN-γ, IL-4, and IL-10) to assess Th1/Th2 immune balance, together with receptor-level docking analyses as recommended in recent immunoinformatics studies [13–16], representing a mechanistically distinct strategy. Unlike classical *S. aureus* vaccine targets, FemA represents an essential enzymatic target with lower antigenic redundancy but potentially higher sequence conservation. This distinction may influence immune accessibility and should be interpreted cautiously. Building on its essential enzymatic role, sequence-defined regions of FemA were further explored for epitope derivation within a multiepitope framework. Blocking it weakens the cell wall and makes it harder for bacteria to survive. Meanwhile, the sequence-defined surface regions of FemA are used to derive CTL, HTL, and B‑cell epitopes that are antigenic, non-allergenic, non-toxic, and broadly HLA‑covered, supporting its role as a preventive antigen. Overall, the rationale for choosing the FemA is to build a unified pipeline for one experimentally validated target and also for a multi-epitope vaccine for long-term prevention and reduction of the FemA-expressing *S. aureus* burden. This dual-purpose strategy represents a conceptual advance over traditional approaches that treat vaccine development and drug discovery as independent pipelines. While the FemA is a conceptually attractive shared target for both pharmacological inhibition and epitope-based vaccination, any parallel computational exploration benefit of these approaches remains speculative and will require dedicated experimental evaluation (e.g., combined drug–vaccine regimens in infection models) to confirm additive or supra-additive effects.

Subsequently, several online databases and tools were utilized to screen potential HTL, CTL, and B-cell epitopes in candidate vaccine proteins. The antigenic epitopes that were selected and subsequently ranked in accordance with predetermined criteria for the construction of vaccine were: 1) possessing non-allergenic characteristics and the ability to stimulate an immune response, epitopes should demonstrate a robust affinity for binding to HLA class I-II molecules, 2) epitopes ought to possess the capacity to elicit responses from MHC molecules; consequently, they should exhibit extensive population coverage, and 3) epitopes ought to lack similarity to the constituents of human proteins, while B-cell peptides ought to predominate at the interface of the target protein [139,140]. Epitopes of HTL, CTL, and B-cells are all essential constituents of a successful MEV [74]. Our primary emphasis was on incorporating B-cell epitopes due to their vital role in antibody production. Although T-cell immunity typically confers permanent protection against infections, B-cell memory can rapidly generate antibodies to counter threats [141]. CTLs can restrict viral transmission by eliminating infected cells and secreting antiviral cytokines specific to the pathogen [142]. Researchers considered the conservation of B-cell and T-cell epitopes across *S. aureus* strains, as well as their allergenicity, toxicity, antigenicity, and immunogenicity, when selecting epitopes for the MEV. Helper T-cells, which produce cytokines such as IFN-gamma, IL-10, and IL-4, can inhibit detrimental inflammatory responses. Additionally, cytotoxic B-cells, T-cells, and the innate immune system are all significantly activated by helper T-cells. For candidate selection, the cytokine-producing capacity of specific HTL epitopes (i.e., IFN-gamma, IL-10, and IL-4) was also evaluated. However, no explicit structural mapping of predicted epitopes onto the 3D model was performed; therefore, conclusions regarding surface accessibility remain inferential. Across numerous ethnic groups, the HLA allele response to highly polymorphic T-cell epitopes is conserved. A greater number of alleles combined with T-cell epitopes results in enhanced population coverage.

Thus, the CTL and HTL epitopes were selected based on their corresponding HLA alleles, and an investigation was conducted to determine the extent to which these epitopes are recognized globally. From several hundred predicted CTL, HTL, and B-cell epitopes across multiple servers, only 12 passed all successive filters for high antigenicity, non-allergenicity, non-toxicity, and balanced cytokine induction. The selected epitopes and their corresponding alleles should be suitable for use across most regions of the world, according to the data. The chosen epitopes protect more than 97% of the global population coverage, meeting the criterion that the vaccine candidates must protect individuals worldwide against *S. aureus* infection. Given the high burden of *S. aureus* infections, including MRSA, in South Asia, sub-Saharan Africa, and parts of Southeast Asia (where our research groups are based), this broad HLA coverage supports potential utility in high-prevalence Low- and Middle-Income Countries’ settings [143,144]. To generate the MEV construct, appropriate linker sequences were incorporated to ensure optimal epitope presentation and structural stability of the vaccine construct. B-cell epitopes were joined using KK linkers, CTL epitopes using AAY linkers, and HTL epitopes using GPGPG linkers. These linkers are commonly employed in multiepitope vaccine design as they enhance the folding, stabilization, and expression of proteins [145]. The use of adjuvant coupling is critical for enhancing the immunogenicity of vaccines containing multiple epitopes. Adjuvants are chemical components incorporated into vaccine formulations to safeguard against infection by improving antigens’ stability, specific immune responses, durability, and growth [146]. The adjuvant incorporated into the multiepitope vaccine construct was selected based on its established immunostimulatory properties, capacity to enhance antigen presentation, and documented involvement in TLR-mediated innate immune activation. In this study, the bacterial lipoprotein LprG was employed as an adjuvant due to its well-characterized role as a pathogen-associated molecular pattern (PAMP) capable of activating antigen-presenting cells. In addition, EAAAK linkers were used that connected the promoter of the MEV to the adjuvant (LprG); this linker improves structural rigidity and reduces unfavorable interactions between adjacent protein domains, thereby supporting proper folding of the chimeric construct [147]. Structural stability was supported by disulfide engineering analysis, 100-ns MD simulations under explicit solvent conditions, and normal mode analysis demonstrating limited deformability.

By joining the adjuvant and epitope with the EAAAK linker, the two domains of the bifunctional fusion protein can be separated efficiently. In addition to improving thermal stability, this rigid linker reduces interactions between the vaccine domains of chimeric proteins [135]. The complete length of the constructed vaccine was ascertained to be 444 amino acids. Despite the considerable length of the developed vaccine, numerous experiments have demonstrated that lesser lengths can be just as practical [148]. The physicochemical profile of the MEV was subsequently examined. The vaccine’s molecular weight is 42.184 kDa, and it does not share any sequence homology with the human proteome. The high solubility of the recombinant protein, achieved through its overexpression in *E. coli*, facilitated its easy transport into the host cell. A high instability score indicates that the expressed protein will retain its functionality. It is mildly acidic, as indicated by the steady physiological pH relationship, as suggested by the MEV's calculated pI of 5.66. Furthermore, the hydrophilic and thermostable characteristics were indicated by the GRAVY score and aliphatic index, respectively. Based on previously documented data, the median half-life of MEV in yeast is over 0 hours, *in vitro* it is 30 hours, and *in vivo* it is over 20 hours [135,149–151].

In addition to being extraordinarily immunogenic and antigenic, MEV is highly adaptable, non-allergenic, and non-toxic. These results suggest that the MEV construct is predicted to elicit coordinated immune responses under modeled conditions. Tertiary structure prediction facilitates a more comprehensive understanding of protein dynamics, ligand-protein associations, and protein function [152]. By performing a 3D structure simulation of the MEV, we successfully assessed its attractive characteristics. The outcomes of implementing numerous computational strategies confirmed the superior quality of the projected structure. To achieve successful systemic delivery, candidate vaccines must establish sustained interactions with receptor proteins, such as TLR2 and TLR4. These proteins were considered primary immune receptors due to their established roles in recognizing bacterial components and initiating both innate and adaptive immune responses. Several studies have demonstrated that ligands or peptide-based vaccine constructs capable of engaging with these proteins can significantly enhance immunogenicity by activating downstream NF-κB and cytokine signaling cascades [153,154]. In the present study, the molecular docking and the molecular dynamics simulation have all showed that the multiepitope-based vaccine binds with high affinity to TLR2 and TLR4. This binding requires a negligible amount of energy, which is favorable from an energetic standpoint (−16.4 and −14.1 kcal mol^-1^). Protein-protein docking analyses of the MEV_TLR4 and MEV_TLR2 complexes unveiled the presence of numerous hydrogen bonds, whereas molecular dynamics simulations unveiled only marginal alterations. The interface stability arises primarily from numerous weak, cumulative vdW attractions, particularly polar vdW, rather than reliance on a few strong electrostatic or hydrogen-bonding anchors, rendering the complex relatively tolerant to conformational fluctuations such as those observed in RMSD during molecular dynamics simulations [155]. Subsequently, the value of the interface metrics falls comfortably within the typical range reported for stable protein-protein interfaces in vaccine-TLR docking studies (often 1500–2500 Å² interface area, with comparable polar/non-polar balances in multi-epitope constructs), supporting robust shape complementarity and sufficient contact extent for effective TLR engagement [156]. Despite favorable interface metrics and binding energies, molecular docking provides a predictive assessment of receptor engagement rather than direct evidence of downstream signaling activation. In particular, TLR4 signaling is known to depend on co-receptors such as MD-2 and CD14, which were not explicitly modeled in this study [157]. Also, direct experimental or structural evidence confirming a specific interaction between LprG and TLR4 is currently unavailable; however, molecular docking with TLR4 was performed to computationally assess the potential compatibility of the vaccine construct with an additional innate immune receptor implicated in antibacterial defense. Therefore, although the observed binding affinities suggest possible receptor engagement, experimental validation will be required for functional activation of TLR-mediated immune pathways.

Consequently, docking analyses suggest potential stable interactions between the MEV construct and TLR2/TLR4 receptors. After binding to human immune receptors and simulating its entry into the body, MEV was evaluated for its ability to imitate the host immune system. The vaccine stimulates both cellular and humoral immune responses in principle. Throughout the process of immunological simulation validation, the designed vaccine demonstrated the most significant surge in IFN production, along with notable IL-10 and IL-2 activity. The MEV construct design assumes additive immunogenicity of concatenated epitopes; however, potential epitope competition or immunodominance hierarchy effects cannot be excluded in vivo. In addition, C-ImmSim outputs represent theoretical immune simulations and do not substitute for experimental immunogenicity testing. Antibody-mediated immunity plays a critical role in host defense against S. aureus infection. Additionally, immunoglobulins that are overactive (IgM, IgG, and their isotypes) have been observed to be implicated in isotype switching. In addition to predicting a wide range of immunological responses, including numerous B-cell and T-cell epitopes, the Simpson index can be conceptualized as a subunit vaccination strategy. To optimize the expression of the vaccine protein in the *E. coli* system, investigations into mRNA secondary structure and codon optimization were conducted. Afterward, the optimized reverse-translated sequence was cloned into a functional vector by adding SbfI and BamHI restriction sites. The costly and time-consuming nature of designing and developing a potential vaccine is expected. By applying the *in silico* immunoinformatic method, it is possible to develop a vaccination that is both distinct and efficacious. However, challenges related to protein purification, post-translational modifications, insolubility, and setbacks in vaccine design are typical obstacles that may arise frequently during this procedure. The present analysis indicates that the construction of the vaccine in this study is stable, soluble, and amenable to post-translational modifications. Above all, this MEV can elicit a robust immune response while avoiding toxicological effects and allergic reactions. Furthermore, additional in vivo research and clinical trials are necessary to ascertain the effectiveness of the engineered MEV against staphylococcal infections. Beyond immunization, a major contribution of this work is the extensive phytochemical screening pipeline applied to the same FemA target. Inspired by the need for systematic and reproducible computational workflows, similar in spirit to large-scale vaccine-design platforms such as VacTarBac, we screened 1,100 phytocompounds derived from 54 medicinally important plants of Bangladesh [7,13]. Through sequential pharmacokinetic filtering, toxicity assessment, molecular docking, molecular dynamics simulations, and MM-GBSA analyses, we identified a small set of lead compounds exhibiting stable and energetically favorable interactions with FemA.

Collectively, these findings position FemA as a rational shared target for both epitope-based vaccine design and phytochemical-driven inhibitor discovery. Although the drug discovery and vaccine design arms were conducted independently, both were deliberately centered on the same biologically validated target to provide a coherent decision framework. Phytocompound binding targeted the catalytic cavity, whereas epitope selection focused on immunogenic sequence regions, reflecting complementary but biologically distinct targeting strategies rather than functional overlap. In addition, although FemA is essential for pentaglycine bridge formation and methicillin resistance expression, enzyme-targeted inhibition may theoretically impose selective pressure leading to resistance. However, because FemA performs a structurally constrained catalytic role in peptidoglycan assembly, mutational tolerance within its active region is expected to be limited. Nonetheless, resistance evolution remains a possibility and requires experimental validation. The present study remains constrained by its reliance on computational predictions, including structure modeling, docking, and immunoinformatics analyses, which provide hypothesis-generating insights rather than direct biological evidence. Formal negative-control benchmarking—such as docking against property-matched decoys or evaluating epitope predictors using randomized or scrambled peptide sets—was not performed to compute explicit specificity metrics. Although orthogonal validation layers (MD trajectory stability assessment, MM-GBSA rescoring, and multi-filter immunoinformatics screening) were applied to reduce single-method false positives, these measures do not replace decoy-based quantification of false-discovery risk. In addition, docking scores may not directly correlate with enzymatic inhibition, MM-GBSA calculations are sensitive to force-field and solvation model assumptions, and epitope prediction algorithms are inherently limited by training dataset biases, potentially generating false positives or overestimating immunogenicity.

We therefore present Paulownin and the multi‑epitope construct as high-priority candidates for downstream confirmation; however, subsequent iterations with decoy-based enrichment testing and negative peptide benchmarking would further strengthen the specificity claims. Future validation would involve biochemical inhibition assays for Paulownin and immunogenicity testing of the vaccine construct in appropriate infection models. In addition, experimental validation will be essential to substantiate the translational potential of this integrated framework and to advance FemA-targeted interventions toward clinical applicability.

## 5. Conclusions

This study demonstrates the efficacy of in silico methods for discovering therapeutic agents targeting *Staphylococcus aureus*, specifically focusing on the essential cell wall protein FemA. We identified Paulownin, a phytocompound, as a potential high-confidence computational drug candidate with superior binding affinity and stability compared to the antibiotic Doxycycline. Additionally, we designed a multiepitope vaccine that demonstrated strong binding and stability in simulations, suggesting its potential to elicit robust immune responses. These promising results highlight Paulownin and the theoretically immunogenic construct as viable candidates for treating *S. aureus* infections, although further laboratory and animal-based studies are necessary to confirm their effectiveness. These findings provide a computational foundation for future experimental validation rather than immediate therapeutic application.

## Supporting information

S1 Table Drug-like properties of the selected phytocompounds. (DOCX)

S2 Table ADMET properties of the selected phytocompounds. (DOCX)

S3 Table The binding affinity of the phytocompounds. (DOCX)

S4 Table Predicted CTL epitopes and their physicochemical properties. (DOCX)

S5 Table Predicted HTL epitopes and their physicochemical properties. (DOCX)

S6 Table Predicted LBL epitopes and their physicochemical properties. (DOCX)
