# Supplementary material for: Computational and immunoinformatics approaches for designing phytocompound-based drugs and a multi-epitope vaccine targeting FemA, a cell wall protein of Staphylococcus aureus
Source: PLoS One. 2026 Apr 7;21(4):e0346271. doi: 10.1371/journal.pone.0346271 (PMC13056209; doi:10.1371/journal.pone.0346271)
Supplement: S1 Table — (DOCX) [file pone.0346271.s001.docx]

**S1 Table. Drug-like properties of the selected phytocompounds.**

| **SL** | **Name of the phytocompounds** | **MW (g/mol)** | **Rotatable bonds** | **H-bond acceptors** | **H-bond donors** | **Lipinski** | **Ghose** | **Veber** | **Egan** | **Muegge** | **Plants name** |
| --- | --- | --- | --- | --- | --- | --- | --- | --- | --- | --- | --- |
| 1 | 7-Desacetyl-7-benzoylazadiradione | 512.64 | 4 | 5 | 0 | 2 | 4 | 0 | 1 | 1 | *Azadirachta indica* |
| 2 | Lupane | 412.73 | 1 | 0 | 0 | 1 | 3 | 0 | 1 | 2 | *Lawsenia enermis* |
| 3 | 7-Deacetyl-gedunin | 482.57 | 3 | 7 | 0 | 0 | 1 | 0 | 0 | 0 | *Azadirachta indica* |
| 4 | Dehydrocarpaine-I | 476.69 | 0 | 6 | 1 | 0 | 2 | 0 | 0 | 1 | *Carica papaya* |
| 5 | Luteolin-3'-glucoside | 448.38 | 4 | 11 | 7 | 2 | 0 | 1 | 1 | 3 | *Lawsonia inermis* |
| 6 | Nimbinin | 466.57 | 3 | 6 | 0 | 0 | 0 | 0 | 0 | 0 | *Azadirachta indica* |
| 7 | Dehydrocarpaine-II | 474.68 | 0 | 6 | 0 | 0 | 3 | 0 | 0 | 1 | *Carica papaya* |
| 8 | Azadiradione | 450.57 | 3 | 5 | 0 | 0 | 0 | 0 | 0 | 0 | *Azadirachta indica* |
| 9 | Azadirone | 436.58 | 3 | 4 | 0 | 1 | 1 | 0 | 1 | 1 | *Azadirachta indica* |
| 10 | Carpaine | 478.71 | 0 | 6 | 2 | 0 | 2 | 0 | 0 | 1 | *Carica papaya* |
| 11 | Threonine | 119.12 | 2 | 4 | 3 | 0 | 4 | 0 | 0 | 3 | *Carica papaya* |
| 12 | 17-Epiazadiradione | 450.57 | 3 | 5 | 0 | 0 | 0 | 0 | 0 | 0 | *Azadirachta indica* |
| 13 | Apigenin-4'-glucoside | 432.38 | 4 | 10 | 6 | 1 | 0 | 1 | 1 | 2 | *Lawsenia enermis* |
| 14 | Vasicolinone | 305.37 | 2 | 2 | 0 | 0 | 0 | 0 | 0 | 0 | *Justicia adhatoda* |
| 15 | Nimbinone | 286.37 | 0 | 3 | 1 | 0 | 0 | 0 | 0 | 0 | *Azadirachta indica* |
| 16 | 6-Deacetylnimbinene | 440.53 | 4 | 6 | 1 | 0 | 0 | 0 | 0 | 0 | *Azadirachta indica* |
| 17 | Meldenin | 454.6 | 3 | 5 | 1 | 0 | 1 | 0 | 0 | 0 | *Azadirachta indica* |
| 18 | Aloeresin-D | 556.56 | 9 | 11 | 5 | 2 | 3 | 1 | 1 | 2 | *Aloe vera* |
| 19 | Delphinidin | 338.7 | 1 | 7 | 6 | 1 | 0 | 0 | 1 | 1 | *Gingiber officinale* |
| 20 | Angelicoidenol | 170.25 | 0 | 2 | 2 | 0 | 0 | 0 | 0 | 1 | *Gingiber officinale* |
| 21 | BARBALOIN | 418.39 | 3 | 9 | 7 | 1 | 1 | 1 | 1 | 2 | *Aloe vera* |
| 22 | ALOIN-B | 418.39 | 3 | 9 | 7 | 1 | 1 | 1 | 1 | 2 | *Aloe vera* |
| 23 | 7-HYDROXYALOIN | 434.39 | 3 | 10 | 8 | 1 | 1 | 1 | 1 | 2 | *Aloe vera* |
| 24 | ANISOTINE | 349.38 | 4 | 4 | 1 | 0 | 0 | 0 | 0 | 0 | *Justicia adhatoda* |
| 25 | 10-HYDROXYALOIN-B | 434.39 | 3 | 10 | 8 | 1 | 1 | 1 | 1 | 2 | *Aloe vera* |
| 26 | NIMBOCINOLIDE | 586.67 | 6 | 10 | 3 | 1 | 3 | 1 | 1 | 1 | *Azadirachta indica* |
| 27 | AZADIRACHTOL | 580.58 | 5 | 13 | 4 | 2 | 4 | 1 | 1 | 3 | *Azadirachta indica* |
| 28 | 10-HYDROXYALOIN-A | 434.39 | 3 | 10 | 8 | 1 | 1 | 1 | 1 | 2 | *Aloe vera* |
| 29 | ALOE-EMODIN | 270.24 | 1 | 5 | 3 | 0 | 0 | 0 | 0 | 0 | *Aloe vera* |
| 30 | ALOESIN | 394.37 | 4 | 9 | 5 | 0 | 1 | 1 | 1 | 1 | *Aloe vera* |
| 31 | NIMBINENE | 482.57 | 6 | 7 | 0 | 0 | 1 | 0 | 0 | 0 | *Azadirachta indica* |
| 32 | ZEAXANTHIN | 568.8 | 10 | 2 | 2 | 2 | 4 | 0 | 1 | 1 | *Carica papaya* |
| 33 | NIMBANDIOL | 456.53 | 4 | 7 | 2 | 0 | 0 | 0 | 0 | 0 | *Azadirachta indica* |
| 34 | .3-Methyl-1,1,2-TRIPHENYLCYCLOPROPANE | 284.39 | 3 | 0 | 0 | 1 | 0 | 0 | 0 | 2 | *Aloe vera* |
| 35 | 1,8-DIHYDROXYANTHRACENE | 210.23 | 0 | 2 | 2 | 0 | 0 | 0 | 0 | 0 | *Aloe vera* |
| 36 | D-GALACTURONIC-ACID | 194.14 | 1 | 7 | 5 | 0 | 2 | 0 | 0 | 2 | *Carica papaya* |
| 37 | BETA-SITOSTEROL | 414.71 | 6 | 1 | 1 | 1 | 3 | 0 | 1 | 2 | *Azadirachta indica* |
| 38 | Darunavir (ALBUMIN) | 547.66 | 13 | 8 | 3 | 1 | 3 | 2 | 1 | 0 | *Aloe vera* |
| 39 | ALOETIC-ACID | 450.23 | 5 | 13 | 3 | 1 | 0 | 1 | 1 | 2 | *Aloe vera* |
| 40 | 10-EPIZONARENE | 204.35 | 1 | 0 | 0 | 1 | 0 | 0 | 0 | 1 | *Gingiber officinale* |
| 41 | ALPHA-SELINENE | 204.35 | 1 | 0 | 0 | 1 | 0 | 0 | 0 | 2 | *Gingiber officinale* |
| 42 | LAWSARITOL | 414.71 | 6 | 1 | 1 | 1 | 3 | 0 | 1 | 2 | *Lawsenia enermis* |
| 43 | 1,3-DIHYDROXY-6,7-DIMETHOXYXANTHONE | 288.25 | 2 | 6 | 2 | 0 | 0 | 0 | 0 | 0 | *Lawsenia enermis* |
| 44 | Alpha-cadinene | 204.35 | 1 | 0 | 0 | 1 | 0 | 0 | 0 | 1 | *Gingiber officinale* |
| 45 | Camphor | 152.23 | 0 | 1 | 0 | 0 | 1 | 0 | 0 | 2 | *Gingiber officinale* |
| 46 | Ar-curcumene | 202.34 | 4 | 0 | 0 | 1 | 0 | 0 | 0 | 2 | *Gingiber officinale* |
| 47 | Mappain | 448.59 | 9 | 4 | 4 | 1 | 2 | 0 | 1 | 1 | *Carica papaya* |
| 48 | Borneol | 154.25 | 0 | 1 | 1 | 0 | 1 | 0 | 0 | 2 | *Gingiber officinale* |
| 49 | D-galactose | 180.16 | 1 | 6 | 5 | 0 | 2 | 0 | 0 | 2 | *Carica papaya* |
| 50 | Beta-Sitosterol | 414.71 | 6 | 1 | 1 | 1 | 3 | 0 | 1 | 2 | *Azadirachta indica* |
| 51 | Capsaicin | 305.41 | 10 | 3 | 2 | 0 | 0 | 0 | 0 | 0 | *Gingiber officinale* |
| 52 | Vasicinol | 204.23 | 0 | 3 | 2 | 0 | 0 | 0 | 0 | 0 | *Justicia adhatoda* |
| 53 | Beta-bisabolene | 204.35 | 4 | 0 | 0 | 1 | 0 | 0 | 0 | 2 | *Gingiber officinale* |
| 54 | Esculetin | 178.14 | 0 | 4 | 2 | 0 | 1 | 0 | 0 | 1 | *Lawsenia enermis* |
| 55 | Car-3-ene | 136.23 | 0 | 0 | 0 | 1 | 1 | 0 | 0 |  | *Gingiber officinale* |
| 56 | 1,4-Naphthaquinone | 158.15 | 0 | 2 | 0 | 0 | 2 | 0 | 0 | 1 | *Lawsenia enermis* |
| 57 | Alpha-curcumene | 202.34 | 4 | 0 | 0 | 1 | 0 | 0 | 0 | 2 | *Gingiber officinale* |
| 58 | Chymopapain-A | 204.23 | 0 | 3 | 2 | 0 | 0 | 0 | 0 | 0 | *Carica papaya* |
| 59 | Beta-carotene | 536.87 | 10 | 0 | 0 | 2 | 4 | 0 | 1 | 2 | *Ocimum sanctam* |
| 60 | Bisabolane | 210.4 | 5 | 0 | 0 | 1 | 0 | 0 | 0 | 2 | *Phyllanthus emblica* |
| 61 | Beta-bisabolol | 222.37 | 4 | 1 | 1 | 0 | 0 |  | 0 | 2 | *Gingiber officinale* |
| 62 | 1,8-Cineole | 154.25 | 0 | 1 | 0 | 0 | 1 | 0 | 0 | 2 | *Gingiber officinale* |
| 63 | 2-Methyl-2-phytyl-6-chromanol | 388.63 | 12 | 2 | 1 | 1 | 2 | 1 | 1 | 1 | *Aloe vera* |
| 64 | Beta-phellandrene | 136.23 | 1 | 0 | 0 | 0 | 1 | 0 | 0 | 2 | *Gingiber officinale* |
| 65 | Methyl chavicol benzylacetate | 280.36 | 7 | 2 | 0 | 1 | 0 | 0 | 0 | 0 | *Ocimum sanctam* |
| 66 | Alpha-zingiberene | 204.35 | 4 | 0 | 0 | 1 | 0 | 0 | 0 | 2 | *Gingiber officinale* |
| 67 | 10-Gingerol | 350.49 | 14 | 4 | 2 | 0 | 0 | 1 | 0 | 1 | *Gingiber officinale* |
| 68 | Beta-thujone | 152.23 | 1 | 1 | 0 | 0 | 1 | 0 | 0 | 2 | *Gingiber officinale* |
| 69 | Alpha-phellandrene | 136.23 | 1 | 0 | 0 | 0 | 1 | 0 | 0 | 2 | *Gingiber officinale* |
| 70 | Caffeic-acid | 180.16 | 2 | 4 | 3 | 0 | 0 | 0 | 0 | 1 | *Gingiber officinale* |
| 71 | Cis-linalool-oxide | 212.29 | 3 | 3 | 0 | 0 | 0 | 0 | 0 | 0 | *Carica papaya* |
| 72 | Alpha-terpineol | 154.25 | 1 | 1 | 1 | 0 | 1 | 0 | 0 | 2 | *Gingiber officinale* |
| 73 | Carvacrol | 150.22 | 1 | 1 | 1 | 0 | 1 | 0 | 0 | 2 | *Ocimum sanctam* |
| 74 | Carvacrol methyl ether | 164.24 | 2 | 1 | 0 | 0 | 0 | 0 | 0 | 2 | *Phyllanthus emblica* |
| 75 | 10-Dehydrogingerdione | 346.46 | 12 | 4 | 2 | 0 | 0 | 0 | 0 | 1 | *Gingiber officinale* |
| 76 | Alpha-terpinene | 136.23 | 1 | 0 | 0 | 0 | 1 | 0 | 0 | 2 | *Gingiber officinale* |
| 77 | Vasicoline | 291.39 | 2 | 1 | 0 | 0 | 0 | 0 | 0 | 0 | *Justicia adhatoda* |
| 78 | Vasicol | 206.24 | 2 | 2 | 2 | 0 | 0 | 0 | 0 | 0 | *Justicia adhatoda* |
| 79 | D-glucitol | 182.17 | 5 | 6 | 6 | 1 | 2 | 0 | 0 | 3 | *Aloe vera* |
| 80 | D-arabinose | 230.11 | 6 | 8 | 5 | 0 | 1 | 1 | 1 | 2 | *Aloe vera* |
| 81 | Myrcene | 136.23 | 4 | 0 | 0 | 0 | 1 | 0 | 0 | 2 | *Gingiber officinale* |
| 82 | Leucine | 131.17 | 3 | 3 | 2 | 0 | 2 | 0 | 0 | 1 | *Carica papaya* |
| 83 | Gingediacetate | 380.48 | 14 | 6 | 1 | 0 | 0 | 1 | 0 | 0 | *Gingiber officinale* |
| 84 | Beta-ocimene | 136.23 | 3 | 0 | 0 | 0 | 1 | 0 | 0 | 2 | *Carica papaya* |
| 85 | Oleic acid ozonide | 330.46 | 15 | 5 | 1 | 0 | 0 | 1 | 0 | 1 | *Ocimum sanctam* |
| 86 | 10-SHOGAOL | 332.48 | 13 | 3 | 1 | 0 | 0 | 1 | 0 | 1 | *Gingiber officinale* |
| 87 | 2,2,4-TRIMETHYLHEPTANE | 142.28 | 4 | 0 | 0 | 1 | 1 | 0 | 0 | 2 | *Gingiber officinale* |
| 88 | ALOINOSE | 150.13 | 0 | 5 | 4 | 0 | 3 | 0 | 0 | 2 | *Aloe vera* |
| 89 | ALPHA-CELLULOSE | 2.02 | 0 | 0 | 0 | 1 |  |  |  |  | *Aloe vera* |
| 90 | 10-GINGERDIONE | 348.48 | 13 | 4 | 2 | 0 | 0 | 1 | 0 | 1 | *Gingiber officinale* |
| 91 | NONAN-1-AL | 308.41 | 12 | 4 | 0 | 0 | 0 | 1 | 0 | 0 | *Gingiber officinale* |
| 92 | SERINE | 105.09 | 2 | 4 | 3 | 0 | 4 | 0 | 0 | 3 | *Gingiber officinale* |
| 93 | CAPRIC-ACID | 243.34 | 12 | 3 | 3 | 0 | 0 | 1 | 0 | 0 | *Gingiber officinale* |
| 94 | LYSINE | 146.19 | 5 | 4 | 3 | 0 | 3 | 0 | 0 | 2 | *Gingiber officinale* |
| 95 | GERANIOL | 154.25 | 4 | 1 | 1 | 0 | 1 | 0 | 0 | 2 | *Gingiber officinale* |
| 96 | BETAINE | 117.15 | 2 | 2 | 0 | 0 | 4 | 0 | 0 | 1 | *Justicia adhatoda* |
| 97 | GLYCINE | 75.07 | 1 | 1 | 2 | 0 | 4 | 0 |  | 3 | *Gingiber officinale* |
| 98 | N-HEPTANE | 202.33 | 6 | 2 | 0 | 0 | 0 | 0 | 0 | 0 | *Gingiber officinale* |
| 99 | LINOLEIC-ACID | 280.45 | 14 | 2 | 1 | 1 | 1 | 1 | 1 | 1 | *Ocimum sanctam* |
| 100 | PALMITIC-ACID | 256.42 | 14 | 2 | 1 | 1 | 0 | 1 | 0 | 1 | *Ocimum sanctam* |
| 101 | N-PROPANOL | 60.1 | 1 | 1 | 1 | 0 | 3 | 0 | 0 | 3 | *Gingiber officinale* |
| 102 | GALLOCATECHIN | 306.27 | 1 | 7 | 6 | 1 | 0 | 0 | 0 | 1 | *Psidium guajava* |
| 103 | L-arabinose | 150.13 | 0 | 5 | 4 | 0 | 3 | 0 | 0 | 2 | *Terminalia chebula* |
| 104 | Arginine | 174.2 | 5 | 4 | 4 | 0 | 1 | 0 | 0 | 2 | *Terminalia chebula* |
| 105 | L-Rhamnose | 164.16 | 0 | 5 | 4 | 0 | 2 | 0 | 0 | 2 | *Terminalia chebula* |
| 106 | Niacine | 123.11 | 1 | 3 | 1 | 0 | 3 | 0 | 0 | 1 | *Senna alexandrina* |
| 107 | Thiamine | 265.35 | 4 | 3 | 2 | 0 | 0 | 0 | 0 | 0 | *Senna alexandrina* |
| 108 | Methanol | 32.04 | 0 | 1 | 1 | 0 | 3 | 0 | 0 | 3 | *Elaeocarpus serratus* |
| 109 | 8-Aminocaffeine | 209.21 | 0 | 3 | 1 | 0 | 1 | 0 | 0 | 0 | *Elaeocarpus serratus* |
| 110 | Citronellyl isobutyrate | 226.36 | 8 | 2 | 0 | 0 | 0 | 0 | 0 | 0 | *Elaeocarpus serratus* |
| 111 | Niazimin A | 383.39 | 9 | 8 | 3 | 0 | 0 | 0 | 0 | 0 | *Moringa oleifera* |
| 112 | Apigenin | 270.24 | 1 | 5 | 3 | 0 | 0 | 0 | 0 | 0 | *Clerodendrum Viscosum* |
| 113 | 3,5-Di-tertbutyl-4-dihydro-benzaldehyde | 220.35 | 3 | 1 | 0 | 0 | 0 | 0 | 0 | 1 | *Aloe vera* |
| 114 | 7-HYDROXY-CHROMONE | 367.4 | 4 | 6 | 1 | 0 | 0 | 0 | 0 | 0 | *Aloe vera* |
| 115 | 2,2,8-Trimethyl-3,4-dihydro-2H-1-benzopyran-6-ol | 192.25 | 0 | 2 | 1 | 0 | 0 | 0 | 0 | 1 | *Aloe vera* |
| 116 | ALOECHRYSONE | 272.3 | 1 | 4 | 2 | 0 | 0 | 0 | 0 | 0 | *Aloe vera* |
| 117 | ALOERESIN | 394.37 | 4 | 9 | 5 | 0 | 1 | 1 | 1 | 1 | *Aloe vera* |
| 118 | ALOIN-A | 418.39 | 3 | 9 | 7 | 1 | 1 | 1 | 1 | 2 | *Aloe vera* |
| 119 | D-GALACTAN | 504.44 | 7 | 16 | 11 | 3 | 2 | 1 | 1 | 4 | *Aloe vera* |
| 120 | BETA-SITOSTEROL | 414.71 | 6 | 1 | 1 | 1 | 3 | 0 | 1 | 2 | *Ocimum sanctam* |
| 121 | SAPONINS | 1131.26 | 16 | 24 | 13 | 3 | 4 | 2 | 1 | 6 | *Ocimum sanctam* |
| 122 | XYLOSE | 150.13 | 0 | 5 | 4 | 0 | 3 | 0 | 0 | 2 | *Ocimum sanctam* |
| 123 | ARACHIDIC ACID | 312.53 | 18 | 2 | 1 | 1 | 1 | 1 | 1 | 2 | *Justicia adhatoda* |
| 124 | CEROTIC-ACID | 396.69 | 24 | 2 | 1 | 1 | 2 | 1 | 1 | 2 | *Justicia adhatoda* |
| 125 | LIGNOCERIC-ACID | 368.64 | 22 | 2 | 1 | 1 | 2 | 1 | 1 | 2 | *Justicia adhatoda* |
| 126 | OSCINE | 155.19 | 0 | 3 | 1 | 0 | 2 | 0 | 0 | 1 | *Justicia adhatoda* |
| 127 | VASICINONE | 202.21 | 0 | 3 | 1 | 0 | 0 | 0 | 0 | 0 | *Justicia adhatoda* |
| 128 | Vasicinolone | 218.21 | 0 | 4 | 2 | 0 | 0 | 0 | 0 | 0 | *Justicia adhatoda* |
| 129 | gallic acid | 170.12 | 1 | 5 | 4 | 0 | 2 | 0 | 0 | 1 | *Phyllanthus emblica* |
| 130 | Quercetin | 302.24 | 1 | 7 | 5 | 0 | 0 | 0 | 0 | 0 | *Phyllanthus emblica* |
| 131 | mucic acid | 210.14 | 5 | 8 | 6 | 1 | 2 | 1 | 1 | 3 | *Phyllanthus emblica* |
| 132 | 1,4-lactone | 86.09 | 0 | 2 | 0 | 0 | 3 | 0 | 0 | 2 | *Phyllanthus emblica* |
| 133 | 17-Hydroxyazadiradione | 466.57 | 3 | 6 | 1 | 0 | 0 | 0 | 0 | 0 | *Azadirachta indica* |
| 134 | 3-DEACETYL-SALANIN | 554.67 | 7 | 8 | 1 | 1 | 3 | 0 | 0 | 0 | *Azadirachta indica* |
| 135 | Deacetylazadirachtinol | 662.68 | 8 | 14 | 3 | 2 | 3 | 1 | 1 | 4 | *Azadirachta indica* |
| 136 | Nimbin | 540.6 | 8 | 9 | 0 | 1 | 3 | 0 | 0 | 0 | *Azadirachta indica* |
| 137 | ARACHIDIC-ACID | 312.53 | 18 | 2 | 1 | 1 | 1 | 1 | 1 | 2 | *Azadirachta indica* |
| 138 | AZADIRACHTANIN | 600.65 | 7 | 11 | 2 | 2 | 3 | 1 | 1 | 3 | *Azadirachta indica* |
| 139 | AZADIRACHTIN | 720.71 | 10 | 16 | 3 | 2 | 3 | 1 | 1 | 4 | *Azadirachta indica* |
| 140 | BEHENIC-ACID | 340.58 | 20 | 2 | 1 | 1 | 1 | 1 | 1 | 2 | *Azadirachta indica* |
| 141 | DESACETYLNIMBIN | 498.56 | 6 | 8 | 1 | 0 | 1 | 0 | 0 | 0 | *Azadirachta indica* |
| 142 | NIMBINOCINOLIDE | 572.6 | 8 | 11 | 1 | 2 | 3 | 1 | 1 | 2 | *Azadirachta indica* |
| 143 | MELIANTRIOL | 490.72 | 3 | 5 | 4 | 0 | 3 | 0 | 0 | 1 | *Azadirachta indica* |
| 144 | NIMBOLIN-A | 642.78 | 9 | 8 | 0 | 2 | 4 | 0 | 1 | 2 | *Azadirachta indica* |
| 145 | NIMBOLIN-B | 674.78 | 9 | 10 | 1 | 1 | 4 | 0 | 0 | 1 | *Azadirachta indica* |
| 146 | 1-(4-Hydroxy-3-methoxyphenyl)tetradecane-3,5-diol | 352.51 | 14 | 4 | 3 | 0 | 0 | 1 | 0 | 1 | *Gingiber offinale* |
| 147 | 6-METHYLGINGEDIOL | 310.43 | 11 | 4 | 2 | 0 | 0 | 1 | 0 | 0 | *Gingiber offinale* |
| 148 | GINGERGLYCOLIPID-A | 680.82 | 25 | 14 | 8 | 3 | 3 | 2 | 1 | 5 | *Gingiber offinale* |
| 149 | GINGERGLYCOLIPID-C | 680.82 | 25 | 14 | 8 | 3 | 3 | 2 | 1 | 5 | *Gingiber offinale* |
| 150 | EPOXY-LINALOOL | 184.23 | 4 | 3 | 1 | 0 | 0 | 0 | 0 | 1 | *Carica papaya* |
| 151 | BUTANOIC-ACID | 87.1 | 2 | 2 | 1 | 0 | 3 | 0 | 0 | 2 | *Carica papaya* |
| 152 | LYCOPENE | 536.87 | 16 | 0 | 0 | 2 | 4 | 1 | 1 | 3 | *Carica papaya* |
| 153 | N-HEPTACOSANE | 380.73 | 24 | 0 | 0 | 1 | 3 | 1 | 1 | 3 | *Carica papaya* |
| 154 | PHYTOFLUENE | 542.92 | 19 | 0 | 0 | 2 | 4 | 1 | 1 | 3 | *Carica papaya* |
| 155 | Salannin | 596.71 | 9 | 9 | 9 | 1 | 3 | 0 | 0 | 0 | *Azadirachta indica* |
| 156 | Epoxyazadiradione | 466.57 | 3 | 6 | 0 | 0 | 0 | 0 | 0 | 0 | *Azadirachta indica* |
| 157 | Nimbolide | 466.52 | 4 | 7 | 0 | 0 | 0 | 0 | 0 | 0 | *Azadirachta indica* |
| 158 | 17-Glycolyldeoxygedunin | 526.62 | 6 | 8 | 1 | 1 | 3 | 0 | 0 | 0 | *Azadirachta indica* |
| 159 | Malyngamide S | 484.07 | 14 | 5 | 2 | 0 | 3 | 1 | 0 | 0 | *Azadirachta indica* |
| 160 | 7-Acetyl-16,17-Dehydro-16-Hydroxyneotrichilenone | 466.57 | 3 | 6 | 1 | 0 | 0 | 0 | 0 | 0 | *Azadirachta indica* |
| 161 | Beta-Nimolactone | 386.48 | 2 | 5 | 0 | 0 | 0 | 0 | 0 | 0 | *Azadirachta indica* |
| 162 | Alpha-Nimolactone | 386.48 | 2 | 5 | 0 | 0 | 0 | 0 | 0 | 0 | *Azadirachta indica* |
| 163 | 17-Epi-17-Hydroxyazadiradione | 466.57 | 3 | 6 | 1 | 0 | 0 | 0 | 0 | 0 | *Azadirachta indica* |
| 164 | Nimolicinol | 482.57 | 3 | 7 | 1 | 0 | 1 | 0 | 0 | 0 | *Azadirachta indica* |
| 165 | 7-Benzoylnimbocinol | 512.64 | 4 | 5 | 0 | 2 | 4 | 0 | 1 | 1 | *Azadirachta indica* |
| 166 | Desfuranoazadiradione | 384.51 | 2 | 4 | 0 | 0 | 0 | 0 | 0 | 0 | *Azadirachta indica* |
| 167 | 15-Hydroxyazadiradione | 466.57 | 3 | 6 | 1 | 0 | 0 | 0 | 0 | 0 | *Azadirachta indica* |
| 168 | Ohchinin Acetate | 644.75 | 10 | 9 | 9 | 1 | 4 | 0 | 1 | 1 | *Azadirachta indica* |
| 169 | Vepaol | 752.76 | 11 | 17 | 3 | 2 | 3 | 2 | 1 | 4 | *Azadirachta indica* |
| 170 | Neemfruitin B | 526.7 | 4 | 6 | 1 | 1 | 3 | 0 | 0 | 1 | *Azadirachta indica* |
| 171 | Protoxylocarpin G | 528.72 | 5 | 6 | 2 | 1 | 3 | 0 | 0 | 0 | *Azadirachta indica* |
| 172 | Diacetylvilasinin | 512.63 | 5 | 7 | 1 | 1 | 3 | 0 | 0 | 0 | *Azadirachta indica* |
| 173 | Desmethyllimocin B | 470.6 | 3 | 3 | 1 | 0 | 1 | 0 | 0 | 0 | *Azadirachta indica* |
| 174 | Spicatin | 516.54 | 10 | 10 | 1 | 1 | 1 | 0 | 1 | 0 | *Azadirachta indica* |
| 175 | Neemfruitin A | 516.67 | 5 | 7 | 1 | 1 | 3 | 0 | 0 | 0 | *Azadirachta indica* |
| 176 | Ethinyl Estradiol | 296.4 | 0 | 2 | 2 | 0 | 0 | 0 | 0 | 0 | *Psidium guajava* |
| 177 | Retinol | 286.45 | 5 | 1 | 1 | 1 | 0 | 0 | 0 | 2 | *Psidium guajava* |
| 178 | Ascorbate | 176.12 | 2 | 6 | 4 | 0 | 2 | 0 | 0 | 1 | *Psidium guajava* |
| 179 | Oleanolic_Acid | 456.7 | 1 | 3 | 2 | 1 | 3 | 0 | 1 | 1 | *Psidium guajava* |
| 180 | L-Glutamate | 147.13 | 4 | 5 | 3 | 0 | 4 | 0 | 0 | 2 | *Psidium guajava* |
| 181 | Eugenol | 164.2 | 3 | 2 | 1 | 0 | 0 | 0 | 0 | 1 | *Psidium guajava* |
| 182 | Linolenic Acid | 278.43 | 13 | 2 | 1 | 1 | 1 | 1 | 1 | 1 | *Psidium guajava* |
| 183 | Arjunolic Acid | 488.7 | 2 | 5 | 4 | 0 | 3 | 0 | 0 | 1 | *Psidium guajava* |
| 184 | Genistein | 270.24 | 1 | 5 | 3 | 0 | 0 | 0 | 0 | 0 | *Allium sativum* |
| 185 | Myricetin | 318.24 | 1 | 8 | 6 | 1 | 0 | 1 | 1 | 2 | *Allium sativum* |
| 186 | Kaempferol | 286.24 | 1 | 6 | 4 | 0 | 0 | 0 | 0 | 0 | *Allium sativum* |
| 187 | Adenosine | 267.24 | 2 | 7 | 4 | 0 | 1 | 0 | 1 | 0 | *Allium sativum* |
| 188 | Rutin | 610.52 | 6 | 16 | 10 | 3 | 4 | 1 | 1 | 4 | *Allium sativum* |
| 189 | Acetylcysteine | 163.19 | 4 | 3 | 2 | 0 | 3 | 0 | 0 | 1 | *Allium sativum* |
| 190 | Coumaric Acid | 326.3 | 5 | 8 | 5 | 0 | 1 | 0 | 1 | 0 | *Allium sativum* |
| 191 | Riboflavin | 376.36 | 5 | 8 | 5 | 0 | 1 | 1 | 1 | 1 | *Allium sativum* |
| 192 | Beta Carotene | 536.87 | 10 | 0 | 0 | 2 | 4 | 0 | 1 | 2 | *Allium sativum* |
| 193 | Protocatechuic Acid | 154.12 | 1 | 4 | 3 | 0 | 3 | 0 | 0 | 1 | *Allium sativum* |
| 194 | L-Phenylalanine | 165.19 | 3 | 3 | 2 | 0 | 0 | 0 | 0 | 1 | *Allium sativum* |
| 195 | Paraben | 279.33 | 8 | 4 | 3 | 0 | 0 | 0 | 0 | 0 | *Allium sativum* |
| 196 | Ajoene | 234.4 | 8 | 1 | 0 | 0 | 0 | 0 | 0 | 0 | *Allium sativum* |
| 197 | Acetaldehyde | 44.05 | 0 | 1 | 0 | 0 | 3 | 0 | 0 | 3 | *Allium sativum* |
| 198 | Nerol | 154.25 | 4 | 1 | 1 | 0 | 1 | 0 | 0 | 2 | *Allium sativum* |
| 199 | Allicin | 162.27 | 5 | 1 | 0 | 0 | 1 | 0 | 0 | 1 | *Allium sativum* |
| 200 | Dl-Phenylalanine | 165.19 | 3 | 3 | 2 | 0 | 0 | 0 | 0 | 1 | *Allium sativum* |
| 201 | D-Glutamate | 147.13 | 4 | 5 | 3 | 0 | 4 | 0 | 0 | 2 | *Allium sativum* |
| 202 | 4-Hydroxypyrrolidin-1-Ium-2-Carboxylate | 277.17 | 3 | 9 | 2 | 0 | 1 | 0 | 0 | 1 | *Allium sativum* |
| 203 | Sinapinate | 224.21 | 4 | 5 | 2 | 0 | 0 | 0 | 0 | 0 | *Allium sativum* |
| 204 | (D)-Serine | 105.09 | 2 | 4 | 3 | 0 | 4 | 0 | 0 | 3 | *Allium sativum* |
| 205 | 2-Azaniumyl-4-Methylpentanoate | 131.17 | 3 | 2 | 1 | 0 | 3 | 0 | 0 | 1 | *Allium sativum* |
| 206 | Glycinebetaine | 117.15 | 2 | 2 | 0 | 0 | 4 | 0 | 0 | 1 | *Allium sativum* |
| 207 | Aniline | 93.13 | 0 | 0 | 1 | 0 | 3 | 0 | 0 | 2 | *Allium sativum* |
| 208 | Biotin | 244.31 | 5 | 3 | 3 | 0 | 0 | 0 | 0 | 0 | *Allium sativum* |
| 209 | Pyridoxine | 169.18 | 2 | 4 | 3 | 0 | 0 | 0 | 0 | 1 | *Allium sativum* |
| 210 | Tetraethylammonium Chloride | 165.7 | 4 | 0 | 0 | 0 | 1 | 0 | 0 | 3 | *Allium sativum* |
| 211 | N,N-Dimethyltryptamine | 188.27 | 3 | 1 | 1 | 0 | 0 | 0 | 0 | 1 | *Allium sativum* |
| 212 | Pyridoxal Phosphate | 247.14 | 4 | 7 | 3 | 0 | 0 | 0 | 0 | 0 | *Allium sativum* |
| 213 | Lariciresinol | 360.4 | 6 | 6 | 3 | 0 | 0 | 0 | 0 | 0 | *Allium sativum* |
| 214 | Matairesinol | 358.39 | 6 | 6 | 2 | 0 | 0 | 0 | 0 | 0 | *Allium sativum* |
| 215 | Coniferyl Alcohol | 180.2 | 3 | 3 | 2 | 0 | 0 | 0 | 0 | 1 | *Allium sativum* |
| 216 | Pyridoxal | 167.16 | 2 | 4 | 2 | 0 | 0 | 0 | 0 | 1 | *Allium sativum* |
| 217 | 2,3-Dihydroxybenzoic Acid | 154.12 | 1 | 4 | 3 | 0 | 3 | 0 | 0 | 1 | *Allium sativum* |
| 218 | (2R)-2-Amino-3-Methylselanylpropanoic Acid | 182.08 | 3 | 3 | 2 | 0 | 3 | 0 | 0 | 3 | *Allium sativum* |
| 219 | Elaidic Acid | 282.46 | 15 | 2 | 1 | 1 | 1 | 1 | 1 | 1 | *Allium sativum* |
| 220 | Allitridin | 178.34 | 6 | 0 | 0 | 0 | 1 | 0 | 0 | 1 | *Allium sativum* |
| 221 | D-Tyrosine | 181.19 | 3 | 4 | 3 | 0 | 0 | 0 | 0 | 2 | *Allium sativum* |
| 222 | Pyridoxamine | 168.19 | 2 | 4 | 3 | 0 | 0 | 0 | 0 | 1 | *Allium sativum* |
| 223 | Pyridoxine Hydrochloride | 205.64 | 2 | 4 | 3 | 0 | 0 | 0 | 0 | 0 | *Allium sativum* |
| 224 | Sarcosine | 89.09 | 2 | 3 | 2 | 0 | 4 | 0 | 0 | 3 | *Allium sativum* |
| 225 | Threonine | 119.12 | 2 | 4 | 3 | 0 | 4 | 0 | 0 | 3 | *Allium sativum* |
| 226 | D-Leucine | 131.17 | 3 | 3 | 2 | 0 | 2 | 0 | 0 | 1 | *Allium sativum* |
| 227 | D-Aspartate | 133.1 | 3 | 5 | 3 | 0 | 4 | 0 | 0 | 3 | *Allium sativum* |
| 228 | D-Valine | 117.15 | 2 | 3 | 2 | 0 | 3 | 0 | 0 | 2 | *Allium sativum* |
| 229 | 2-Methylbenzaldehyde | 120.15 | 1 | 1 | 0 | 0 | 3 | 0 | 0 | 2 | *Allium sativum* |
| 230 | 2-Amino-4-Methylselanylbutanoic Acid | 196.11 | 4 | 3 | 2 | 0 | 1 | 0 | 0 | 2 | *Allium sativum* |
| 231 | Ethyl Linoleate | 308.5 | 16 | 2 | 0 | 1 | 1 | 1 | 1 | 2 | *Allium sativum* |
| 232 | Thiamine Hydrochloride | 337.27 | 4 | 3 | 2 | 0 | 1 | 0 | 0 | 0 | *Allium sativum* |
| 233 | 3-Methylsulfanylpropanal | 104.17 | 3 | 1 | 0 | 0 | 3 | 0 | 0 | 2 | *Allium sativum* |
| 234 | Pentanal | 86.13 | 3 | 1 | 0 | 0 | 3 | 0 | 0 | 2 | *Allium sativum* |
| 235 | 2-Azaniumyl-3-Methylpentanoate | 131.17 | 3 | 2 | 1 | 0 | 3 | 0 | 0 | 1 | *Allium sativum* |
| 236 | Isoeugenitol | 206.19 | 0 | 4 | 2 | 0 | 0 | 0 | 0 | 0 | *Allium sativum* |
| 237 | Diallyl Sulfide | 114.21 | 4 | 0 | 0 | 0 | 3 | 0 | 0 | 2 | *Allium sativum* |
| 238 | Methylsulfinylsulfanylmethane | 110.2 | 1 | 1 | 0 | 0 | 3 | 0 | 0 | 2 | *Allium sativum* |
| 239 | Selenomethionine | 196.11 | 4 | 3 | 2 | 0 | 1 | 0 | 0 | 2 | *Allium sativum* |
| 240 | Isoleucine | 131.17 | 3 | 3 | 2 | 0 | 2 | 0 | 0 | 1 | *Allium sativum* |
| 241 | Ornithine | 132.16 | 4 | 4 | 3 | 0 | 3 | 0 | 0 | 2 | *Allium sativum* |
| 242 | D-Proline | 115.13 | 1 | 3 | 2 | 0 | 4 | 0 | 0 | 2 | *Allium sativum* |
| 243 | Racemethionine | 149.21 | 4 | 3 | 2 | 0 | 2 | 0 | 0 | 1 | *Allium sativum* |
| 244 | Alliin | 177.22 | 5 | 4 | 2 | 0 | 0 | 0 | 0 | 2 | *Allium sativum* |
| 245 | 2-Amino-3-Prop-2-Enylsulfanylpropanoic Acid | 161.22 | 5 | 3 | 2 | 0 | 0 | 0 | 0 | 2 | *Allium sativum* |
| 246 | Cis-P-Coumaric Acid | 164.16 | 2 | 3 | 2 | 0 | 0 | 0 | 0 | 1 | *Allium sativum* |
| 247 | Cis-4-Hydroxy-L-Proline | 131.13 | 1 | 4 | 3 | 0 | 4 | 0 | 0 | 2 | *Allium sativum* |
| 248 | 1-Propanethiol | 76.16 | 1 | 0 | 0 | 0 | 3 | 0 | 0 | 3 | *Allium sativum* |
| 249 | Racemic | 417.45 | 5 | 7 | 3 | 0 | 0 | 0 | 1 | 0 | *Allium sativum* |
| 250 | 1-Isothiocyanato-2-Methylpropane | 115.2 | 2 | 1 | 0 | 0 | 3 | 0 | 0 | 1 | *Allium sativum* |
| 251 | Isoflavone | 222.24 | 1 | 2 | 0 | 0 | 0 | 0 | 0 | 0 | *Allium sativum* |
| 252 | 1,2-Dimercaptocyclopentane | 134.26 | 0 | 0 | 0 | 3 | 0 | 0 | 0 | 1 | *Allium sativum* |
| 253 | 1,2-Epithiopropane | 74.14 | 0 | 0 | 0 | 0 | 3 | 0 | 0 | 3 | *Allium sativum* |
| 254 | 2,5-Dimethyltetrahydrothiophene | 189.36 | 2 | 0 | 0 | 0 | 0 | 0 | 0 | 1 | *Allium sativum* |
| 255 | 3-Methyl-2-cyclopentene-1-thione | 112.19 | 0 | 0 | 0 | 0 | 3 | 0 | 0 | 2 | *Allium sativum* |
| 256 | Sativoside B1 | 1423.49 | 22 | 35 | 22 | 3 | 4 | 2 | 1 | 7 | *Allium sativum* |
| 257 | Gitonin | 1051.17 | 11 | 23 | 13 | 3 | 4 | 2 | 1 | 5 | *Allium sativum* |
| 258 | Uttronin A | 1035.17 | 11 | 22 | 12 | 3 | 4 | 2 | 1 | 5 | *Allium sativum* |
| 259 | Sativoside R2 | 1197.31 | 14 | 27 | 15 | 3 | 4 | 2 | 1 | 5 | *Allium sativum* |
| 260 | Sativoside R1 | 1377.47 | 21 | 33 | 20 | 3 | 4 | 2 | 1 | 7 | *Allium sativum* |
| 261 | Eucalyptol | 154.25 | 0 | 1 | 0 | 0 | 1 | 0 | 0 | 2 | *Zingiber officinale* |
| 262 | Naringenin | 272.25 | 1 | 5 | 3 | 0 | 0 | 0 | 0 | 0 | *Zingiber officinale* |
| 263 | Fisetin | 286.24 | 1 | 6 | 4 | 0 | 0 | 0 | 0 | 0 | *Zingiber officinale* |
| 264 | Cianidanol | 290.27 | 1 | 6 | 5 | 0 | 0 | 0 | 0 | 0 | *Zingiber officinale* |
| 265 | Morin | 302.24 | 1 | 7 | 5 | 0 | 0 | 0 | 0 | 0 | *Zingiber officinale* |
| 266 | Limonene | 136.23 | 1 | 0 | 0 | 0 | 1 | 0 | 0 | 2 | *Zingiber officinale* |
| 267 | Vanillin | 152.15 | 2 | 3 | 1 | 0 | 2 | 0 | 0 | 1 | *Zingiber officinale* |
| 268 | Safrole | 162.19 | 2 | 2 | 0 | 0 | 0 | 0 | 0 | 1 | *Zingiber officinale* |
| 269 | Isoeugenol | 164.2 | 2 | 2 | 1 | 0 | 0 | 0 | 0 | 1 | *Zingiber officinale* |
| 270 | Cinnamic Acid | 148.16 | 2 | 2 | 1 | 0 | 2 | 0 | 0 | 1 | *Zingiber officinale* |
| 271 | 6-Paradol | 278.39 | 10 | 3 | 1 | 0 | 0 | 0 | 0 | 0 | *Zingiber officinale* |
| 272 | Dodecanoate | 199.31 | 10 | 2 | 0 | 0 | 0 | 0 | 0 | 1 | *Zingiber officinale* |
| 273 | Alpha-Bisabolol | 222.37 | 4 | 1 | 1 | 0 | 0 | 0 | 0 | 1 | *Zingiber officinale* |
| 274 | Myristic Acid | 228.37 | 12 | 2 | 1 | 0 | 0 | 1 | 0 | 1 | *Zingiber officinale* |
| 275 | Decanoic Acid | 172.26 | 8 | 2 | 1 | 0 | 0 | 0 | 0 | 1 | *Zingiber officinale* |
| 276 | Citronellol | 156.27 | 5 | 1 | 1 | 0 | 1 | 0 | 0 | 2 | *Zingiber officinale* |
| 277 | Hexanoate | 115.15 | 4 | 2 | 0 | 0 | 3 | 0 | 0 | 1 | *Zingiber officinale* |
| 278 | Methyleugenol | 178.23 | 4 | 2 | 0 | 0 | 0 | 0 | 0 | 1 | *Zingiber officinale* |
| 279 | Octanoic Acid | 144.21 | 6 | 2 | 1 | 0 | 1 | 0 | 0 | 1 | *Zingiber officinale* |
| 280 | Xanthorrizol | 218.33 | 4 | 1 | 1 | 0 | 0 | 0 | 0 | 2 | *Zingiber officinale* |
| 281 | Citronellal | 154.25 | 5 | 1 | 0 | 0 | 1 | 0 | 0 | 2 | *Zingiber officinale* |
| 282 | Stearic Acid | 284.48 | 16 | 2 | 1 | 1 | 1 | 1 | 1 | 2 | *Zingiber officinale* |
| 283 | Allo-Ocimene | 136.23 | 2 | 0 | 0 | 0 | 1 | 0 | 0 | 2 | *Zingiber officinale* |
| 284 | Perillaketone | 166.22 | 4 | 2 | 0 | 0 | 0 | 0 | 0 | 1 | *Zingiber officinale* |
| 285 | Choline | 104.17 | 2 | 1 | 1 | 0 | 2 | 0 | 0 | 1 | *Zingiber officinale* |
| 286 | Hexanal | 100.16 | 4 | 1 | 0 | 0 | 3 | 0 | 0 | 2 | *Zingiber officinale* |
| 287 | Beta-Pinene | 136.23 | 0 | 0 | 0 | 1 | 1 | 0 | 0 | 2 | *Zingiber officinale* |
| 288 | Beta-Caryophyllene | 204.35 | 0 | 0 | 0 | 1 | 0 | 0 | 0 | 1 | *Zingiber officinale* |
| 289 | Fenchlorazol-Ethyl | 403.48 | 5 | 4 | 0 | 0 | 0 | 0 | 0 | 1 | *Zingiber officinale* |
| 290 | Citropten | 206.19 | 2 | 4 | 0 | 0 | 0 | 0 | 0 | 0 | *Zingiber officinale* |
| 291 | Carene | 136.23 | 0 | 0 | 0 | 1 | 1 | 0 | 0 | 2 | *Zingiber officinale* |
| 292 | Diethyl Phthalate | 222.24 | 6 | 4 | 0 | 0 | 0 | 0 | 0 | 0 | *Zingiber officinale* |
| 293 | Ethyl Acetate | 88.11 | 2 | 2 | 0 | 0 | 3 | 0 | 0 | 2 | *Zingiber officinale* |
| 294 | Geraniin | 952.64 | 3 | 27 | 14 | 3 | 4 | 1 | 1 | 5 | *Zingiber officinale* |
| 295 | Neryl Acetate | 196.29 | 6 | 2 | 0 | 0 | 0 | 0 | 0 | 1 | *Zingiber officinale* |
| 296 | Cis-3-Hexenol | 100.16 | 3 | 1 | 1 | 0 | 3 | 0 | 0 | 2 | *Zingiber officinale* |
| 297 | Fenchyl Alcohol | 154.25 | 0 | 1 | 1 | 0 | 1 | 0 | 0 | 2 | *Zingiber officinale* |
| 298 | Oleamide | 281.48 | 15 | 1 | 1 | 1 | 0 | 1 | 0 | 1 | *Zingiber officinale* |
| 299 | Camphene | 136.23 | 0 | 0 | 0 | 1 | 1 | 0 | 0 | 2 | *Zingiber officinale* |
| 300 | 8-Gingerol | 322.44 | 12 | 4 | 2 | 0 | 0 | 1 | 0 | 0 | *Zingiber officinale* |
| 301 | Limonene Oxide | 152.23 | 1 | 1 | 0 | 0 | 1 | 0 | 0 | 2 | *Zingiber officinale* |
| 302 | P-Cymene | 134.22 | 1 | 0 | 0 | 1 | 1 | 0 | 0 | 2 | *Zingiber officinale* |
| 303 | Apocynin | 166.17 | 2 | 3 | 1 | 0 | 0 | 0 | 0 | 1 | *Zingiber officinale* |
| 304 | Zingerone | 194.23 | 4 | 3 | 1 | 0 | 0 | 0 | 0 | 1 | *Zingiber officinale* |
| 305 | Nonanoate | 157.23 | 7 | 2 | 0 | 0 | 1 | 0 | 0 | 1 | *Zingiber officinale* |
| 306 | Isoginkgetin | 566.51 | 5 | 10 | 4 | 1 | 3 | 1 | 1 | 2 | *Zingiber officinale* |
| 307 | Pinostrobin | 270.28 | 2 | 4 | 1 | 0 | 0 | 0 | 0 | 0 | *Zingiber officinale* |
| 308 | Eugenyl Acetate | 206.24 | 5 | 3 | 0 | 0 | 0 | 0 | 0 | 0 | *Zingiber officinale* |
| 309 | Spathulenol | 220.35 | 0 | 1 | 1 | 0 | 0 | 0 | 0 | 1 | *Zingiber officinale* |
| 310 | Aframodial | 318.45 | 3 | 5 | 0 | 0 | 0 | 0 | 0 | 0 | *Zingiber officinale* |
| 311 | Nonane | 128.26 | 6 | 0 | 0 | 1 | 1 | 0 | 0 | 3 | *Zingiber officinale* |
| 312 | Santamarin | 248.32 | 0 | 3 | 1 | 0 | 0 | 0 | 0 | 0 | *Zingiber officinale* |
| 313 | Nonanal | 142.24 | 7 | 1 | 0 | 0 | 1 | 0 | 0 | 2 | *Zingiber officinale* |
| 314 | Octanal | 128.21 | 6 | 1 | 0 | 0 | 1 | 0 | 0 | 2 | *Zingiber officinale* |
| 315 | Sabinene | 136.23 | 1 | 0 | 0 | 1 | 1 | 0 | 0 | 2 | *Zingiber officinale* |
| 316 | Pentadecanoic Acid | 242.4 | 13 | 2 | 1 | 0 | 0 | 1 | 0 | 1 | *Zingiber officinale* |
| 317 | Nereistoxin | 149.28 | 1 | 1 | 0 | 0 | 2 | 0 | 0 | 1 | *Zingiber officinale* |
| 318 | 4'-Methoxyglabridin | 354.4 | 2 | 5 | 2 | 0 | 0 | 0 | 0 | 0 | *Zingiber officinale* |
| 319 | 2-Methylbutan-1-Ol | 88.15 | 2 | 1 | 1 | 0 | 3 | 0 | 0 | 2 | *Zingiber officinale* |
| 320 | Pyrrolidine | 71.12 | 0 | 1 | 1 | 0 | 3 | 0 | 0 | 3 | *Zingiber officinale* |
| 321 | Palmitamide | 255.44 | 14 | 1 | 1 | 0 | 0 | 1 | 0 | 1 | *Zingiber officinale* |
| 322 | Raspberryketone | 164.2 | 3 | 2 | 1 | 0 | 0 | 0 | 0 | 1 | *Zingiber officinale* |
| 323 | Hexahydrocurcumin | 374.43 | 10 | 6 | 3 | 0 | 0 | 0 | 0 | 0 | *Zingiber officinale* |
| 324 | 6-Dehydrogingerdione | 290.35 | 9 | 4 | 1 | 0 | 0 | 0 | 0 | 0 | *Zingiber officinale* |
| 325 | Oxaldehyde | 58.04 | 1 | 2 | 0 | 0 | 4 | 0 | 0 | 2 | *Zingiber officinale* |
| 326 | 4-Ethylresorcinol | 138.16 | 1 | 2 | 2 | 0 | 1 | 0 | 0 | 1 | *Zingiber officinale* |
| 327 | Germacrene D | 204.35 | 1 | 0 | 0 | 0 | 0 | 0 | 0 | 1 | *Zingiber officinale* |
| 328 | Globulol | 222.37 | 0 | 1 | 1 | 0 | 0 | 0 | 0 | 1 | *Zingiber officinale* |
| 329 | Ethyl Propionate | 102.13 | 3 | 2 | 0 | 0 | 3 | 0 | 0 | 1 | *Zingiber officinale* |
| 330 | Verbenone | 150.22 | 0 | 1 | 0 | 0 | 1 | 0 | 0 | 2 | *Zingiber officinale* |
| 331 | Pipecolic Acid | 129.16 | 1 | 3 | 2 | 0 | 2 | 0 | 0 | 2 | *Zingiber officinale* |
| 332 | Guaiol | 222.37 | 1 | 1 | 1 | 0 | 0 | 0 | 0 | 1 | *Zingiber officinale* |
| 333 | Myrtenol | 152.23 | 1 | 1 | 1 | 0 | 1 | 0 | 0 | 2 | *Zingiber officinale* |
| 334 | Bisabolone | 220.35 | 4 | 1 | 0 | 0 | 0 | 0 | 0 | 1 | *Zingiber officinale* |
| 335 | Ethyl Hexadecanoate | 284.48 | 16 | 2 | 0 | 1 | 1 | 1 | 1 | 2 | *Zingiber officinale* |
| 336 | Olealdehyde | 266.46 | 15 | 1 | 0 | 1 | 1 | 1 | 1 | 2 | *Zingiber officinale* |
| 337 | Nerolidiol | 222.37 | 7 | 1 | 1 | 0 | 0 | 0 | 0 | 1 | *Zingiber officinale* |
| 338 | Triacontane | 422.81 | 27 | 0 | 0 | 1 | 3 | 1 | 1 | 3 | *Zingiber officinale* |
| 339 | Beta-Sesquiphellandrene | 204.35 | 4 | 0 | 0 | 0 | 0 | 0 | 0 | 2 | *Zingiber officinale* |
| 340 | Cubebin | 356.37 | 4 | 6 | 1 | 0 | 0 | 0 | 0 | 0 | *Zingiber officinale* |
| 341 | Beta-Selinene | 204.35 | 1 | 0 | 0 | 0 | 0 | 0 | 0 | 2 | *Zingiber officinale* |
| 342 | Pantothenic Acid | 219.23 | 7 | 5 | 4 | 0 | 1 | 0 | 0 | 0 | *Zingiber officinale* |
| 343 | Paravallarine | 343.5 | 1 | 3 | 1 | 0 | 0 | 0 | 0 | 0 | *Zingiber officinale* |
| 344 | Papaverine | 339.39 | 6 | 5 | 0 | 0 | 0 | 0 | 0 | 0 | *Carica papaya* |
| 345 | Daucosterol | 576.85 | 9 | 6 | 4 | 1 | 4 | 0 | 0 | 1 | *Justicia adhatoda* |
| 346 | Daidzin | 416.38 | 4 | 9 | 5 | 0 | 0 | 1 | 1 | 0 | *Justicia adhatoda* |
| 347 | Genistin | 432.38 | 4 | 10 | 6 | 1 | 0 | 1 | 1 | 2 | *Justicia adhatoda* |
| 348 | Farrerol | 300.31 | 1 | 5 | 3 | 0 | 0 | 0 | 0 | 0 | *Justicia adhatoda* |
| 349 | Vasicine | 188.23 | 0 | 2 | 1 | 0 | 0 | 0 | 0 | 1 | *Justicia adhatoda* |
| 350 | Isoquercetin | 464.38 | 4 | 12 | 8 | 2 | 1 | 1 | 1 | 3 | *Lawsonia inermis* |
| 351 | Apigetrin | 432.38 | 4 | 10 | 6 | 1 | 0 | 1 | 1 | 2 | *Lawsonia inermis* |
| 352 | Astragalin | 448.38 | 4 | 11 | 7 | 2 | 0 | 1 | 1 | 3 | *Lawsonia inermis* |
| 353 | Naphthoquinone | 158.15 | 0 | 2 | 0 | 0 | 2 | 0 | 0 | 1 | *Lawsonia inermis* |
| 354 | Diosmetin | 300.26 | 2 | 6 | 3 | 0 | 0 | 0 | 0 | 0 | *Lawsonia inermis* |
| 355 | Isoimperatorin | 270.28 | 3 | 4 | 0 | 0 | 0 | 0 | 0 | 0 | *Lawsonia inermis* |
| 356 | Spiraeoside | 464.38 | 4 | 12 | 8 | 2 | 1 | 1 | 1 | 3 | *Lawsonia inermis* |
| 357 | Elemicin | 208.25 | 5 | 3 | 0 | 0 | 0 | 0 | 0 | 0 | *Lawsonia inermis* |
| 358 | Tilianine | 446.4 | 5 | 10 | 5 | 0 | 0 | 1 | 1 | 1 | *Lawsonia inermis* |
| 359 | Daphnorin | 514.43 | 6 | 12 | 4 | 2 | 1 | 1 | 1 | 2 | *Lawsonia inermis* |
| 360 | Apiol | 222.24 | 4 | 4 | 0 | 0 | 0 | 0 | 0 | 0 | *Lawsonia inermis* |
| 361 | Apiin | 564.49 | 7 | 14 | 8 | 3 | 3 | 1 | 1 | 3 | *Lawsonia inermis* |
| 362 | Icariside F2 | 402.39 | 7 | 10 | 6 | 1 | 1 | 1 | 1 | 3 | *Lawsonia inermis* |
| 363 | Acacetin-7-O-Beta-D-Galactopyranoside | 446.4 | 5 | 10 | 5 | 0 | 0 | 1 | 1 | 1 | *Lawsonia inermis* |
| 364 | Kinidilin | 300.31 | 4 | 5 | 0 | 0 | 0 | 0 | 0 | 0 | *Lawsonia inermis* |
| 365 | Daphneside | 502.42 | 6 | 14 | 8 | 3 | 2 | 1 | 1 | 4 | *Lawsonia inermis* |
| 366 | Hydrocortisone | 362.43 | 2 | 5 | 3 | 0 | 0 | 0 | 0 | 0 | *Aloe vera* |
| 367 | Catechol | 110.11 | 0 | 2 | 2 | 0 | 3 | 0 | 0 | 1 | *Aloe vera* |
| 368 | Rhein | 284.22 | 1 | 6 | 3 | 0 | 0 | 0 | 0 | 0 | *Aloe vera* |
| 369 | Galangin | 270.24 | 1 | 5 | 3 | 0 | 0 | 0 | 0 | 0 | *Aloe vera* |
| 370 | Acrolein | 56.06 | 1 | 1 | 0 | 0 | 3 | 0 | 0 | 3 | *Aloe vera* |
| 371 | Phlorizin | 436.41 | 7 | 10 | 7 | 1 | 0 | 1 | 1 | 2 | *Aloe vera* |
| 372 | Vanillic Acid | 168.15 | 2 | 4 | 2 | 0 | 0 | 0 | 0 | 1 | *Aloe vera* |
| 373 | Stigmasterol | 412.69 | 5 | 1 | 1 | 1 | 3 | 0 | 1 | 2 | *Aloe vera* |
| 374 | Glutathione | 307.32 | 11 | 7 | 5 | 0 | 1 | 2 | 1 | 2 | *Aloe vera* |
| 375 | Azelaic Acid | 188.22 | 8 | 4 | 2 | 0 | 0 | 0 | 0 | 1 | *Aloe vera* |
| 376 | Homovanillic Acid | 182.17 | 3 | 4 | 2 | 0 | 0 | 0 | 0 | 1 | *Aloe vera* |
| 377 | Hypoxanthine | 136.11 | 0 | 3 | 2 | 0 | 3 | 0 | 0 | 1 | *Aloe vera* |
| 378 | 4-Methoxycinnamic Acid | 178.18 | 3 | 3 | 1 | 0 | 0 | 0 | 0 | 1 | *Aloe vera* |
| 379 | Furfural | 96.08 | 1 | 2 | 0 | 0 | 3 | 0 | 0 | 1 | *Aloe vera* |
| 380 | Butanedione | 86.09 | 1 | 2 | 0 | 0 | 3 | 0 | 0 | 2 | *Aloe vera* |
| 381 | 4-Methylbenzoic Acid | 136.15 | 1 | 2 | 1 | 0 | 3 | 0 | 0 | 1 | *Aloe vera* |
| 382 | Pyroglutamic Acid | 129.11 | 1 | 3 | 2 | 0 | 4 | 0 | 0 | 1 | *Aloe vera* |
| 383 | Methanesulfonylmethane | 94.13 | 0 | 2 | 0 | 0 | 3 | 0 | 0 | 2 | *Aloe vera* |
| 384 | Heptanal | 114.19 | 5 | 1 | 0 | 0 | 2 | 0 | 0 | 2 | *Aloe vera* |
| 385 | Butanone | 72.11 | 1 | 1 | 0 | 0 | 3 | 0 | 0 | 3 | *Aloe vera* |
| 386 | Malic Acid | 134.09 | 3 | 5 | 3 | 0 | 4 | 0 | 0 | 2 | *Aloe vera* |
| 387 | Campesteryl Ferulate | 576.85 | 10 | 4 | 1 | 2 | 4 | 0 | 1 | 1 | *Aloe vera* |
| 388 | Tartrate | 150.09 | 3 | 6 | 4 | 0 | 4 | 0 | 0 | 2 | *Aloe vera* |
| 389 | Phenylpyruvate | 164.16 | 3 | 3 | 1 | 0 | 0 | 0 | 0 | 1 | *Aloe vera* |
| 390 | Tartaric Acid | 150.09 | 3 | 6 | 4 | 0 | 4 | 0 | 0 | 2 | *Aloe vera* |
| 391 | Nataloe-Emodin | 270.24 | 0 | 5 | 3 | 0 | 0 | 0 | 0 | 0 | *Aloe vera* |
| 392 | 2-Decanone | 156.27 | 7 | 1 | 0 | 0 | 1 | 0 | 0 | 2 | *Aloe vera* |
| 393 | Isobutyraldehyde | 72.11 | 1 | 1 | 0 | 0 | 3 | 0 | 0 | 3 | *Aloe vera* |
| 394 | 2-Methylbutanal | 86.13 | 2 | 1 | 0 | 0 | 3 | 0 | 0 | 2 | *Aloe vera* |
| 395 | Elcosanoic Acid | 312.53 | 18 | 2 | 1 | 1 | 1 | 1 | 1 | 2 | *Aloe vera* |
| 396 | 3-Methylbutanoic Acid | 102.13 | 2 | 2 | 1 | 0 | 3 | 0 | 0 | 1 | *Aloe vera* |
| 397 | Pidolic Acid | 129.11 | 1 | 3 | 2 | 0 | 4 | 0 | 0 | 1 | *Aloe vera* |
| 398 | Methyl Isobutyl Ketone | 100.16 | 2 | 1 | 0 | 0 | 3 | 0 | 0 | 2 | *Aloe vera* |
| 399 | Madagascin | 338.35 | 3 | 5 | 2 | 0 | 0 | 0 | 0 | 0 | *Aloe vera* |
| 400 | Glycolic Acid | 76.05 | 1 | 3 | 2 | 0 | 4 | 0 | 0 | 2 | *Aloe vera* |
| 401 | Furan-2-Carboxylic Acid | 112.08 | 1 | 3 | 1 | 0 | 3 | 0 | 0 | 1 | *Aloe vera* |
| 402 | 2-Hydroxybutyric Acid | 104.1 | 2 | 3 | 2 | 0 | 3 | 0 | 0 | 2 | *Aloe vera* |
| 403 | 2-Methylfuran | 82.1 | 0 | 1 | 0 | 0 | 3 | 0 | 0 | 2 | *Aloe vera* |
| 404 | 2-Dodecyl-3-Methylbutanedioic Acid | 300.43 | 14 | 4 | 2 | 0 | 0 | 1 | 0 | 1 | *Aloe vera* |
| 405 | Oxane-2,3,4,5-Tetrol | 150.13 | 0 | 5 | 4 | 0 | 3 | 0 | 0 | 2 | *Aloe vera* |
| 406 | Aloinoside B | 564.54 | 5 | 13 | 9 | 3 | 4 | 1 | 1 | 3 | *Aloe vera* |
| 407 | Hispiduloside | 462.4 | 5 | 11 | 6 | 2 | 0 | 1 | 1 | 3 | *Aloe vera* |
| 408 | Arabitol(D) | 152.15 | 4 | 5 | 5 | 0 | 3 | 0 | 0 | 2 | *Aloe vera* |
| 409 | Crotonaldehyde | 70.09 | 1 | 1 | 0 | 0 | 3 | 0 | 0 | 3 | *Aloe vera* |
| 410 | Propylformate | 87.1 | 2 | 2 | 0 | 0 | 4 | 0 | 0 | 2 | *Aloe vera* |
| 411 | Ellagic Acid | 302.19 | 0 | 8 | 4 | 0 | 0 | 1 | 1 | 0 | *Terminalia chebula* |
| 412 | Pyrogallol | 126.11 | 0 | 3 | 3 | 0 | 3 | 0 | 0 | 1 | *Terminalia chebula* |
| 413 | Erucic Acid | 338.57 | 19 | 2 | 1 | 1 | 1 | 1 | 1 | 2 | *Terminalia chebula* |
| 414 | Nicotifloroside | 594.52 | 6 | 15 | 9 | 3 | 4 | 1 | 1 | 3 | *Terminalia chebula* |
| 415 | Corilagin | 634.45 | 3 | 18 | 11 | 3 | 2 | 1 | 1 | 4 | *Terminalia chebula* |
| 416 | Phthalic Acid | 166.13 | 2 | 4 | 2 | 0 | 1 | 0 | 0 | 1 | *Terminalia chebula* |
| 417 | Ethylgallate | 198.17 | 3 | 5 | 3 | 0 | 0 | 0 | 0 | 1 | *Terminalia chebula* |
| 418 | Punicalagin | 1084.72 | 0 | 30 | 17 | 3 | 3 | 1 | 1 | 5 | *Terminalia chebula* |
| 419 | Chebulinic Acid | 956.68 | 12 | 27 | 13 | 3 | 3 | 2 | 1 | 4 | *Terminalia chebula* |
| 420 | Punicalin | 782.53 | 0 | 22 | 13 | 3 | 3 | 1 | 1 | 5 | *Terminalia chebula* |
| 421 | Ethyl Cinnamate | 176.21 | 4 | 2 | 0 | 0 | 0 | 0 | 0 | 1 | *Terminalia chebula* |
| 422 | N-Octacosanoic Acid | 424.74 | 26 | 2 | 1 | 1 | 3 | 1 | 1 | 2 | *Terminalia chebula* |
| 423 | Phenylacetaldehyde | 120.15 | 2 | 1 | 0 | 0 | 2 | 0 | 0 | 2 | *Terminalia chebula* |
| 424 | Squalene | 410.72 | 15 | 0 | 0 | 1 | 3 | 1 | 1 | 2 | *Terminalia chebula* |
| 425 | Methyl Nonyl Ketone | 170.29 | 8 | 1 | 0 | 0 | 0 | 0 | 0 | 2 | *Terminalia chebula* |
| 426 | Cyclohexane | 84.16 | 0 | 0 | 0 | 0 | 3 | 0 | 0 | 2 | *Terminalia chebula* |
| 427 | Hexadecane | 226.44 | 13 | 0 | 0 | 1 | 1 | 1 | 1 | 2 | *Terminalia chebula* |
| 428 | Nigaichigoside F1 | 66.84 | 5 | 11 | 8 | 3 | 3 | 1 | 1 | 4 | *Terminalia chebula* |
| 429 | Oblonganoside | 764.64 | 7 | 13 | 8 | 3 | 3 | 1 | 1 | 4 | *Terminalia chebula* |
| 430 | Indene | 116.16 | 0 | 0 | 0 | 0 | 3 | 0 | 0 | 2 | *Terminalia chebula* |
| 431 | Arjungenin | 504.7 | 2 | 6 | 5 | 1 | 3 | 0 | 0 | 0 | *Terminalia chebula* |
| 432 | Casuarinin | 936.65 | 4 | 26 | 16 | 3 | 3 | 1 | 1 | 5 | *Terminalia chebula* |
| 433 | 1,16-Hexadecanediol | 258.44 | 15 | 2 | 2 | 0 | 0 | 1 | 0 | 1 | *Terminalia chebula* |
| 434 | Oblonganoside | 764.64 | 7 | 13 | 8 | 3 | 3 | 1 | 1 | 4 | *Terminalia chebula* |
| 435 | Cheilanthifoline | 325.36 | 1 | 5 | 1 | 0 | 0 | 0 | 0 | 0 | *Terminalia chebula* |
| 436 | Heptadecane | 240.47 | 14 | 0 | 0 | 1 | 1 | 1 | 1 | 2 | *Terminalia chebula* |
| 437 | Ethyl Oleate | 310.51 | 17 | 2 | 0 | 1 | 1 | 1 | 1 | 2 | *Terminalia chebula* |
| 438 | Arjunglucoside I | 666.84 | 5 | 11 | 8 | 3 | 3 | 1 | 1 | 4 | *Terminalia chebula* |
| 439 | Pedunculoside | 650.84 | 5 | 10 | 7 | 2 | 3 | 1 | 1 | 3 | *Terminalia chebula* |
| 440 | Chebuloside Ii | 666.84 | 5 | 11 | 8 | 3 | 3 | 1 | 1 | 4 | *Terminalia chebula* |
| 441 | Cyclododecane | 168.32 | 0 | 0 | 0 | 1 | 0 | 0 | 0 | 2 | *Terminalia chebula* |
| 442 | Terflavin A | 1086.73 | 8 | 30 | 17 | 3 | 3 | 1 | 1 | 5 | *Terminalia chebula* |
| 443 | Rhein | 284.22 | 1 | 6 | 3 | 0 | 0 | 0 | 0 | 0 | *Senna alexandrina* |
| 444 | Methyl Salicylate | 152.15 | 2 | 3 | 1 | 0 | 3 | 0 | 0 | 1 | *Senna alexandrina* |
| 445 | Physcion | 284.26 | 1 | 5 | 2 | 0 | 0 | 0 | 0 | 0 | *Senna alexandrina* |
| 446 | Chrysophanol | 254.24 | 0 | 4 | 2 | 0 | 0 | 0 | 0 | 0 | *Senna alexandrina* |
| 447 | Isorhamnetin | 316.26 | 2 | 7 | 4 | 0 | 0 | 0 | 0 | 0 | *Senna alexandrina* |
| 448 | Menthol | 156.27 | 1 | 1 | 1 | 0 | 1 | 0 | 0 | 2 | *Senna alexandrina* |
| 449 | Pulegone | 152.23 | 0 | 1 | 0 | 0 | 1 | 0 | 0 | 2 | *Senna alexandrina* |
| 450 | Carvone | 150.22 | 1 | 1 | 0 | 0 | 1 | 0 | 0 | 2 | *Senna alexandrina* |
| 451 | Anethole | 148.2 | 2 | 1 | 0 | 0 | 1 | 0 | 0 | 2 | *Senna alexandrina* |
| 452 | Levomenthol | 156.27 | 1 | 1 | 1 | 0 | 1 | 0 | 0 | 2 | *Senna alexandrina* |
| 453 | Sennoside B | 862.74 | 9 | 20 | 12 | 3 | 4 | 1 | 1 | 5 | *Senna alexandrina* |
| 454 | Fenchone | 152.23 | 0 | 1 | 0 | 0 | 1 | 0 | 0 | 2 | *Senna alexandrina* |
| 455 | Sennoside A | 862.74 | 9 | 20 | 12 | 3 | 4 | 1 | 1 | 5 | *Senna alexandrina* |
| 456 | Undecane | 156.31 | 8 | 0 | 0 | 1 | 1 | 0 | 0 | 3 | *Senna alexandrina* |
| 457 | Tridecane | 184.36 | 10 | 0 | 0 | 1 | 0 | 0 | 0 | 3 | *Senna alexandrina* |
| 458 | Pentadecane | 212.41 | 12 | 0 | 0 | 1 | 1 | 1 | 1 | 2 | *Senna alexandrina* |
| 459 | Cyclocitral | 152.23 | 1 | 1 | 0 | 0 | 1 | 0 | 0 | 2 | *Senna alexandrina* |
| 460 | Longiforene | 204.35 | 0 | 0 | 0 | 1 | 0 | 0 | 0 | 2 | *Senna alexandrina* |
| 461 | Racementhol | 156.27 | 1 | 1 | 1 | 0 | 1 | 0 | 0 | 2 | *Senna alexandrina* |
| 462 | Ayanin | 344.32 | 4 | 7 | 2 | 0 | 0 | 0 | 0 | 0 | *Phyllanthus emblica* |
| 463 | Furosin | 650.45 | 4 | 19 | 10 | 3 | 3 | 1 | 1 | 5 | *Phyllanthus emblica* |
| 464 | 7-Hydroxy-2-(4-Hydroxyphenyl)Chromen-4-One | 256.25 | 1 | 4 | 2 | 0 | 0 | 0 | 0 | 0 | *Phyllanthus emblica* |
| 465 | Undecanol | 172.31 | 9 | 1 | 1 | 0 | 0 | 0 | 0 | 2 | *Phyllanthus emblica* |
| 466 | Putrescine | 88.15 | 3 | 2 | 2 | 0 | 3 | 0 | 0 | 2 | *Phyllanthus emblica* |
| 467 | Chebulagic Acid | 954.66 | 5 | 27 | 13 | 3 | 3 | 1 | 1 | 5 | *Phyllanthus emblica* |
| 468 | Entagenic Acid | 488.7 | 1 | 5 | 4 | 0 | 3 | 0 | 0 | 1 | *Phyllanthus emblica* |
| 469 | Beta-Glucogallin | 332.26 | 4 | 10 | 7 | 1 | 1 | 1 | 1 | 2 | *Phyllanthus emblica* |
| 470 | Punicafolin | 938.66 | 9 | 26 | 15 | 3 | 3 | 1 | 1 | 4 | *Phyllanthus emblica* |
| 471 | Phaseoloidin | 330.29 | 5 | 9 | 6 | 1 | 1 | 1 | 1 | 2 | *Phyllanthus emblica* |
| 472 | Entadamide | 161.22 | 5 | 2 | 2 | 0 | 0 | 0 | 0 | 1 | *Phyllanthus emblica* |
| 473 | Harpagide | 364.35 | 3 | 10 | 7 | 1 | 1 | 1 | 1 | 3 | *Phyllanthus emblica* |
| 474 | 2-Pentadecanone | 226.4 | 12 | 1 | 0 | 0 | 0 | 1 | 0 | 2 | *Phyllanthus emblica* |
| 475 | Elaeocarpusin | 788.57 | 13 | 22 | 13 | 3 | 3 | 2 | 1 | 4 | *Phyllanthus emblica* |
| 476 | phyllaemblicin | 494.94 | 6 | 13 | 8 | 2 | 2 | 1 | 1 | 4 | *Phyllanthus emblica* |
| 477 | Disogenin | 414.64 | 0 | 3 | 1 | 1 | 2 | 0 | 0 | 1 | *Clerodendrum infortunatum* |
| 478 | 3-Acetylbetulinic Acid | 498.78 | 4 | 4 | 1 | 1 | 4 | 0 | 1 | 1 | *Clerodendrum infortunatum* |
| 479 | Physalin F | 526.53 | 0 | 10 | 1 | 1 | 1 | 0 | 1 | 1 | *Clerodendrum infortunatum* |
| 480 | Solamargine | 868.06 | 7 | 16 | 9 | 3 | 3 | 1 | 1 | 5 | *Clerodendrum infortunatum* |
| 481 | Degalactotigonin | 1035.17 | 11 | 22 | 12 | 3 | 4 | 1 | 2 | 5 | *Clerodendrum infortunatum* |
| 482 | Solasonine | 884.06 | 8 | 17 | 10 | 3 | 4 | 1 | 1 | 5 | *Clerodendrum infortunatum* |
| 483 | Tigogenin | 416.64 | 0 | 3 | 1 | 1 | 2 | 0 | 0 | 1 | *Clerodendrum infortunatum* |
| 484 | Physalin G | 526.53 | 0 | 10 | 2 | 1 | 1 | 1 | 1 | 1 | *Clerodendrum infortunatum* |
| 485 | Khasianine | 721.92 | 5 | 12 | 7 | 3 | 3 | 1 | 1 | 5 | *Clerodendrum infortunatum* |
| 486 | Ferulic Acid | 194.18 | 3 | 4 | 2 | 0 | 0 | 0 | 0 | 1 | *Ocimum tenuiflorum* |
| 487 | Androstenedione | 286.41 | 0 | 2 | 0 | 0 | 0 | 0 | 0 | 0 | *Ocimum tenuiflorum* |
| 488 | Dehydroabietic Acid | 300.44 | 2 | 2 | 1 | 1 | 0 | 0 | 0 | 0 | *Ocimum tenuiflorum* |
| 489 | Afzelin | 432.38 | 3 | 10 | 6 | 1 | 0 | 1 | 1 | 2 | *Ocimum tenuiflorum* |
| 490 | Estragole | 148.2 | 3 | 1 | 0 | 0 | 1 | 0 | 0 | 2 | *Ocimum tenuiflorum* |
| 491 | Enniatin B | 639.82 | 6 | 9 | 0 | 2 | 3 | 0 | 1 | 2 | *Ocimum tenuiflorum* |
| 492 | Procyanidin B2 | 578.52 | 3 | 12 | 10 | 3 | 2 | 1 | 1 | 3 | *Ocimum tenuiflorum* |
| 493 | Pinosylvin | 212.24 | 2 | 2 | 2 | 0 | 0 | 0 | 0 | 0 | *Ocimum tenuiflorum* |
| 494 | Isolariciresinol | 360.4 | 5 | 6 | 4 | 0 | 0 | 0 | 0 | 0 | *Ocimum tenuiflorum* |
| 495 | Procyanidin | 594.52 | 4 | 13 | 10 | 3 | 2 | 1 | 1 | 3 | *Ocimum tenuiflorum* |
| 496 | Methylindole-3-Carboxylate | 175.18 | 2 | 2 | 1 | 0 | 0 | 0 | 0 | 1 | *Ocimum tenuiflorum* |
| 497 | Galactitol | 182.17 | 5 | 6 | 6 | 1 | 2 | 0 | 0 | 3 | *Ocimum tenuiflorum* |
| 498 | Quinic Acid | 192.17 | 1 | 6 | 5 | 0 | 1 | 0 | 0 | 2 | *Ocimum tenuiflorum* |
| 499 | 12-Hydroxydehydroabietic Acid | 316.43 | 2 | 3 | 2 | 0 | 0 | 0 | 0 | 0 | *Ocimum tenuiflorum* |
| 500 | Shikimic Acid | 174.15 | 1 | 5 | 4 | 0 | 2 | 0 | 0 | 1 | *Ocimum tenuiflorum* |
| 501 | Pinifolic Acid | 336.47 | 6 | 4 | 2 | 0 | 0 | 0 | 0 | 0 | *Ocimum tenuiflorum* |
| 502 | Pentane-2,4-Dione | 100.12 | 2 | 2 | 0 | 0 | 3 | 0 | 0 | 1 | *Ocimum tenuiflorum* |
| 503 | Enniatin B1 | 635.85 | 7 | 9 | 0 | 2 | 3 | 0 | 1 | 2 | *Ocimum tenuiflorum* |
| 504 | Pinitol | 194.18 | 1 | 6 | 5 | 0 | 1 | 0 | 0 | 2 | *Ocimum tenuiflorum* |
| 505 | Dihydroconiferin | 344.36 | 7 | 8 | 5 | 0 | 1 | 0 | 0 | 0 | *Ocimum tenuiflorum* |
| 506 | Gob C | 390.38 | 5 | 8 | 6 | 1 | 0 | 0 | 1 | 1 | *Ocimum tenuiflorum* |
| 507 | Beta-Hydroxypropiovanillone | 196.2 | 4 | 4 | 2 | 0 | 0 | 0 | 0 | 1 | *Ocimum tenuiflorum* |
| 508 | Terpinolene | 136.23 | 0 | 0 | 0 | 0 | 1 | 0 | 0 | 2 | *Ocimum tenuiflorum* |
| 509 | Stigmastanol | 416.72 | 6 | 1 | 1 | 1 | 3 | 0 | 1 | 2 | *Ocimum tenuiflorum* |
| 510 | Grifolin | 328.49 | 8 | 2 | 2 | 1 | 1 | 0 | 1 | 1 | *Ocimum tenuiflorum* |
| 511 | Isoabienol | 290.48 | 4 | 1 | 1 | 1 | 0 | 0 | 0 | 2 | *Ocimum tenuiflorum* |
| 512 | Enniatin J1 | 611.67 | 5 | 9 | 0 | 2 | 3 | 0 | 1 | 2 | *Ocimum tenuiflorum* |
| 513 | Enniatin K1 | 625.79 | 6 | 9 | 0 | 2 | 3 | 0 | 1 | 2 | *Ocimum tenuiflorum* |
| 514 | Citrusin D | 342.34 | 6 | 8 | 5 | 0 | 1 | 0 | 0 | 0 | *Ocimum tenuiflorum* |
| 515 | Erythro-Guaiacylglycerol | 214.22 | 4 | 5 | 4 | 0 | 1 | 0 | 0 | 0 | *Ocimum tenuiflorum* |
| 516 | Abietic Acid | 302.45 | 2 | 2 | 1 | 1 | 0 | 0 | 0 | 0 | *Elaeocarpus serratus* |
| 517 | Pomiferin A | 286.45 | 2 | 1 | 1 | 1 | 0 | 0 | 0 | 2 | *Elaeocarpus serratus* |
| 518 | 7-Dehyroabietanone | 284.22 | 1 | 1 | 0 | 1 | 0 | 0 | 0 | 2 | *Elaeocarpus serratus* |
| 519 | Abietadien-18-Ol | 288.47 | 2 | 1 | 1 | 1 | 0 | 0 | 0 | 1 | *Elaeocarpus serratus* |
| 520 | 7-Oxodehydroabietinol | 300.44 | 2 | 2 | 1 | 0 | 0 | 0 | 0 | 0 | *Elaeocarpus serratus* |
| 521 | Syringetin | 346.29 | 3 | 8 | 4 | 0 | 0 | 0 | 0 | 0 | *Elaeocarpus serratus* |
| 522 | Abietadien-18-Al | 286.45 | 2 | 1 | 0 | 1 | 0 | 0 | 0 | 1 | *Elaeocarpus serratus* |
| 523 | Dehydroabietinal | 284.44 | 2 | 1 | 0 | 1 | 0 | 0 | 0 | 1 | *Elaeocarpus serratus* |
| 524 | 7Beta,18-Dihydroxydehydroabietanol | 302.45 | 2 | 2 | 2 | 0 | 0 | 0 | 0 | 0 | *Elaeocarpus serratus* |
| 525 | Ursolic Acid | 456.7 | 1 | 3 | 2 | 1 | 3 | 0 | 1 | 1 | *Elaeocarpus serratus* |
| 526 | Methylparaben | 152.15 | 2 | 3 | 1 | 0 | 3 | 0 | 0 | 1 | *Moringa oleifera* |
| 527 | Acetylcholine | 146.21 | 4 | 2 | 0 | 0 | 2 | 0 | 0 | 2 | *Moringa oleifera* |
| 528 | Aminobenzoic Acid | 137.14 | 1 | 2 | 2 | 0 | 3 | 0 | 0 | 1 | *Moringa oleifera* |
| 529 | 4-Hydroxybenzaldehyde | 122.12 | 1 | 2 | 1 | 0 | 3 | 0 | 0 | 1 | *Moringa oleifera* |
| 530 | Xanthoxyletin | 258.27 | 1 | 4 | 0 | 0 | 0 | 0 | 0 | 0 | *Moringa oleifera* |
| 531 | Hesperidin | 610.56 | 7 | 15 | 8 | 3 | 4 | 1 | 1 | 4 | *Moringa oleifera* |
| 532 | Gamma-Terpinene | 136.23 | 1 | 0 | 0 | 0 | 1 | 0 | 0 | 2 | *Moringa oleifera* |
| 533 | Sesamin | 354.35 | 2 | 6 | 0 | 0 | 0 | 0 | 0 | 0 | *Moringa oleifera* |
| 534 | Lasiodiplodin | 292.37 | 1 | 4 | 1 | 0 | 0 | 0 | 0 | 0 | *Moringa oleifera* |
| 535 | Sesalin | 228.24 | 0 | 3 | 0 | 0 | 0 | 0 | 0 | 0 | *Moringa oleifera* |
| 536 | Dentatin | 326.39 | 3 | 4 | 0 | 0 | 0 | 0 | 0 | 0 | *Moringa oleifera* |
| 537 | Nordentatin | 312.36 | 2 | 4 | 1 | 0 | 0 | 0 | 0 | 0 | *Moringa oleifera* |
| 538 | Narirutin | 580.53 | 6 | 14 | 8 | 3 | 4 | 1 | 1 | 3 | *Moringa oleifera* |
| 539 | Clausarin | 380.48 | 4 | 4 | 1 | 0 | 0 | 0 | 0 | 1 | *Moringa oleifera* |
| 540 | Sesamolin | 370.35 | 3 | 7 | 0 | 0 | 0 | 0 | 0 | 0 | *Moringa oleifera* |
| 541 | Niazinin A | 343.4 | 6 | 6 | 4 | 0 | 0 | 0 | 1 | 0 | *Moringa oleifera* |
| 542 | Niazirin | 279.29 | 3 | 6 | 3 | 0 | 0 | 0 | 0 | 0 | *Moringa oleifera* |
| 543 | Niazicin A | 385.43 | 8 | 7 | 3 | 0 | 0 | 0 | 1 | 0 | *Moringa oleifera* |
| 544 | 2-Phenylacetonitrile | 117.15 | 1 | 1 | 0 | 0 | 3 | 0 | 0 | 2 | *Moringa oleifera* |
| 545 | 1,3-Dibenzylurea | 240.3 | 6 | 1 | 2 | 0 | 0 | 0 | 0 | 0 | *Moringa oleifera* |
| 546 | Arjunic Acid | 488.7 | 1 | 5 | 4 | 0 | 3 | 0 | 0 | 1 | *Terminalia arjuna* |
| 547 | Luteolin | 286.24 | 1 | 6 | 4 | 0 | 0 | 0 | 0 | 0 | *Allium cepa* |
| 548 | Dopamine | 153.18 | 2 | 3 | 3 | 0 | 1 | 0 | 0 | 1 | *Allium cepa* |
| 549 | Levodopa | 197.19 | 3 | 5 | 4 | 0 | 0 | 0 | 0 | 2 | *Allium cepa* |
| 550 | Benzoic Acid | 122.11 | 1 | 2 | 1 | 0 | 3 | 0 | 0 | 1 | *Allium cepa* |
| 551 | Benzyl Isothiocyanate | 149.21 | 2 | 1 | 0 | 0 | 2 | 0 | 0 | 1 | *Allium cepa* |
| 552 | Taurine | 125.15 | 2 | 4 | 2 | 0 | 3 | 0 | 0 | 3 | *Allium cepa* |
| 553 | Diphenylamine | 169.22 | 2 | 0 | 1 | 0 | 0 | 0 | 0 | 2 | *Allium cepa* |
| 554 | Diallyldisulfide | 146.27 | 5 | 0 | 0 | 0 | 2 | 0 | 0 | 1 | *Allium cepa* |
| 555 | O-Coumaric Acid | 164.16 | 2 | 3 | 2 | 0 | 0 | 0 | 0 | 1 | *Allium cepa* |
| 556 | Diffractaic Acid | 374.38 | 6 | 7 | 2 | 0 | 0 | 0 | 0 | 0 | *Allium cepa* |
| 557 | Creatinine | 113.12 | 0 | 2 | 1 | 0 | 4 | 0 | 0 | 2 | *Allium cepa* |
| 558 | Phloroglucinol | 126.11 | 0 | 3 | 3 | 0 | 3 | 0 | 0 | 1 | *Allium cepa* |
| 559 | Trigonelline | 137.14 | 1 | 2 | 0 | 0 | 4 | 0 | 0 | 1 | *Allium cepa* |
| 560 | Propionaldehyde | 58.08 | 1 | 1 | 0 | 0 | 3 | 0 | 0 | 3 | *Allium cepa* |
| 561 | Delphinidin 3-Glucoside | 465.38 | 4 | 12 | 9 | 2 | 0 | 1 | 1 | 3 | *Allium cepa* |
| 562 | L-Citrulline | 175.19 | 6 | 4 | 4 | 0 | 1 | 0 | 0 | 2 | *Allium cepa* |
| 563 | 2-Tridecanone | 198.38 | 10 | 1 | 0 | 0 | 0 | 0 | 0 | 3 | *Allium cepa* |
| 564 | Phloroglucinaldehyde | 154.12 | 1 | 4 | 3 | 0 | 3 | 0 | 0 | 1 | *Allium cepa* |
| 565 | 2-Methyl-(E)-2-Butenal | 84.12 | 1 | 1 | 0 | 0 | 3 | 0 | 0 | 2 | *Allium cepa* |
| 566 | Dextrose | 271.24 | 1 | 5 | 4 | 0 | 0 | 0 | 0 | 0 | *Allium cepa* |
| 567 | Pelargonidin | 194.18 | 1 | 6 | 5 | 0 | 1 | 0 | 0 | 2 | *Allium cepa* |
| 568 | Propane-2-Thiol | 76.16 | 0 | 0 | 0 | 0 | 3 | 0 | 0 | 3 | *Allium cepa* |
| 569 | Peonidin | 301.27 | 2 | 6 | 4 | 0 | 0 | 0 | 0 | 0 | *Allium cepa* |
| 570 | 2-Methyl-(E)-2-Pentenal | 98.14 | 2 | 1 | 0 | 0 | 3 | 0 | 0 | 2 | *Allium cepa* |
| 571 | Quercetin-3,4'-Di-O-Glucoside | 626.52 | 7 | 17 | 11 | 3 | 4 | 1 | 1 | 4 | *Allium cepa* |
| 572 | 2-Amino-Hexanedioic Acid | 161.16 | 5 | 5 | 3 | 0 | 1 | 0 | 0 | 2 | *Allium cepa* |
| 573 | 2,4,6-Trihydroxybenzoic acid | 170.12 | 1 | 5 | 4 | 0 | 2 | 0 | 0 | 1 | *Allium cepa* |
| 574 | Prostaglandin A-1 | 336.47 | 13 | 4 | 2 | 0 | 0 | 1 | 0 | 0 | *Allium cepa* |
| 575 | Alliospiroside A | 708.88 | 4 | 12 | 6 | 3 | 3 | 1 | 1 | 5 | *Allium cepa* |
| 576 | Alliospiroside B | 738.9 | 5 | 13 | 7 | 3 | 3 | 1 | 1 | 5 | *Allium cepa* |
| 577 | Alliospiroside C | 724.88 | 4 | 13 | 7 | 3 | 3 | 1 | 1 | 5 | *Allium cepa* |
| 578 | Alliospiroside D | 754.9 | 5 | 14 | 8 | 3 | 3 | 1 | 1 | 5 | *Allium cepa* |
| 579 | Alliofuroside A | 889.03 | 11 | 18 | 11 | 3 | 4 | 2 | 1 | 5 | *Allium cepa* |
| 580 | Quercetin 3,4'-diglucoside | 626.52 | 7 | 17 | 11 | 3 | 4 | 1 | 1 | 4 | *Allium cepa* |
| 581 | Isorhamnetin 4'-glucoside | 478.4 | 5 | 12 | 7 | 2 | 0 | 1 | 1 | 3 | *Allium cepa* |
| 582 | cis-Ferulic acid | 194.18 | 3 | 4 | 2 | 0 | 0 | 0 | 0 | 1 | *Allium cepa* |
| 583 | Desacetyl-Beta-Cyclopyrethrosin | 264.32 | 0 | 4 | 2 | 0 | 0 | 0 | 0 | 0 | *Acacia auriculiformis* |
| 584 | Tatridin B | 264.32 | 0 | 4 | 2 | 0 | 0 | 0 | 0 | 0 | *Acacia auriculiformis* |
| 585 | Sinugibberodiol | 236.35 | 1 | 2 | 2 | 0 | 0 | 0 | 0 | 0 | *Acacia auriculiformis* |
| 586 | Elegalactone A | 264.32 | 0 | 4 | 2 | 0 | 0 | 0 | 0 | 0 | *Acacia auriculiformis* |
| 587 | 1-Epitatridin B | 264.32 | 0 | 4 | 2 | 0 | 0 | 0 | 0 | 0 | *Acacia auriculiformis* |
| 588 | Tamirin | 262.3 | 0 | 4 | 1 | 0 | 0 | 0 | 0 | 0 | *Acacia auriculiformis* |
| 589 | Umbelliferone | 162.14 | 0 | 3 | 1 | 0 | 1 | 0 | 0 | 1 | *Aegle marmelos* |
| 590 | Auraptene | 298.38 | 6 | 3 | 0 | 0 | 0 | 0 | 0 | 1 | *Aegle marmelos* |
| 591 | Aegeline | 297.35 | 7 | 3 | 2 | 0 | 0 | 0 | 0 | 0 | *Aegle marmelos* |
| 592 | Scoparone | 206.19 | 2 | 4 | 0 | 0 | 0 | 0 | 0 | 0 | *Aegle marmelos* |
| 593 | Gamma-Fagarine | 229.23 | 2 | 4 | 0 | 0 | 0 | 0 | 0 | 0 | *Aegle marmelos* |
| 594 | Marmesin | 246.26 | 1 | 4 | 1 | 0 | 0 | 0 | 0 | 0 | *Aegle marmelos* |
| 595 | Dehydromarmeline | 335.44 | 9 | 2 | 1 | 0 | 0 | 0 | 0 | 1 | *Aegle marmelos* |
| 596 | Anhydroaegeline | 279.33 | 6 | 2 | 1 | 0 | 0 | 0 | 0 | 0 | *Aegle marmelos* |
| 597 | Aegelinoside A | 459.44 | 10 | 8 | 5 | 0 | 0 | 0 | 1 | 0 | *Aegle marmelos* |
| 598 | Anhydromarmeline | 332.42 | 8 | 2 | 1 | 0 | 0 | 0 | 0 | 1 | *Aegle marmelos* |
| 599 | Aegelinoside B | 459.49 | 10 | 8 | 5 | 0 | 0 | 0 | 1 | 0 | *Aegle marmelos* |
| 600 | Doxorubicin | 543.52 | 5 | 12 | 6 | 3 | 2 | 1 | 1 | 3 | *Annona muricata* |
| 601 | Bullatacin | 622.92 | 25 | 7 | 3 | 1 | 4 | 1 | 1 | 3 | *Annona muricata* |
| 602 | Asimicin | 622.92 | 25 | 7 | 3 | 1 | 4 | 1 | 1 | 3 | *Annona muricata* |
| 603 | Rhamnetin | 316.26 | 2 | 7 | 4 | 0 | 0 | 0 | 0 | 0 | *Annona muricata* |
| 604 | Folic Acid | 441.4 | 10 | 9 | 6 | 2 | 0 | 1 | 1 | 2 | *Annona muricata* |
| 605 | Annonacin | 596.88 | 26 | 7 | 4 | 1 | 4 | 1 | 1 | 2 | *Annona muricata* |
| 606 | Annomontacin | 624.93 | 28 | 7 | 4 | 1 | 4 | 1 | 1 | 3 | *Annona muricata* |
| 607 | Bullanin | 606.92 | 25 | 6 | 2 | 2 | 4 | 1 | 1 | 3 | *Annona muricata* |
| 608 | 1,3,7-Trihydroxyxanthone | 244.2 | 0 | 5 | 3 | 0 | 0 | 0 | 0 | 0 | *Annona muricata* |
| 609 | Rel-Squamocin-G | 622.92 | 25 | 7 | 3 | 3 | 1 | 4 | 1 | 1 | *Annona muricata* |
| 610 | Annonacinone | 594.96 | 26 | 7 | 3 | 1 | 4 | 1 | 1 | 2 | *Annona muricata* |
| 611 | Corossolone | 578.86 | 26 | 6 | 2 | 2 | 4 | 1 | 1 | 2 | *Annona muricata* |
| 612 | Muricin A | 596.88 | 26 | 7 | 4 | 1 | 4 | 1 | 1 | 2 | *Annona muricata* |
| 613 | Annopentocin A | 612.88 | 26 | 8 | 5 | 1 | 4 | 1 | 2 | 3 | *Annona muricata* |
| 614 | Muricin B | 596.88 | 26 | 7 | 4 | 1 | 4 | 1 | 1 | 2 | *Annona muricata* |
| 615 | Goniothalamicin | 596.88 | 26 | 7 | 4 | 1 | 4 | 1 | 1 | 2 | *Annona muricata* |
| 616 | Annopentocin B | 612.88 | 26 | 8 | 5 | 1 | 4 | 1 | 2 | 3 | *Annona muricata* |
| 617 | Annopentocin C | 612.88 | 26 | 8 | 5 | 1 | 4 | 1 | 2 | 3 | *Annona muricata* |
| 618 | Cis-Annomontacin | 624.93 | 28 | 7 | 4 | 1 | 4 | 1 | 1 | 3 | *Annona muricata* |
| 619 | Annoglaucin | 638.92 | 25 | 8 | 4 | 1 | 4 | 1 | 1 | 3 | *Annona muricata* |
| 620 | Rel-Squamocin-H | 622.92 | 25 | 7 | 3 | 1 | 4 | 1 | 1 | 3 | *Annona muricata* |
| 621 | 5-Hydroxy-1,3-Dimethoxyxanthone | 272.25 | 2 | 5 | 1 | 0 | 0 | 0 | 0 | 0 | *Annona muricata* |
| 622 | Javoricin | 596.88 | 26 | 7 | 4 | 1 | 4 | 1 | 1 | 2 | *Annona muricata* |
| 623 | Arianacin | 596.88 | 26 | 7 | 4 | 1 | 4 | 1 | 1 | 2 | *Annona muricata* |
| 624 | Cis-Annonacin-10-One | 594.86 | 26 | 7 | 3 | 1 | 4 | 1 | 1 | 2 | *Annona muricata* |
| 625 | Cis-Goniothalamicin | 596.88 | 26 | 7 | 4 | 1 | 4 | 1 | 1 | 2 | *Annona muricata* |
| 626 | Annomuricin B | 612.88 | 26 | 8 | 5 | 1 | 4 | 1 | 2 | 3 | *Annona muricata* |
| 627 | Muricatocin B | 612.88 | 26 | 8 | 5 | 1 | 4 | 1 | 2 | 3 | *Annona muricata* |
| 628 | Arianacin Formal | 608.89 | 23 | 7 | 2 | 1 | 4 | 1 | 1 | 3 | *Annona muricata* |
| 629 | Muricatocin A | 612.88 | 26 | 8 | 5 | 1 | 4 | 1 | 2 | 3 | *Annona muricata* |
| 630 | Javoricin Formal | 608.89 | 23 | 7 | 2 | 1 | 4 | 1 | 1 | 3 | *Annona muricata* |
| 631 | Muricin C | 596.88 | 26 | 7 | 4 | 1 | 4 | 1 | 1 | 2 | *Annona muricata* |
| 632 | Cis-Annonacin Formal | 594.86 | 23 | 7 | 2 | 1 | 4 | 1 | 1 | 2 | *Annona muricata* |
| 633 | Xylomaticin | 624.93 | 28 | 7 | 4 | 1 | 4 | 1 | 1 | 3 | *Annona muricata* |
| 634 | Cis-Goniothalamicin Formal | 608.89 | 23 | 7 | 2 | 1 | 4 | 1 | 1 | 3 | *Annona muricata* |
| 635 | Annomuricin A | 612.88 | 26 | 8 | 5 | 1 | 4 | 1 | 2 | 3 | *Annona muricata* |
| 636 | Goniothalamicin Formal | 608.89 | 23 | 7 | 2 | 1 | 4 | 1 | 1 | 3 | *Annona muricata* |
| 637 | Corossoline | 580.88 | 26 | 6 | 3 | 2 | 4 | 1 | 1 | 2 | *Annona muricata* |
| 638 | Muricin D | 568.83 | 24 | 7 | 4 | 1 | 4 | 1 | 1 | 2 | *Annona muricata* |
| 639 | Muricin I | 606.92 | 27 | 6 | 3 | 2 | 4 | 1 | 1 | 3 | *Annona muricata* |
| 640 | Muricin H | 606.92 | 27 | 6 | 3 | 2 | 4 | 1 | 1 | 3 | *Annona muricata* |
| 641 | Muricatetrocin A/B | 580.88 | 26 | 6 | 3 | 2 | 4 | 1 | 1 | 2 | *Annona muricata* |
| 642 | Muricin F | 594.86 | 25 | 7 | 4 | 1 | 4 | 1 | 1 | 2 | *Annona muricata* |
| 643 | Longifolicin | 580.88 | 26 | 6 | 3 | 2 | 4 | 1 | 1 | 2 | *Annona muricata* |
| 644 | Annocatalin | 596.88 | 26 | 7 | 4 | 1 | 4 | 1 | 1 | 2 | *Annona muricata* |
| 645 | Rel-Squamocin-J | 578.86 | 23 | 6 | 2 | 2 | 4 | 1 | 1 | 2 | *Annona muricata* |
| 646 | 3,4,5-Trihydroxyxanthen-9-One | 434.91 | 10 | 6 | 3 | 0 | 1 | 0 | 1 | 1 | *Annona muricata* |
| 647 | Succinic Acid | 118.09 | 3 | 4 | 2 | 0 | 3 | 0 | 0 | 2 | *Averrhoa carambola* |
| 648 | Maleic Acid | 116.07 | 2 | 4 | 2 | 0 | 3 | 0 | 0 | 2 | *Averrhoa carambola* |
| 649 | Eudesmane-4Alpha,11-Diol | 240.38 | 1 | 2 | 2 | 0 | 0 | 0 | 0 | 0 | *Averrhoa carambola* |
| 650 | Mutatoxanthin | 584.87 | 9 | 3 | 2 | 2 | 4 | 0 | 1 | 1 | *Averrhoa carambola* |
| 651 | Fluorouracil | 130.08 | 0 | 3 | 2 | 0 | 3 | 0 | 0 | 2 | *Bauhinia purpurea* |
| 652 | Betulinic Acid | 456.7 | 2 | 3 | 2 | 1 | 3 | 0 | 1 | 1 | *Bauhinia purpurea* |
| 653 | Chrysin | 254.24 | 1 | 4 | 2 | 0 | 0 | 0 | 0 | 0 | *Bauhinia purpurea* |
| 654 | Lupeol | 426.72 | 1 | 1 | 1 | 1 | 3 | 0 | 1 | 2 | *Bauhinia purpurea* |
| 655 | 3-O-Methylquercetin | 316.26 | 2 | 7 | 4 | 0 | 0 | 0 | 0 | 0 | *Bauhinia purpurea* |
| 656 | Lupenone | 424.7 | 1 | 1 | 0 | 1 | 3 | 0 | 1 | 2 | *Bauhinia purpurea* |
| 657 | Demethoxymatteucinol | 284.31 | 1 | 4 | 2 | 0 | 0 | 0 | 0 | 0 | *Bauhinia purpurea* |
| 658 | 3-Methoxy-5-Phenethylphenol | 312.4 | 7 | 3 | 2 | 0 | 0 | 0 | 0 | 0 | *Bauhinia purpurea* |
| 659 | 7-Oxositosterol | 428.69 | 6 | 2 | 1 | 1 | 3 | 0 | 1 | 1 | *Bauhinia purpurea* |
| 660 | Dihydropinosylvin | 214.26 | 3 | 2 | 2 | 0 | 0 | 0 | 0 | 0 | *Bauhinia purpurea* |
| 661 | Batatasin Iv | 244.29 | 4 | 3 | 2 | 0 | 0 | 0 | 0 | 0 | *Bauhinia purpurea* |
| 662 | 5,7-Dihydroxy-6-Methyl-2-Phenylchromen-4-One | 268.26 | 1 | 4 | 2 | 0 | 0 | 0 | 0 | 0 | *Bauhinia purpurea* |
| 663 | Phosphoenolpyruvate | 168.04 | 3 | 6 | 3 | 0 | 2 | 0 | 0 | 2 | *Bauhinia purpurea* |
| 664 | Bauhinoxepin I | 428.69 | 6 | 2 | 1 | 1 | 3 | 0 | 1 | 1 | *Bauhinia purpurea* |
| 665 | Bauhinoxepin H | 214.26 | 3 | 2 | 2 | 0 | 0 | 0 | 0 | 0 | *Bauhinia purpurea* |
| 666 | Bauhinoxepin F | 244.29 | 4 | 3 | 2 | 0 | 0 | 0 | 0 | 0 | *Bauhinia purpurea* |
| 667 | Bauhiniastatin 1 | 268.26 | 1 | 4 | 2 | 0 | 0 | 0 | 0 | 0 | *Bauhinia purpurea* |
| 668 | Bauhiniastatin 3 | 168.04 | 3 | 6 | 3 | 0 | 2 | 0 | 0 | 2 | *Bauhinia purpurea* |
| 669 | Bauhinoxepin E | 302.32 | 2 | 5 | 2 | 0 | 0 | 0 | 0 | 0 | *Bauhinia purpurea* |
| 670 | Bauhiniastatin 2 | 300.31 | 2 | 5 | 2 | 0 | 0 | 0 | 0 | 0 | *Bauhinia purpurea* |
| 671 | Pacharin | 270.28 | 1 | 4 | 2 | 0 | 0 | 0 | 0 | 0 | *Bauhinia purpurea* |
| 672 | Strobopinin | 270.28 | 1 | 4 | 2 | 0 | 0 | 0 | 0 | 0 | *Bauhinia purpurea* |
| 673 | Bauhinoxepin J | 256.25 | 1 | 4 | 0 | 0 | 0 | 0 | 0 | 0 | *Bauhinia purpurea* |
| 674 | 2-(3,5-Dimethoxyphenethyl)Phenol | 258.31 | 5 | 3 | 1 | 0 | 0 | 0 | 0 | 0 | *Bauhinia purpurea* |
| 675 | Bauhinol E | 258.31 | 4 | 3 | 2 | 0 | 0 | 0 | 0 | 0 | *Bauhinia purpurea* |
| 676 | Bauhibenzofurin A | 272.3 | 3 | 4 | 1 | 0 | 0 | 0 | 0 | 0 | *Bauhinia purpurea* |
| 677 | Bauhinoxepin C | 272.3 | 1 | 4 | 2 | 0 | 0 | 0 | 0 | 0 | *Bauhinia purpurea* |
| 678 | Bauhinoxepin D | 272.3 | 1 | 4 | 2 | 0 | 0 | 0 | 0 | 0 | *Bauhinia purpurea* |
| 679 | Bauhinoxepin G | 272.3 | 1 | 4 | 2 | 0 | 0 | 0 | 0 | 0 | *Bauhinia purpurea* |
| 680 | 5,7-Dihydroxychromone | 258.31 | 4 | 3 | 2 | 0 | 0 | 0 | 0 | 0 | *Bauhinia purpurea* |
| 681 | Bauhiniastatin 4 | 270.28 | 1 | 4 | 2 | 0 | 0 | 0 | 0 | 0 | *Bauhinia purpurea* |
| 682 | Trilinolein | 879.38 | 50 | 6 | 0 | 2 | 4 | 1 | 1 | 3 | *Bauhinia purpurea* |
| 683 | Theophylline | 180.16 | 0 | 3 | 1 | 0 | 1 | 0 | 0 | 1 | *Camellia sinensis* |
| 684 | Phenothiazine | 199.27 | 0 | 0 | 1 | 0 | 0 | 0 | 0 | 1 | *Camellia sinensis* |
| 685 | Daidzein | 254.24 | 1 | 4 | 2 | 0 | 0 | 0 | 0 | 0 | *Camellia sinensis* |
| 686 | Orlistat | 495.73 | 24 | 5 | 1 | 1 | 4 | 1 | 1 | 2 | *Camellia sinensis* |
| 687 | Phenethylisothiocyanate | 163.24 | 3 | 1 | 0 | 0 | 0 | 0 | 0 | 1 | *Camellia sinensis* |
| 688 | Epigallocatechin | 306.27 | 1 | 7 | 6 | 1 | 0 | 0 | 0 | 1 | *Camellia sinensis* |
| 689 | Chlorogenic Acid | 354.31 | 5 | 9 | 6 | 1 | 1 | 1 | 1 | 2 | *Camellia sinensis* |
| 690 | Hyperoside | 464.38 | 4 | 12 | 8 | 2 | 1 | 1 | 1 | 3 | *Camellia sinensis* |
| 691 | (E)-Hex-2-Enal | 98.14 | 3 | 1 | 0 | 0 | 3 | 0 | 0 | 2 | *Camellia sinensis* |
| 692 | Theobromine | 180.16 | 0 | 3 | 1 | 0 | 1 | 0 | 0 | 1 | *Camellia sinensis* |
| 693 | Santin | 344.32 | 4 | 7 | 2 | 0 | 0 | 0 | 0 | 0 | *Camellia sinensis* |
| 694 | Beta-Ionone | 192.3 | 2 | 1 | 0 | 0 | 0 | 0 | 0 | 2 | *Camellia sinensis* |
| 695 | Procyanidin C1 | 866.77 | 5 | 18 | 15 | 3 | 3 | 1 | 1 | 5 | *Camellia sinensis* |
| 696 | Gallocatechin Gallate | 458.37 | 4 | 11 | 8 | 2 | 0 | 1 | 1 | 3 | *Camellia sinensis* |
| 697 | Citral | 152.23 | 4 | 1 | 0 | 0 | 1 | 0 | 0 | 2 | *Camellia sinensis* |
| 698 | (E)-2-Heptenal | 112.17 | 4 | 1 | 0 | 0 | 2 | 0 | 0 | 2 | *Camellia sinensis* |
| 699 | Furfuryl Alcohol | 98.1 | 1 | 2 | 1 | 0 | 3 | 0 | 0 | 1 | *Camellia sinensis* |
| 700 | Epiafzelechin | 274.27 | 1 | 5 | 4 | 0 | 0 | 0 | 0 | 0 | *Camellia sinensis* |
| 701 | 2-(4-Methylcyclohex-3-En-1-Yl) Propan-2-Ol | 154.25 | 1 | 1 | 1 | 0 | 1 | 0 | 0 | 2 | *Camellia sinensis* |
| 702 | 1-Pentanol | 88.15 | 3 | 1 | 1 | 0 | 3 | 0 | 0 | 2 | *Camellia sinensis* |
| 703 | Trifolin | 448.38 | 4 | 11 | 7 | 2 | 0 | 1 | 1 | 3 | *Camellia sinensis* |
| 704 | Egonol | 326.34 | 5 | 5 | 1 | 0 | 0 | 0 | 0 | 0 | *Camellia sinensis* |
| 705 | Chrysene | 228.29 | 0 | 0 | 0 | 1 | 0 | 0 | 0 | 2 | *Camellia sinensis* |
| 706 | Tricin | 330.29 | 3 | 7 | 3 | 0 | 0 | 0 | 0 | 0 | *Camellia sinensis* |
| 707 | Isomyricitrin | 480.38 | 4 | 13 | 9 | 2 | 2 | 1 | 1 | 3 | *Camellia sinensis* |
| 708 | 3,7,11-Trimethyldodeca-1,6,10-Trien-3-Ol | 222.37 | 7 | 1 | 1 | 0 | 0 | 0 | 0 | 1 | *Camellia sinensis* |
| 709 | Chakasaponin Ii | 1273.41 | 17 | 27 | 14 | 3 | 4 | 2 | 1 | 6 | *Camellia sinensis* |
| 710 | 3-O-Caffeoylquinic Acid | 354.31 | 5 | 9 | 6 | 1 | 1 | 1 | 1 | 2 | *Camellia sinensis* |
| 711 | 23-Hydroxyursolic Acid | 472.7 | 2 | 4 | 3 | 1 | 3 | 0 | 1 | 1 | *Camellia sinensis* |
| 712 | Theasaponin E1 | 1231.33 | 17 | 27 | 13 | 3 | 4 | 2 | 1 | 6 | *Camellia sinensis* |
| 713 | Floratheasaponin A | 1287.44 | 17 | 27 | 14 | 3 | 4 | 2 | 1 | 6 | *Camellia sinensis* |
| 714 | Pyrrole-2-Carboxylate | 111.1 | 1 | 2 | 2 | 0 | 3 | 0 | 0 | 1 | *Camellia sinensis* |
| 715 | Cis-Jasmone | 164.24 | 3 | 1 | 0 | 0 | 0 | 0 | 0 | 2 | *Camellia sinensis* |
| 716 | (E)-Linalool Oxide | 170.25 | 2 | 2 | 1 | 0 | 0 | 0 | 0 | 1 | *Camellia sinensis* |
| 717 | 2-O-(beta-L-Arabinopyranosyl)-myoinositol | 312.27 | 2 | 10 | 8 | 1 | 1 | 1 | 1 | 3 | *Camellia sinensis* |
| 718 | Degalloyl theasinensin F | 594.52 | 3 | 13 | 11 | 3 | 2 | 1 | 1 | 3 | *Camellia sinensis* |
| 719 | Epigallocatechin 3,5-digallate | 610.48 | 7 | 15 | 10 | 3 | 2 | 1 | 1 | 4 | *Camellia sinensis* |
| 720 | Epigallocatechin 3-caffeate | 668.41 | 5 | 10 | 7 | 1 | 0 | 1 | 1 | 2 | *Camellia sinensis* |
| 721 | Theaflagallin | 499.34 | 1 | 9 | 7 | 1 | 0 | 1 | 1 | 2 | *Camellia sinensis* |
| 722 | Theasinensin B | 762.62 | 6 | 18 | 14 | 3 | 3 | 1 | 1 | 4 | *Camellia sinensis* |
| 723 | Theasinensin E | 610.52 | 3 | 14 | 12 | 3 | 2 | 1 | 1 | 4 | *Camellia sinensis* |
| 724 | Theasinensin D | 914.73 | 9 | 22 | 16 | 3 | 3 | 1 | 1 | 5 | *Camellia sinensis* |
| 725 | Theasinensin F | 898.73 | 9 | 21 | 15 | 3 | 3 | 1 | 1 | 5 | *Camellia sinensis* |
| 726 | S-Methyl-L-methionine | 164.25 | 4 | 3 | 2 | 0 | 0 | 0 | 0 | 2 | *Camellia sinensis* |
| 727 | 1,4,6-Trigalloyl-beta-D-glucopyranose | 636.47 | 10 | 18 | 11 | 3 | 2 | 1 | 1 | 4 | *Camellia sinensis* |
| 728 | 8-C-Ascorbylepigallocatechin 3-gallate | 632.48 | 6 | 17 | 11 | 3 | 3 | 1 | 1 | 4 | *Camellia sinensis* |
| 729 | Strictinin | 634.45 | 3 | 18 | 11 | 3 | 2 | 1 | 1 | 4 | *Camellia sinensis* |
| 730 | Theogallin | 344.27 | 4 | 10 | 7 | 1 | 1 | 1 | 1 | 2 | *Camellia sinensis* |
| 731 | Oolonghomobisflavan A | 928.75 | 10 | 22 | 16 | 3 | 3 | 1 | 1 | 5 | *Camellia sinensis* |
| 732 | Oolonghomobisflavan B | 928.75 | 10 | 22 | 16 | 3 | 3 | 1 | 1 | 5 | *Camellia sinensis* |
| 733 | Germanicol | 426.72 | 0 | 1 | 1 | 1 | 3 | 0 | 1 | 2 | *Camellia sinensis* |
| 734 | Taraxerol | 426.72 | 0 | 1 | 1 | 1 | 3 | 0 | 1 | 2 | *Camellia sinensis* |
| 735 | Procyanidin B5 | 578.52 | 3 | 12 | 10 | 3 | 2 | 1 | 1 | 3 | *Camellia sinensis* |
| 736 | Procyanidin B7 | 578.52 | 3 | 12 | 10 | 3 | 2 | 1 | 1 | 3 | *Camellia sinensis* |
| 737 | Prodelphinidin B3 | 610.52 | 3 | 14 | 12 | 3 | 2 | 1 | 1 | 4 | *Camellia sinensis* |
| 738 | Theaflavin 3,3'-digallate | 878.7 | 8 | 20 | 13 | 3 | 3 | 1 | 1 | 5 | *Camellia sinensis* |
| 739 | Theaflavin 3-gallate | 716.6 | 5 | 16 | 11 | 3 | 3 | 1 | 1 | 4 | *Camellia sinensis* |
| 740 | Avicularin | 434.35 | 4 | 11 | 7 | 2 | 0 | 1 | 1 | 3 | *Camellia sinensis* |
| 741 | Pulmatin | 416.38 | 3 | 9 | 5 | 0 | 0 | 1 | 1 | 1 | *Cassia fistula* |
| 742 | 1,8-Dihydroxy-3-Methyl-4A,9A-Dihydroanthracene-9,10-Dione | 256.25 | 0 | 4 | 2 | 0 | 0 | 0 | 0 | 0 | *Cassia fistula* |
| 743 | (S)-Reticuline | 329.39 | 4 | 5 | 2 | 0 | 0 | 0 | 0 | 0 | *Cinnamomum camphora* |
| 744 | Debromoaplysiatoxin | 592.72 | 7 | 10 | 3 | 1 | 3 | 1 | 1 | 0 | *Cinnamomum camphora* |
| 745 | Procyanidin B1 | 578.52 | 3 | 12 | 10 | 3 | 2 | 1 | 1 | 3 | *Cinnamomum camphora* |
| 746 | Styrene | 104.15 | 1 | 0 | 0 | 0 | 3 | 0 | 0 | 2 | *Cinnamomum camphora* |
| 747 | Catechin-(4Alpha->6)-Epicatechin | 578.52 | 3 | 12 | 10 | 0 | 3 | 0 | 0 | 2 | *Cinnamomum camphora* |
| 748 | Aplysiatoxin | 671.61 | 7 | 10 | 3 | 1 | 3 | 1 | 1 | 1 | *Cinnamomum camphora* |
| 749 | Norboldine | 2 | 5 | 3 | 0 | 0 | 0 | 0 | 0 | 0 | *Cinnamomum camphora* |
| 750 | 4,7,7-Trimethylbicyclo[2.2.1]Heptan-3-One | 152.23 | 0 | 1 | 0 | 0 | 1 | 0 | 0 | 2 | *Cinnamomum camphora* |
| 751 | Obtusilactone A | 308.46 | 12 | 3 | 1 | 0 | 0 | 1 | 0 | 1 | *Cinnamomum camphora* |
| 752 | Obtusilactone | 278.39 | 10 | 3 | 1 | 0 | 0 | 0 | 0 | 1 | *Cinnamomum camphora* |
| 753 | Isoobutasilactone | 278.39 | 10 | 3 | 1 | 0 | 0 | 0 | 0 | 1 | *Cinnamomum camphora* |
| 754 | 1-Methoxy-4-[(Z)-Prop-1-Enyl]Benzene | 148.2 | 2 | 1 | 0 | 0 | 1 | 0 | 0 | 2 | *Cinnamomum camphora* |
| 755 | (6Z)-3,7,11-Trimethyldodeca-1,6,10-Trien-3-Ol | 222.37 | 7 | 1 | 1 | 0 | 0 | 0 | 0 | 1 | *Cinnamomum camphora* |
| 756 | (-)-Fenchone | 152.23 | 0 | 1 | 0 | 0 | 1 | 0 | 0 | 2 | *Cinnamomum camphora* |
| 757 | Azulene | 128.17 | 0 | 0 | 0 | 0 | 2 | 0 | 0 | 2 | *Cinnamomum camphora* |
| 758 | Isomahubannolide-23 | 364.56 | 16 | 3 | 1 | 1 | 1 | 1 | 1 | 2 | *Cinnamomum camphora* |
| 759 | Malyngamide M | 450.05 | 16 | 3 | 1 | 1 | 3 | 1 | 1 | 2 | *Cinnamomum camphora* |
| 760 | Malyngamide N | 466.05 | 16 | 4 | 0 | 0 | 3 | 1 | 0 | 2 | *Cinnamomum camphora* |
| 761 | Paulownin | 370.35 | 2 | 7 | 1 | 0 | 0 | 0 | 0 | 0 | *Cinnamomum camphora* |
| 762 | Cinnamaldehyde | 132.16 | 2 | 1 | 0 | 0 | 2 | 0 | 0 | 0 | *Cinnamomum tamala* |
| 763 | Xanthyletin | 228.24 | 0 | 3 | 0 | 0 | 0 | 0 | 0 | 0 | *Citrus aurantifolia* |
| 764 | 5,7,8,3',4'-Pentamethoxyflavone | 372.27 | 6 | 7 | 0 | 0 | 0 | 0 | 0 | 2 | *Citrus aurantifolia* |
| 765 | Linalool | 154.25 | 4 | 1 | 1 | 0 | 1 | 0 | 0 | 1 | *Coriandrum sativum* |
| 766 | 2,3-Diphenylcycloprop-2-En-1-One | 206.24 | 2 | 1 | 0 | 0 | 0 | 0 | 0 | 2 | *Coriandrum sativum* |
| 767 | 1-Methyl-4-Prop-1-En-2-Ylcyclohexene | 136.23 | 1 | 0 | 0 | 0 | 1 | 0 | 0 | 1 | *Coriandrum sativum* |
| 768 | Linalyl Acetate | 196.29 | 6 | 2 | 0 | 0 | 0 | 0 | 0 | 2 | *Coriandrum sativum* |
| 769 | Toluene | 92.14 | 0 | 0 | 0 | 0 | 3 | 0 | 0 | 2 | *Coriandrum sativum* |
| 770 | Pentane | 72.15 | 2 | 0 | 0 | 0 | 3 | 0 | 0 | 2 | *Coriandrum sativum* |
| 771 | (1S,3R,4S)-4,7,7-Trimethylbicyclo[2.2.1]Heptan-3-Ol | 154.25 | 0 | 1 | 1 | 0 | 1 | 0 | 0 | 0 | *Coriandrum sativum* |
| 772 | 4-[(3,3-Dimethyloxiran-2-Yl)Methoxy]Furo[3,2-G]Chromen-7-One | 286.28 | 3 | 5 | 0 | 0 | 0 | 0 | 0 | 2 | *Coriandrum sativum* |
| 773 | Undecanal | 170.29 | 9 | 1 | 0 | 0 | 0 | 0 | 0 | 2 | *Coriandrum sativum* |
| 774 | (1S,5S)-4,6,6-Trimethylbicyclo[3.1.1]Hept-3-Ene | 136.23 | 0 | 0 | 0 | 1 | 0 | 0 | 0 | 0 | *Coriandrum sativum* |
| 775 | Citroside B | 386.44 | 4 | 8 | 5 | 0 | 1 | 0 | 1 |  | *Coriandrum sativum* |
| 776 | Adonitol | 152.15 | 4 | 5 | 5 | 0 | 3 | 0 | 0 | 2 | *Coriandrum sativum* |
| 777 | Tetradecanal | 212.37 | 12 | 1 | 0 | 0 | 0 | 1 | 0 | 2 | *Coriandrum sativum* |
| 778 | (1S,5S)-6,6-Dimethyl-4-Methylidenebicyclo[3.1.1]Heptane | 136.23 | 0 | 0 | 0 | 1 | 1 | 0 | 0 | 2 | *Coriandrum sativum* |
| 779 | 2-(4-Methylphenyl)Propan-2-Ol | 150.22 | 1 | 1 | 1 | 0 | 1 | 0 | 0 | 2 | *Coriandrum sativum* |
| 780 | Hexan-3-Ol | 102.17 | 3 | 1 | 1 | 0 | 2 | 0 | 0 | 1 | *Coriandrum sativum* |
| 781 | Petroselenic Acid | 282.46 | 15 | 2 | 1 | 1 | 1 | 1 | 1 | 1 | *Coriandrum sativum* |
| 782 | Pristimerin | 464.64 | 2 | 4 | 1 | 1 | 3 | 0 | 1 | 3 | *Cheilocostus speciosus* |
| 783 | Paclitaxel | 853.91 | 15 | 14 | 4 | 2 | 3 | 2 | 1 | 2 | *Curcuma longa* |
| 784 | Farnesol | 222.37 | 7 | 1 | 1 | 0 | 0 | 0 | 0 | 0 | *Curcuma longa* |
| 785 | Chlorothalonil | 265.91 | 0 | 2 | 0 | 0 | 1 | 0 | 0 | 1 | *Curcuma longa* |
| 786 | Cyclovalone | 366.41 | 4 | 5 | 2 | 0 | 0 | 0 | 0 | 0 | *Curcuma longa* |
| 787 | Arachidonic Acid | 304.47 | 14 | 2 | 1 | 1 | 1 | 1 | 1 | 0 | *Curcuma longa* |
| 788 | 1,5-Bis(4-Hydroxy-3-Methoxyphenyl)-1,4-Pentadien-3-One | 326.34 | 6 | 5 | 2 | 0 | 0 | 0 | 0 | 3 | *Curcuma longa* |
| 789 | Congo Red | 696.66 | 7 | 10 | 2 | 2 | 3 | 1 | 2 | 0 | *Curcuma longa* |
| 790 | Bisdemethoxycurcumin | 308.33 | 6 | 4 | 2 | 0 | 0 | 0 | 0 | 0 | *Curcuma longa* |
| 791 | Flavanone | 224.25 | 1 | 2 | 0 | 0 | 0 | 0 | 0 | 0 | *Curcuma longa* |
| 792 | Demethoxycurcumin | 338.35 | 7 | 5 | 2 | 0 | 0 | 0 | 0 | 2 | *Curcuma longa* |
| 793 | (+)-Curcuphenol | 218.33 | 4 | 1 | 1 | 0 | 0 | 0 | 0 | 1 | *Curcuma longa* |
| 794 | Ligustrazine | 136.19 | 0 | 2 | 0 | 0 | 1 | 0 | 0 | 1 | *Curcuma longa* |
| 795 | 4-[(Z)-2-(3-Methoxyphenyl)Ethenyl]Phenol | 295.16 | 3 | 2 | 1 | 1 | 0 | 0 | 0 | 2 | *Curcuma longa* |
| 796 | Terpineol | 154.25 | 1 | 1 | 1 | 0 | 1 | 0 | 0 | 0 | *Curcuma longa* |
| 797 | Dichlofluanid | 333.23 | 5 | 4 | 0 | 0 | 0 | 0 | 0 | 2 | *Curcuma longa* |
| 798 | Benzaldehyde | 106.12 | 1 | 1 | 0 | 0 | 3 | 0 | 0 | 2 | *Curcuma longa* |
| 799 | 1-Methyl-4-(6-Methylhepta-1,5-Dien-2-Yl)Cyclohexene | 204.35 | 4 | 0 | 0 | 0 | 0 | 0 | 0 | 2 | *Curcuma longa* |
| 800 | Docusate | 422.58 | 18 | 7 | 1 | 0 | 0 | 1 | 0 | 2 | *Curcuma longa* |
| 801 | 1-Heptanol | 116.2 | 5 | 1 | 1 | 0 | 2 | 0 | 0 | 1 | *Curcuma longa* |
| 802 | Zerumbone | 218.33 | 0 | 1 | 0 | 0 | 0 | 0 | 0 | 0 | *Curcuma longa* |
| 803 | Tetrahydrocurcumin | 372.41 | 10 | 6 | 2 | 0 | 0 | 0 | 0 | 2 | *Curcuma longa* |
| 804 | Ethylbenzene | 106.17 | 1 | 0 | 0 | 0 | 3 | 0 | 0 |  | *Curcuma longa* |
| 805 | L-Carvone | 150.22 | 1 | 1 | 0 | 0 | 1 | 0 | 0 | 1 | *Curcuma longa* |
| 806 | Isofuranodiene | 216.32 | 0 | 1 | 0 | 0 | 0 | 0 | 0 | 1 | *Curcuma longa* |
| 807 | 2-Methyl-3-[(E)-3,7,11,15-Tetramethylhexadec-2-EnylNaphthalene-1,4-Dione | 450.7 | 14 | 2 | 0 | 1 | 3 | 1 | 1 | 1 | *Curcuma longa* |
| 808 | Alloaromadendrene | 204.35 | 0 | 0 | 0 | 1 | 0 | 0 | 0 | 1 | *Curcuma longa* |
| 809 | (E)-Hexadec-9-Enoic Acid | 254.41 | 13 | 2 | 1 | 0 | 0 | 1 | 0 | 2 | *Curcuma longa* |
| 810 | Zedoarondiol | 304.58 | 20 | 2 | 11 | 1 | 1 | 1 | 1 |  | *Curcuma longa* |
| 811 | Tetramethoxycurcumin | 396.43 | 10 | 6 | 0 | 0 | 0 | 0 | 0 | 0 | *Curcuma longa* |
| 812 | Menthofuran | 150.22 | 0 | 1 | 0 | 0 | 1 | 0 | 0 | 2 | *Curcuma longa* |
| 813 | Citronellyl Acetate | 198.3 | 7 | 2 | 0 | 0 | 0 | 0 | 0 | 1 | *Curcuma longa* |
| 814 | Labdadienedial | 302.45 | 5 | 2 | 0 | 0 | 0 | 0 | 0 | 0 | *Curcuma longa* |
| 815 | Turmerone | 218.33 | 4 | 1 | 0 | 0 | 0 | 0 | 0 | 1 | *Curcuma longa* |
| 816 | Dehydrodeguelin | 392.4 | 2 | 6 | 0 | 0 | 0 | 0 | 0 | 0 | *Curcuma longa* |
| 817 | 1,7-Bis(4-Hydroxyphenyl)-1,4,6-Heptatrien-3-One | 292.33 | 5 | 3 | 2 | 0 | 0 | 0 | 0 | 0 | *Curcuma longa* |
| 818 | Ent-Spathulenol | 220.35 | 0 | 1 | 1 | 0 | 0 | 0 | 0 | 1 | *Curcuma longa* |
| 819 | 2-Methylpentane | 86.18 | 2 | 0 | 0 | 0 | 2 | 0 | 0 | 2 | *Curcuma longa* |
| 820 | O-Demethyldemethoxycurcumin | 324.33 | 6 | 5 | 3 | 0 | 0 | 0 | 0 | 0 | *Curcuma longa* |
| 821 | Mono-O-Demethylcurcumin | 354.35 | 7 | 6 | 3 | 0 | 0 | 0 | 0 | 0 | *Curcuma longa* |
| 822 | 2-(4-Methylcyclohex-3-En-1-Yl)Propan-2-Yl Acetate | 196.29 | 3 | 2 | 0 | 0 | 0 | 0 | 0 | 1 | *Curcuma longa* |
| 823 | Letestuianin C | 312.36 | 8 | 4 | 2 | 0 | 0 | 0 | 0 | 0 | *Curcuma longa* |
| 824 | (3S)-3,7-Dimethyloct-6-En-1-Ol | 156.27 | 5 | 1 | 1 | 0 | 1 | 0 | 0 | 2 | *Curcuma longa* |
| 825 | Cyclopentane | 70.13 | 0 | 0 | 0 | 0 | 3 | 0 | 0 | 2 | *Curcuma longa* |
| 826 | 4-Ethenyl-2-Methoxyphenol | 150.17 | 2 | 2 | 1 | 0 | 1 | 0 | 0 | 1 | *Curcuma longa* |
| 827 | Syn-Benzaldoxime | 121.14 | 1 | 2 | 1 | 0 | 3 | 0 | 0 | 1 | *Curcuma longa* |
| 828 | 2-Methyl-5-Propan-2-Ylcyclohexa-1,3-Diene | 136.23 | 1 | 0 | 0 | 0 | 1 | 0 | 0 | 2 | *Curcuma longa* |
| 829 | Ocimene | 136.23 | 1 | 0 | 0 | 0 | 1 | 0 | 0 | 2 | *Curcuma longa* |
| 830 | Curcumenol | 234.33 | 0 | 2 | 1 | 0 | 0 | 0 | 0 | 0 | *Curcuma longa* |
| 831 | (4,7,7-Trimethyl-3-Bicyclo[2.2.1]Heptanyl) Acetate | 196.29 | 2 | 2 | 0 | 0 | 0 | 0 | 0 | 1 | *Curcuma longa* |
| 832 | Propanoyl Propanoate | 130.14 | 4 | 3 | 0 | 0 | 3 | 0 | 0 | 1 | *Curcuma longa* |
| 833 | Heptadecanoic Acid | 270.45 | 15 | 2 | 1 | 1 | 1 | 1 | 1 | 1 | *Curcuma longa* |
| 834 | Cyclooctanone | 126.2 | 0 | 1 | 0 | 0 | 2 | 0 | 0 | 2 | *Curcuma longa* |
| 835 | Sodium Caprylate | 166.19 | 6 | 2 | 0 | 0 | 0 | 0 | 0 | 1 | *Curcuma longa* |
| 836 | 1,2,3,5-Tetramethylbenzene | 134.22 | 0 | 0 | 0 | 1 | 1 | 0 | 0 | 2 | *Curcuma longa* |
| 837 | 3,7-Dimethyloct-6-Enal | 154.25 | 5 | 1 | 0 | 0 | 1 | 0 | 0 | 2 | *Curcuma longa* |
| 838 | Procurcumadiol | 250.33 | 0 | 3 | 2 | 0 | 0 | 0 | 0 | 0 | *Curcuma longa* |
| 839 | Ar-Turmerone | 216.32 | 4 | 1 | 0 | 0 | 0 | 0 | 0 | 1 | *Curcuma longa* |
| 840 | Alpha-Elemol | 222.37 | 3 | 1 | 1 | 0 | 0 | 0 | 0 | 1 | *Curcuma longa* |
| 841 | Azetidine-2-Carboxylic Acid | 101.1 | 1 | 3 | 2 | 0 | 4 | 0 | 0 | 3 | *Delonix regia* |
| 842 | Arbutin | 272.25 | 3 | 7 | 5 | 0 | 1 | 0 | 0 | 0 | *Diospyros peregrina* |
| 843 | Skimmianine | 259.26 | 3 | 5 | 0 | 0 | 0 | 0 | 0 | 0 | *Diospyros peregrina* |
| 844 | Xanthoxylin | 196.2 | 3 | 4 | 1 | 0 | 0 | 0 | 0 | 1 | *Diospyros peregrina* |
| 845 | Hydroxy-Alpha-Sanshool | 263.38 | 9 | 2 | 2 | 0 | 0 | 0 | 0 | 0 | *Diospyros peregrina* |
| 846 | Piperonylic Acid | 166.13 | 1 | 4 | 1 | 0 | 2 | 0 | 0 | 1 | *Diospyros peregrina* |
| 847 | Hydroxy-Beta-Sanshool | 263.38 | 9 | 2 | 2 | 0 | 0 | 0 | 0 | 0 | *Diospyros peregrina* |
| 848 | Piperitol | 356.37 | 3 | 6 | 1 | 0 | 0 | 0 | 0 | 0 | *Diospyros peregrina* |
| 849 | Pluviatilol | 356.37 | 3 | 6 | 1 | 0 | 0 | 0 | 0 | 0 | *Diospyros peregrina* |
| 850 | Laurifoline | 342.41 | 2 | 4 | 2 | 0 | 0 | 0 | 0 | 0 | *Diospyros peregrina* |
| 851 | Wighteone | 338.35 | 3 | 5 | 3 | 0 | 0 | 0 | 0 | 0 | *Erythrina variegata* |
| 852 | Alpinumisoflavone | 336.34 | 1 | 5 | 2 | 0 | 0 | 0 | 0 | 0 | *Erythrina variegata* |
| 853 | Erysovine | 299.36 | 2 | 4 | 1 | 0 | 0 | 0 | 0 | 0 | *Erythrina variegata* |
| 854 | Erysenegalensein E | 422.47 | 6 | 6 | 4 | 0 | 0 | 0 | 0 | 1 | *Erythrina variegata* |
| 855 | Metformin | 129.16 | 2 | 2 | 3 | 0 | 3 | 0 | 0 | 2 | *Eucalyptus camaldulensis* |
| 856 | (-)-Epicatechin | 290.287 | 1 | 6 | 5 | 0 | 0 | 0 | 0 | 0 | *Eucalyptus camaldulensis* |
| 857 | Mangostin | 410.46 | 5 | 6 | 3 | 0 | 0 | 0 | 0 | 1 | *Eucalyptus camaldulensis* |
| 858 | Garcinone E | 464.55 | 6 | 6 | 4 | 0 | 2 | 0 | 1 | 1 | *Eucalyptus camaldulensis* |
| 859 | Proanthocyanidin A2 | 576.5 | 2 | 12 | 9 | 3 | 2 | 1 | 1 | 3 | *Eucalyptus camaldulensis* |
| 860 | Norathyriol | 260.2 | 0 | 6 | 4 | 0 | 0 | 0 | 0 | 0 | *Eucalyptus camaldulensis* |
| 861 | 1,7-Dihydroxyxanthone | 228.2 | 0 | 4 | 2 | 0 | 0 | 0 | 0 | 0 | *Eucalyptus camaldulensis* |
| 862 | 3-Isomangostin | 410.46 | 3 | 6 | 2 | 0 | 0 | 0 | 0 | 1 | *Eucalyptus camaldulensis* |
| 863 | Beta-Mangostin | 424.49 | 6 | 6 | 2 | 0 | 0 | 0 | 0 | 1 | *Eucalyptus camaldulensis* |
| 864 | Mangostanin | 408.44 | 3 | 6 | 2 | 0 | 0 | 0 | 0 | 1 | *Eucalyptus camaldulensis* |
| 865 | Fuscaxanthone C | 458.51 | 7 | 6 | 1 | 0 | 1 | 0 | 0 | 1 | *Eucalyptus camaldulensis* |
| 866 | Proanthocyanidin A1 | 576.5 | 2 | 12 | 9 | 3 | 2 | 1 | 1 | 3 | *Eucalyptus camaldulensis* |
| 867 | Macluraxanthone | 394.42 | 2 | 6 | 3 | 0 | 0 | 0 | 0 | 1 | *Eucalyptus camaldulensis* |
| 868 | Garcinone D | 428.47 | 6 | 7 | 4 | 0 | 0 | 0 | 0 | 0 | *Eucalyptus camaldulensis* |
| 869 | Rubraxanthone | 410.46 | 6 | 6 | 3 | 0 | 0 | 0 | 0 | 1 | *Eucalyptus camaldulensis* |
| 870 | 6-Methylsalicylic Acid | 152.15 | 1 | 3 | 2 | 0 | 2 | 0 | 0 | 1 | *Eucalyptus camaldulensis* |
| 871 | 1,3,7-Trihydroxy-2-Prenylxanthone | 312.32 | 2 | 5 | 3 | 0 | 0 | 0 | 0 | 0 | *Eucalyptus camaldulensis* |
| 872 | Br-Xanthone A | 396.43 | 0 | 6 | 2 | 0 | 0 | 0 | 0 | 0 | *Eucalyptus camaldulensis* |
| 873 | Maclurin | 262.21 | 2 | 6 | 5 | 0 | 0 | 0 | 0 | 0 | *Eucalyptus camaldulensis* |
| 874 | 8-Deoxygartanin | 380.43 | 4 | 5 | 3 | 0 | 0 | 0 | 0 | 1 | *Eucalyptus camaldulensis* |
| 875 | 3,6,8-Trihydroxy-2-Methoxy-1-(3-Methylbut-2-Enyl)Xanthen-9-One | 478.58 | 8 | 6 | 3 | 0 | 2 | 0 | 1 | 1 | *Eucalyptus camaldulensis* |
| 876 | Astilbin | 450.39 | 3 | 11 | 7 | 2 | 0 | 1 | 1 | 3 | *Eucalyptus camaldulensis* |
| 877 | 1,3,6-Trihydroxy-2,4-Bis(3-Methylbut-2-Enyl)Xanthen-9-One | 380.43 | 4 | 5 | 3 | 0 | 0 | 0 | 0 | 1 | *Eucalyptus camaldulensis* |
| 878 | Tovophylline A | 462.53 | 4 | 6 | 3 | 0 | 2 | 0 | 1 | 1 | *Eucalyptus camaldulensis* |
| 879 | 1,3,7-Trihydroxy-2-Methoxyxanthone | 274.23 | 1 | 6 | 3 | 0 | 0 | 0 | 0 | 0 | *Eucalyptus camaldulensis* |
| 880 | Smeathxanthone A | 396.43 | 5 | 6 | 4 | 0 | 0 | 0 | 0 | 1 | *Eucalyptus camaldulensis* |
| 881 | 2,3,6,8-Tetrahydroxy-1-(3-Methylbut-2-Enyl)Xanthen-9-One | 328.32 | 2 | 6 | 4 | 0 | 0 | 0 | 0 | 0 | *Eucalyptus camaldulensis* |
| 882 | 1-Isomangostin | 410.46 | 3 | 6 | 2 | 0 | 0 | 0 | 0 | 1 | *Eucalyptus camaldulensis* |
| 883 | Mangostenone D | 396.43 | 2 | 6 | 3 | 0 | 0 | 0 | 0 | 1 | *Eucalyptus camaldulensis* |
| 884 | Cratoxyxanthone | 834.9 | 10 | 13 | 6 | 3 | 4 | 1 | 2 | 5 | *Eucalyptus camaldulensis* |
| 885 | Cudraxanthone | 326.34 | 3 | 5 | 2 | 0 | 0 | 0 | 0 | 0 | *Eucalyptus camaldulensis* |
| 886 | Mangostinone | 380.43 | 5 | 5 | 3 | 0 | 0 | 0 | 0 | 1 | *Eucalyptus camaldulensis* |
| 887 | 2,4,6,3',5'-Pentahydroxybenzophenone | 262.21 | 2 | 6 | 5 | 0 | 0 | 0 | 0 | 0 | *Eucalyptus camaldulensis* |
| 888 | 11Alpha-Mangostanin | 426.46 | 4 | 7 | 3 | 0 | 0 | 0 | 0 | 0 | *Eucalyptus camaldulensis* |
| 889 | Cudraxanthone G | 394.46 | 5 | 5 | 2 | 0 | 0 | 0 | 0 | 1 | *Eucalyptus camaldulensis* |
| 890 | 3,4,5,3'-Tetrahydroxybenzophenone | 246.22 | 2 | 5 | 4 | 0 | 0 | 0 | 0 | 0 | *Eucalyptus camaldulensis* |
| 891 | Demethylcalabaxanthone | 378.42 | 2 | 5 | 2 | 0 | 0 | 0 | 0 | 1 | *Eucalyptus camaldulensis* |
| 892 | 8-Hydroxycudraxanthone G | 410.46 | 5 | 6 | 3 | 0 | 0 | 0 | 0 | 1 | *Eucalyptus camaldulensis* |
| 893 | Epigalocatechin Gallate | 458.37 | 4 | 11 | 8 | 2 | 0 | 1 | 1 | 3 | *Garcinia mangostana* |
| 894 | Betulin | 442.72 | 2 | 2 | 2 | 1 | 3 | 0 | 1 | 1 | *Garcinia mangostana* |
| 895 | Aminoglutethimide | 232.28 | 2 | 2 | 2 | 0 | 0 | 0 | 0 | 0 | *Garcinia mangostana* |
| 896 | Phytate | 660.04 | 12 | 24 | 12 | 3 | 2 | 2 | 1 | 5 | *Garcinia mangostana* |
| 897 | Gartanin | 396.43 | 4 | 6 | 4 | 0 | 0 | 0 | 0 | 1 | *Garcinia mangostana* |
| 898 | Euxanthone | 228.2 | 0 | 4 | 2 | 0 | 0 | 0 | 0 | 0 | *Garcinia mangostana* |
| 899 | Di-O-Acetylmangostin | 494.53 | 9 | 8 | 1 | 0 | 2 | 0 | 0 | 1 | *Garcinia mangostana* |
| 900 | Di-O-Allylmangostin | 490.59 | 11 | 6 | 1 | 0 | 3 | 1 | 1 | 1 | *Garcinia mangostana* |
| 901 | Di-O-Isopropylmangostin | 494.62 | 9 | 6 | 1 | 0 | 4 | 0 | 1 | 1 | *Garcinia mangostana* |
| 902 | Di-O-Ethylmangostin | 466.57 | 9 | 6 | 1 | 0 | 2 | 0 | 1 | 1 | *Garcinia mangostana* |
| 903 | Di-O-Butylmangostin | 522.67 | 13 | 6 | 1 | 1 | 4 | 1 | 1 | 1 | *Garcinia mangostana* |
| 904 | Di-O-Methallylmangostin | 518.64 | 11 | 6 | 1 | 1 | 4 | 1 | 1 | 1 | *Garcinia mangostana* |
| 905 | Xanthene-9-Thione | 212.27 | 0 | 1 | 0 | 0 | 0 | 0 | 0 | 0 | *Garcinia mangostana* |
| 906 | Di-O-Propylmangostin | 494.62 | 11 | 6 | 1 | 0 | 4 | 1 | 1 | 1 | *Garcinia mangostana* |
| 907 | 11-Hydroxy-1-Isomangostin | 426.46 | 3 | 7 | 3 | 0 | 0 | 0 | 0 | 0 | *Garcinia mangostana* |
| 908 | 11-hydroxy-3-O-methyl-1-isomangostin | 440.49 | 4 | 7 | 2 | 0 | 0 | 0 | 0 | 0 | *Garcinia mangostana* |
| 909 | Mangostanol | 426.46 | 3 | 7 | 3 | 0 | 0 | 0 | 0 | 0 | *Garcinia mangostana* |
| 910 | Garcinone B | 394.42 | 2 | 6 | 3 | 0 | 0 | 0 | 0 | 1 | *Garcinia mangostana* |
| 911 | Geniposidic Acid | 374.34 | 5 | 10 | 6 | 1 | 1 | 1 | 1 | 3 | *Gmelina philippensis* |
| 912 | Gardoside | 374.34 | 4 | 10 | 6 | 1 | 1 | 1 | 1 | 3 | *Gmelina philippensis* |
| 913 | 8-Epiloganic Acid | 376.36 | 4 | 10 | 6 | 1 | 1 | 1 | 1 | 2 | *Gmelina philippensis* |
| 914 | Diphyllin | 380.35 | 3 | 7 | 1 | 0 | 0 | 0 | 0 | 0 | *Justicia gendarussa* |
| 915 | Patentiflorin A | 526.49 | 5 | 11 | 3 | 2 | 2 | 1 | 1 | 1 | *Justicia gendarussa* |
| 916 | Gnf-Pf-349 | 542.49 | 6 | 12 | 4 | 2 | 2 | 1 | 1 | 2 | *Justicia gendarussa* |
| 917 | 1,3,6-Tri-O-Galloyl-Beta-D-Glucose | 636.47 | 10 | 18 | 11 | 3 | 2 | 1 | 1 | 4 | *Lagerstroemia speciosa* |
| 918 | 2-Ethylhexan-1-Ol | 130.23 | 5 | 1 | 1 | 0 | 1 | 0 | 0 | 2 | *Lagerstroemia speciosa* |
| 919 | Maslinic Acid | 472.7 | 4 | 1 | 3 | 1 | 3 | 0 | 1 | 1 | *Leucas aspera* |
| 920 | Austrobailignan 6 | 328.4 | 6 | 4 | 1 | 0 | 0 | 0 | 0 | 1 | *Leucas aspera* |
| 921 | Asperphenamate | 506.59 | 14 | 4 | 2 | 2 | 2 | 1 | 0 | 1 | *Leucas aspera* |
| 922 | Berkeleyone A | 446.58 | 2 | 6 | 2 | 0 | 0 | 0 | 0 | 0 | *Leucas aspera* |
| 923 | Berkazaphilone C | 386.4 | 4 | 7 | 3 | 0 | 0 | 0 | 0 | 0 | *Leucas aspera* |
| 924 | Berkeleyone C | 458.54 | 6 | 7 | 2 | 0 | 0 | 0 | 0 | 0 | *Leucas aspera* |
| 925 | Berkeleyamide A | 303.4 | 7 | 3 | 2 | 0 | 0 | 0 | 0 | 0 | *Leucas aspera* |
| 926 | Berkeleyacetal B | 486.51 | 2 | 9 | 0 | 0 | 1 | 0 | 0 | 0 | *Leucas aspera* |
| 927 | Berkazaphilone B | 386.4 | 4 | 7 | 3 | 0 | 0 | 0 | 0 | 0 | *Leucas aspera* |
| 928 | Preaustinoid A | 444.56 | 2 | 6 | 1 | 0 | 0 | 0 | 0 | 0 | *Leucas aspera* |
| 929 | Mitorubrinol | 398.36 | 5 | 8 | 3 | 0 | 0 | 0 | 0 | 0 | *Leucas aspera* |
| 930 | T-Zeatin | 219.24 | 4 | 4 | 3 | 0 | 0 | 0 | 0 | 0 | *Madhuca longifolia* |
| 931 | Chikusetsusaponin Iva | 794.97 | 7 | 14 | 8 | 3 | 3 | 1 | 1 | 4 | *Madhuca longifolia* |
| 932 | D-Pipecolic Acid | 129.16 | 1 | 3 | 2 | 0 | 2 | 0 | 0 | 2 | *Madhuca longifolia* |
| 933 | Pentagalloyl Glucose | 940.68 | 16 | 26 | 15 | 3 | 3 | 2 | 1 | 5 | *Mangifera indica* |
| 934 | Propyl Gallate | 212.2 | 4 | 5 | 3 | 0 | 0 | 0 | 0 | 0 | *Mangifera indica* |
| 935 | Arctigenin | 372.41 | 7 | 6 | 1 | 0 | 0 | 0 | 0 | 0 | *Mangifera indica* |
| 936 | Mangiferin | 422.34 | 2 | 11 | 8 | 2 | 1 | 1 | 1 | 3 | *Mangifera indica* |
| 937 | Cycloartenol | 426.72 | 4 | 1 | 1 | 1 | 3 | 0 | 1 | 2 | *Mangifera indica* |
| 938 | Damnacanthal | 282.25 | 2 | 5 | 1 | 0 | 0 | 0 | 0 | 0 | *Mangifera indica* |
| 939 | Erythrodiol | 442.72 | 1 | 2 | 2 | 1 | 3 | 0 | 1 | 1 | *Mangifera indica* |
| 940 | Friedeline | 426.72 | 0 | 1 | 0 | 1 | 3 | 0 | 1 | 2 | *Mangifera indica* |
| 941 | Alpha-Amyrin | 426.72 | 0 | 1 | 1 | 1 | 3 | 0 | 1 | 2 | *Mangifera indica* |
| 942 | Lupenyl Acetate | 468.75 | 3 | 2 | 0 | 1 | 3 | 0 | 1 | 1 | *Mangifera indica* |
| 943 | Epifriedelinol | 428.73 | 0 | 1 | 1 | 1 | 3 | 0 | 1 | 2 | *Mangifera indica* |
| 944 | 1,3-Dihydroxypropan-2-Yl (Z)-Octadec-9-Enoate | 356.54 | 19 | 4 | 2 | 0 | 0 | 1 | 0 | 2 | *Mangifera indica* |
| 945 | Mangiferolic Acid | 456.7 | 5 | 3 | 2 | 1 | 3 | 0 | 1 | 1 | *Mangifera indica* |
| 946 | Propyl Benzoate | 164.2 | 4 | 2 | 0 | 0 | 0 | 0 | 0 | 1 | *Mangifera indica* |
| 947 | Taraxerone | 424.7 | 0 | 1 | 0 | 1 | 3 | 0 | 1 | 2 | *Mangifera indica* |
| 948 | Cycloartenone | 424.7 | 4 | 1 | 0 | 1 | 3 | 0 | 1 | 2 | *Mangifera indica* |
| 949 | Catechin Gallate | 442.37 | 4 | 10 | 7 | 1 | 0 | 1 | 1 | 2 | *Mangifera indica* |
| 950 | Rubrofusarin | 272.25 | 1 | 5 | 2 | 0 | 0 | 0 | 0 | 0 | *Mangifera indica* |
| 951 | Cycloartenol Acetate | 468.75 | 6 | 2 | 0 | 1 | 3 | 0 | 1 | 1 | *Mangifera indica* |
| 952 | Isomangiferolic Acid | 456.7 | 5 | 3 | 2 | 1 | 3 | 0 | 1 | 1 | *Mangifera indica* |
| 953 | Epi-Friedelinol | 428.73 | 0 | 1 | 1 | 1 | 3 | 0 | 1 | 2 | *Mangifera indica* |
| 954 | Diolein | 620.99 | 36 | 5 | 1 | 2 | 4 | 1 | 1 | 3 | *Mangifera indica* |
| 955 | Glyceryl Monooleate | 356.54 | 19 | 4 | 2 | 0 | 0 | 1 | 0 | 2 | *Mangifera indica* |
| 956 | Foliamangiferoside A | 422.38 | 5 | 10 | 7 | 1 | 1 | 1 | 1 | 2 | *Mangifera indica* |
| 957 | Ursolaldehyde | 440.7 | 1 | 2 | 1 | 1 | 3 | 0 | 1 | 1 | *Mangifera indica* |
| 958 | 4-O-Galloylchlorogenic Acid | 506.41 | 8 | 13 | 8 | 3 | 1 | 1 | 1 | 3 | *Manilkara zapota* |
| 959 | Methyl 4-O-Galloylchlorogenate | 520.44 | 9 | 13 | 7 | 3 | 1 | 1 | 1 | 3 | *Manilkara zapota* |
| 960 | Kaurenoic Acid | 302.45 | 1 | 2 | 1 | 1 | 0 | 0 | 0 | 1 | *Mikania micrantha* |
| 961 | 5,6-Dihydro Ergosterol | 398.66 | 4 | 1 | 1 | 1 | 2 | 0 | 1 | 2 | *Mikania micrantha* |
| 962 | Alpinetin | 270.28 | 2 | 4 | 1 | 0 | 0 | 0 | 0 | 0 | *Mikania micrantha* |
| 963 | Dihydromikanolide | 292.28 | 0 | 6 | 0 | 0 | 0 | 0 | 0 | 0 | *Mikania micrantha* |
| 964 | Mikanin | 344.32 | 4 | 7 | 2 | 0 | 0 | 0 | 0 | 0 | *Mikania micrantha* |
| 965 | Potassium Mikanin 3-Sulfate | 462.47 | 6 | 10 | 1 | 0 | 0 | 1 | 1 | 1 | *Mikania micrantha* |
| 966 | Xerantolide | 246.3 | 0 | 3 | 0 | 0 | 0 | 0 | 0 | 0 | *Mikania micrantha* |
| 967 | Scandenolide | 334.32 | 2 | 7 | 0 | 0 | 0 | 0 | 0 | 0 | *Mikania micrantha* |
| 968 | Taraxasterol Acetate | 468.75 | 2 | 2 | 0 | 1 | 3 | 0 | 1 | 1 | *Mikania micrantha* |
| 969 | Methyl Vanillate | 182.17 | 3 | 4 | 1 | 0 | 0 | 0 | 0 | 1 | *Moringa oleifera* |
| 970 | Resveratrol | 228.24 | 2 | 3 | 3 | 0 | 0 | 0 | 0 | 0 | *Morus alba* |
| 971 | Scopoletin | 192.17 | 1 | 4 | 1 | 0 | 0 | 0 | 0 | 1 | *Morus alba* |
| 972 | Morin | 302.24 | 1 | 7 | 5 | 0 | 0 | 0 | 0 | 0 | *Morus alba* |
| 973 | Duvoglustat | 163.17 | 1 | 5 | 5 | 0 | 1 | 0 | 0 | 1 | *Morus alba* |
| 974 | Taxifolin | 304.25 | 1 | 7 | 5 | 0 | 0 | 0 | 0 | 0 | *Morus alba* |
| 975 | Butein | 272.25 | 3 | 5 | 4 | 0 | 0 | 0 | 0 | 0 | *Morus alba* |
| 976 | Isobavachalcone | 324.37 | 5 | 4 | 3 | 0 | 0 | 0 | 0 | 1 | *Morus alba* |
| 977 | Cis-Resveratrol | 228.24 | 2 | 3 | 3 | 0 | 0 | 0 | 0 | 0 | *Morus alba* |
| 978 | Quercitrin | 448.38 | 3 | 11 | 7 | 2 | 0 | 1 | 1 | 3 | *Morus alba* |
| 979 | Guaiacol | 124.14 | 1 | 2 | 1 | 0 | 3 | 0 | 0 | 1 | *Morus alba* |
| 980 | Morusin | 420.45 | 3 | 6 | 3 | 0 | 0 | 0 | 0 | 1 | *Morus alba* |
| 981 | Myricitrin | 464.38 | 3 | 12 | 8 | 2 | 0 | 1 | 1 | 3 | *Morus alba* |
| 982 | Oxyresveratrol | 244.24 | 2 | 4 | 4 | 0 | 0 | 0 | 0 | 0 | *Morus alba* |
| 983 | P-Cresol | 108.14 | 0 | 1 | 1 | 0 | 3 | 0 | 0 | 2 | *Morus alba* |
| 984 | Moracin M | 242.23 | 1 | 4 | 3 | 0 | 0 | 0 | 0 | 0 | *Morus alba* |
| 985 | Quercetin Dihydrate | 338.27 | 1 | 9 | 7 | 1 | 0 | 1 | 1 | 1 | *Morus alba* |
| 986 | Moracin C | 310.34 | 3 | 4 | 3 | 0 | 0 | 0 | 0 | 0 | *Morus alba* |
| 987 | Methyl Linolenate | 292.46 | 14 | 2 | 0 | 1 | 1 | 1 | 0 | 1 | *Morus alba* |
| 988 | Naphthalene | 128.17 | 0 | 0 | 0 | 1 | 2 | 0 | 0 | 2 | *Morus alba* |
| 989 | Esculin | 340.28 | 3 | 9 | 5 | 0 | 1 | 1 |  |  | *Morus alba* |
| 990 | 3-Hydroxycoumarin | 162.14 | 0 | 3 | 1 | 0 | 1 | 0 | 0 | 1 | *Morus alba* |
| 991 | Benzyl Alcohol | 108.14 | 1 | 1 | 1 | 0 | 3 | 0 | 0 | 2 | *Morus alba* |
| 992 | Fagomine | 147..17 | 1 | 4 | 4 | 0 | 3 | 0 | 0 | 1 | *Morus alba* |
| 993 | 4-Hydroxyacetophenone | 136.15 | 1 | 2 | 1 | 0 | 3 | 0 | 0 | 2 | *Morus alba* |
| 994 | Cudraflavone B | 420.45 | 3 | 6 | 3 | 0 | 0 | 0 | 0 | 1 | *Morus alba* |
| 995 | Gentisic Acid | 154.12 | 1 | 4 | 3 | 0 | 3 | 0 | 0 | 1 | *Morus alba* |
| 996 | Methyl (9Z,12Z)-Octadeca-9,12-Dienoate | 294.47 | 15 | 2 | 0 | 1 | 1 | 1 | 1 | 1 | *Morus alba* |
| 997 | 6-Hydroxycoumarin | 162.14 | 0 | 3 | 1 | 0 | 1 | 0 | 0 | 1 | *Morus alba* |
| 998 | 3-Epifagomine | 147.17 | 1 | 4 | 4 | 0 | 3 | 0 | 0 | 1 | *Morus alba* |
| 999 | Metacresol | 108.14 | 0 | 1 | 1 | 0 | 3 | 0 | 0 | 2 | *Morus alba* |
| 1000 | Paeonol | 166.17 | 2 | 3 | 1 | 0 | 0 | 0 | 0 | 1 | *Morus alba* |
| 1001 | Moracin P | 326.34 | 1 | 5 | 3 | 0 | 0 | 0 | 0 | 0 | *Morus alba* |
| 1002 | L-1-Deoxynojirimycin | 163.17 | 1 | 5 | 5 | 0 | 1 | 0 | 0 | 1 | *Morus alba* |
| 1003 | Loliolide | 196.24 | 0 | 3 | 1 | 0 | 0 | 0 | 0 | 1 | *Morus alba* |
| 1004 | Methyl Oleate | 296.49 | 16 | 2 | 0 | 1 | 1 | 1 | 1 | 2 | *Morus alba* |
| 1005 | [5,7-Dihydroxy-2-(3,4,5-Trihydroxyphenyl)-3,4-Dihydro-2H-Chromen-3-Yl] 3,4,5-Trihydroxybenzoate | 458.37 | 4 | 11 | 8 | 2 | 0 | 1 | 1 | 3 | *Morus alba* |
| 1006 | Glabrone | 336.34 | 1 | 5 | 2 | 0 | 0 | 0 | 0 | 0 | *Morus alba* |
| 1007 | Morusinol | 438.47 | 4 | 7 | 4 | 0 | 0 | 0 | 0 | 0 | *Morus alba* |
| 1008 | Moscatin | 240.25 | 0 | 0 | 0 | 0 | 0 | 0 | 0 | 0 | *Morus alba* |
| 1009 | Deoxyfuconojirimycin | 147.17 | 0 | 4 | 4 | 0 | 3 | 0 | 0 | 1 | *Morus alba* |
| 1010 | Norartocarpetin | 286.24 | 1 | 6 | 4 | 0 | 0 | 0 | 0 | 0 | *Morus alba* |
| 1011 | Epitaxifolin | 304.25 | 1 | 7 | 5 | 0 | 0 | 0 | 0 | 0 | *Morus alba* |
| 1012 | Iminoribitol | 133.15 | 1 | 4 | 4 | 0 | 3 | 0 | 0 | 1 | *Morus alba* |
| 1013 | N-Methyl-1-Deoxynojirimycin | 177.2 | 1 | 5 | 4 | 0 | 1 | 0 | 0 | 1 | *Morus alba* |
| 1014 | Kuwanon C | 422.47 | 5 | 6 | 4 | 0 | 0 | 0 | 0 | 1 | *Morus alba* |
| 1015 | Valeric Acid | 102.13 | 3 | 2 | 1 | 0 | 3 | 0 | 0 | 1 | *Morus alba* |
| 1016 | Atalantoflavone | 336.34 | 1 | 5 | 2 | 0 | 0 | 0 | 0 | 0 | *Morus alba* |
| 1017 | Moracin D | 308.33 | 1 | 4 | 2 | 0 | 0 | 0 | 0 | 0 | *Morus alba* |
| 1018 | Chlorogenic Acid Methyl Ester | 368.34 | 6 | 9 | 6 | 0 | 1 | 1 | 1 | 1 | *Morus alba* |
| 1019 | Cyclomorusin | 418.44 | 1 | 6 | 2 | 0 | 0 | 0 | 0 | 1 | *Morus alba* |
| 1020 | (2R,3S)-2-(3,4-Dihydroxyphenyl)-3,5,7-Trihydroxy-2,3-Dihydrochromen-4-One | 304.25 | 1 | 7 | 5 | 0 | 0 | 0 | 0 | 0 | *Morus alba* |
| 1021 | Moracin N | 310.34 | 3 | 4 | 3 | 0 | 0 | 0 | 0 | 0 | *Morus alba* |
| 1022 | Calystegine B2 | 175.18 | 0 | 5 | 5 | 0 | 1 | 0 | 0 | 2 | *Morus alba* |
| 1023 | Sanggenon C | 708.71 | 6 | 12 | 8 | 3 | 4 | 1 | 2 | 5 | *Morus alba* |
| 1024 | Morachalcone A | 340.37 | 5 | 5 | 4 | 0 | 0 | 0 | 0 | 0 | *Morus alba* |
| 1025 | 5-(6-Hydroxy-1-Benzofuran-2-Yl)-2,2-Dimethylchromen-7-Ol | 308.33 | 1 | 4 | 1 | 0 | 0 | 0 | 0 | 0 | *Morus alba* |
| 1026 | Isobutyric Acid | 88.11 | 1 | 2 | 1 | 0 | 3 | 0 | 0 | 2 | *Morus alba* |
| 1027 | Trigonelline Hydrochloride | 173.6 | 1 | 2 | 1 | 0 | 2 | 0 | 0 | 1 | *Morus alba* |
| 1028 | Moracin O | 326.34 | 2 | 5 | 3 | 0 | 0 | 0 | 0 | 0 | *Morus alba* |
| 1029 | Ethyl Benzoate | 150.17 | 3 | 2 | 0 | 0 | 1 | 0 | 0 | 1 | *Morus alba* |
| 1030 | Kuwanon E | 424.49 | 6 | 6 | 4 | 0 | 0 | 0 | 0 | 1 | *Morus alba* |
| 1031 | 5-(4-Methoxy-8-Methyl-7,10-Dihydrofuro[2,3-G][1]Benzoxepin-2-Yl)Benzene-1,3-Diol | 338.35 | 2 | 5 | 2 | 0 | 0 | 0 | 0 | 0 | *Morus alba* |
| 1032 | Benzyl Beta-D-Glucopyranoside | 270.28 | 4 | 6 | 4 | 0 | 1 | 0 | 0 | 0 | *Morus alba* |
| 1033 | Moracin X | 266.25 | 1 | 4 | 2 | 0 | 0 | 0 | 0 | 0 | *Morus alba* |
| 1034 | 5-(8-Methyl-7,10-Dihydrofuro[2,3-G][1]Benzoxepin-2-Yl)Benzene-1,3-Diol | 308.33 | 1 | 4 | 2 | 0 | 0 | 0 | 0 | 0 | *Morus alba* |
| 1035 | Hexadecanoic Acid Methyl Ester | 270.45 | 15 | 2 | 0 | 1 | 1 | 1 | 0 | 1 | *Morus alba* |
| 1036 | Sanggenon D | 708.71 | 6 | 12 | 8 | 3 | 4 | 1 | 2 | 5 | *Morus alba* |
| 1037 | Cudraflavone A | 414.44 | 1 | 6 | 2 | 0 | 0 | 0 | 0 | 1 | *Morus alba* |
| 1038 | Steppogenin | 288.25 | 1 | 6 | 4 | 0 | 0 | 0 | 0 | 0 | *Morus alba* |
| 1039 | Albafuran A | 378.46 | 6 | 4 | 3 | 0 | 1 | 0 | 1 | 1 | *Morus alba* |
| 1040 | 5,7-Dihydroxycoumarin | 178.14 | 0 | 4 | 2 | 0 | 1 | 0 | 0 | 1 | *Morus alba* |
| 1041 | Kuwanon V | 646.72 | 9 | 8 | 8 | 2 | 4 | 1 | 2 | 4 | *Morus alba* |
| 1042 | Liriodendrtachioside | 646.72 | 9 | 8 | 6 | 2 | 4 | 1 | 2 | 4 | *Morus alba* |
| 1043 | Kuwanon J | 678.72 | 9 | 10 | 8 | 2 | 4 | 1 | 2 | 4 | *Morus alba* |
| 1044 | Sanggenon O | 708.71 | 6 | 12 | 8 | 3 | 4 | 1 | 2 | 5 | *Morus alba* |
| 1045 | But-2-Enoic Acid | 86.09 | 1 | 2 | 1 | 0 | 3 | 0 | 0 | 2 | *Morus alba* |
| 1046 | Mulberrofuran G | 155.57 | 2 | 8 | 5 | 1 | 3 | 0 | 2 | 2 | *Morus alba* |
| 1047 | Kumanon G | 692.71 | 7 | 11 | 8 | 3 | 4 | 1 | 2 | 5 | *Morus alba* |
| 1048 | Cudraflavone C | 422.47 | 5 | 6 | 4 | 0 | 0 | 0 | 0 | 1 | *Morus alba* |
| 1049 | Squalane | 422.81 | 21 | 0 | 0 | 1 | 3 | 1 | 1 | 3 | *Morus alba* |
| 1050 | Dihydromorin | 304.25 | 1 | 7 | 5 | 0 | 0 | 0 | 0 | 0 | *Morus alba* |
| 1051 | Dihydroferulic Acid | 196.2 | 4 | 4 | 2 | 0 | 0 | 0 | 0 | 1 | *Morus alba* |
| 1052 | (2R)-2-(3,4-Dihydroxyphenyl)-3,5,7-Trihydroxy-2,3-Dihydrochromen-4-One | 304.25 | 1 | 7 | 5 | 0 | 0 | 0 | 0 | 0 | *Morus alba* |
| 1053 | Mulberrofuran Y | 408.49 | 7 | 5 | 3 | 0 | 1 | 0 | 1 | 1 | *Morus alba* |
| 1054 | Dihydroguaiareticacid | 330.42 | 7 | 4 | 2 | 0 | 0 | 0 | 0 | 0 | *Paederia foetida* |
| 1055 | Licarine A | 330.42 | 7 | 4 | 2 | 0 | 0 | 0 | 0 | 0 | *Paederia foetida* |
| 1056 | Acuminatin | 326.39 | 4 | 4 | 1 | 0 | 0 | 0 | 0 | 0 | *Paederia foetida* |
| 1057 | Oleiferin C | 342.39 | 5 | 5 | 1 | 0 | 0 | 0 | 0 | 0 | *Paederia foetida* |
| 1058 | Gallic Acid Methyl Ester | 184.15 | 2 | 5 | 3 | 0 | 0 | 0 | 0 | 1 | *Phyllanthus emblica* |
| 1059 | Dihydrokaempferol | 288.25 | 1 | 6 | 4 | 0 | 0 | 0 | 0 | 0 | *Phyllanthus emblica* |
| 1060 | Putrescine Hydrochloride | 161.07 | 3 | 2 | 2 | 0 | 0 | 0 | 0 | 1 | *Phyllanthus emblica* |
| 1061 | 3-Deoxysappanchalcone | 270.28 | 4 | 4 | 2 | 0 | 0 | 0 | 0 | 0 | *Phyllanthus emblica* |
| 1062 | 7-Trimethylkaempferol | 328.32 | 4 | 6 | 1 | 0 | 0 | 0 | 0 | 0 | *Phyllanthus emblica* |
| 1063 | 1-Octen-3-Ol | 128.21 | 5 | 1 | 1 | 0 | 1 | 0 | 0 | 2 | *Phyllanthus emblica* |
| 1064 | Rhamnocitrin | 300.26 | 2 | 6 | 3 | 0 | 0 | 0 | 0 | 0 | *Phyllanthus emblica* |
| 1065 | Nonanol | 144.25 | 7 | 1 | 1 | 0 | 1 | 0 | 0 | 2 | *Phyllanthus emblica* |
| 1066 | 2-(4-Hydroxyphenyl)-3,4-Dihydro-2H-Chromene-3,5,7-Triol | 274.27 | 1 | 5 | 4 | 0 | 0 | 0 | 0 | 0 | *Phyllanthus emblica* |
| 1067 | (2R)-2-Hydroxybutanedioic Acid | 134.09 | 3 | 5 | 3 | 0 | 4 | 0 | 0 | 2 | *Phyllanthus emblica* |
| 1068 | 1-Eicosanol | 298.55 | 18 | 1 | 1 | 1 | 1 | 1 | 1 | 3 | *Phyllanthus emblica* |
| 1069 | Phyllaemblic acid methy ester | 434.44 | 5 | 9 | 2 | 0 | 0 | 0 | 0 | 0 | *Phyllanthus emblica* |
| 1070 | 1-Heptadecanol | 256.47 | 15 | 1 | 1 | 1 | 1 | 1 | 0 | 2 | *Phyllanthus emblica* |
| 1071 | Oct-1-En-3-Ol | 128.21 | 5 | 1 | 1 | 0 | 1 | 0 | 0 | 2 | *Phyllanthus emblica* |
| 1072 | Digiferruginol | 254.24 | 1 | 4 | 2 | 0 | 0 | 0 | 0 | 0 | *Phyllanthus emblica* |
| 1073 | Methyl Protocatechuate | 168.15 | 2 | 4 | 2 | 0 | 0 | 0 | 0 | 1 | *Schleichera oleosa* |
| 1074 | Schleicheol 1 | 444.73 | 7 | 2 | 1 | 1 | 3 | 0 | 1 | 1 | *Schleichera oleosa* |
| 1075 | Schleicherastatin 1 | 460.73 | 7 | 3 | 2 | 1 | 3 | 0 | 1 | 1 | *Schleichera oleosa* |
| 1076 | Schleicherastatin 3 | 446.71 | 6 | 3 | 2 | 1 | 3 | 0 | 1 | 1 | *Schleichera oleosa* |
| 1077 | Schleicherastatin 5 | 444.69 | 6 | 3 | 2 | 1 | 3 | 0 | 1 | 1 | *Schleichera oleosa* |
| 1078 | Schleicherastatin 6 | 430.66 | 5 | 3 | 2 | 1 | 2 | 0 | 0 | 1 | *Schleichera oleosa* |
| 1079 | Ethyl Vanillin | 166.17 | 3 | 3 | 1 | 0 | 0 | 0 | 0 | 1 | *Sesbania grandiflora* |
| 1080 | 1-Methylpyrrolidin-2-One | 99.13 | 0 | 1 | 0 | 0 | 3 | 0 | 0 | 1 | *Sesbania grandiflora* |
| 1081 | Syringaldehyde | 182.17 | 3 | 4 | 1 | 0 | 0 | 0 | 0 | 1 | *Sesbania grandiflora* |
| 1082 | Sakuranetin | 286.28 | 2 | 5 | 2 | 0 | 0 | 0 | 0 | 0 | *Sesbania grandiflora* |
| 1083 | Evofolin B | 318.32 | 6 | 6 | 3 | 0 | 0 | 0 | 0 | 0 | *Sesbania grandiflora* |
| 1084 | Mutangin | 636.69 | 13 | 11 | 0 | 2 | 3 | 2 | 1 | 2 | *Sesbania grandiflora* |
| 1085 | 1,3,5-Trihydroxy-8-[(2R,3S,4R,5R,6S)-3,4,5-Trihydroxy-6-(Hydroxymethyl)Oxan-2-Yl]Oxyxanthen-9-One | 422.34 | 3 | 11 | 7 | 2 | 1 | 1 | 1 | 3 | *Swertia perennis* |
| 1086 | (2E,4E,6R)-7-[4-(Dimethylamino)Phenyl]-4,6-Dimethyl-7-Oxo-N-[(2R,3R,4S,5S,6R)-3,4,5-Trihydroxy-6-(Hydroxymethyl)Oxan-2-Yl]Oxyhepta-2,4-Dienamide | 464.51 | 10 | 8 | 5 | 0 | 0 | 1 | 1 | 0 | *Swertia perennis* |
| 1087 | Swertianolin | 436.37 | 4 | 11 | 6 | 2 | 1 | 1 | 1 | 3 | *Swertia perennis* |
| 1088 | Decussatin | 302.28 | 3 | 6 | 1 | 0 | 0 | 0 | 0 | 0 | *Swertia perennis* |
| 1089 | 1,5-Dihydroxy-3-Methoxy-8-[(2R,3S,4R,5R,6S)-3,4,5-Trihydroxy-6-(Hydroxymethyl)Oxan-2-Yl]Oxyxanthen-9-One | 436.37 | 4 | 11 | 6 | 2 | 1 | 1 | 1 | 3 | *Swertia perennis* |
| 1090 | 1,2,6,8-Tetrahydroxyxanthen-9-One | 260.2 | 0 | 6 | 4 | 0 | 0 | 0 | 0 | 0 | *Swertia perennis* |
| 1091 | Deacetylgedunin | 440.53 | 1 | 6 | 1 | 0 | 0 | 0 | 0 | 0 | *Swietenia macrophylla* |
| 1092 | Swietenolide | 486.55 | 4 | 8 | 2 | 0 | 1 | 0 | 0 | 0 | *Swietenia macrophylla* |
| 1093 | Methylangolensate | 470.55 | 4 | 7 | 0 | 0 | 0 | 0 | 0 | 0 | *Swietenia macrophylla* |
| 1094 | Methyl (1S,4As,5As,6S,10As)-1-Methyl-2'-Oxospiro[1,4A,5,5A,7,8,10,10A-Octahydropyrano[3,4-F]Indolizine-6,3'-1H-Indole]-4-Carboxylate | 368.43 | 2 | 5 | 1 | 0 | 0 | 0 | 0 | 0 | *Swietenia macrophylla* |
| 1095 | Pinocembrin | 256.25 | 1 | 4 | 2 | 0 | 0 | 0 | 0 | 0 | *Syzygium cumini* |
| 1096 | 2-[[4-[(2-Amino-4-Oxo-1H-Pteridin-6-Yl) Methylamino] Benzoyl] Amino]Pentanedioic Acid | 441.4 | 10 | 9 | 6 | 2 | 0 | 1 | 1 | 2 | *Syzygium cumini* |
| 1097 | Sandaracopimaric Acid | 302.45 | 2 | 2 | 1 | 1 | 0 | 0 | 0 | 1 | *Syzygium cumini* |
| 1098 | 1R-(+)-Alpha-Pinene | 136.23 | 0 | 0 | 0 | 1 | 1 | 0 | 0 | 2 | *Syzygium cumini* |
| 1099 | (2R)-6-Methyl-2-[(1R)-4-Methylcyclohex-3-En-1-Yl] Hept-5-En-2-Ol | 222.37 | 4 | 1 | 1 | 0 | 0 | 0 | 0 | 1 | *Syzygium cumini* |
| 1100 | Sandaracopimaradienediol | 304.47 | 2 | 2 | 2 | 0 | 0 | 0 | 0 | 0 | *Syzygium cumini* |
