# Supplementary material for: Computational and immunoinformatics approaches for designing phytocompound-based drugs and a multi-epitope vaccine targeting FemA, a cell wall protein of Staphylococcus aureus
Source: PLoS One. 2026 Apr 7;21(4):e0346271. doi: 10.1371/journal.pone.0346271 (PMC13056209; doi:10.1371/journal.pone.0346271)
Supplement: S2 Table — (DOCX) [file pone.0346271.s002.docx]

**S2 Table. ADMET properties of the selected phytocompounds.**

| **SL** | **Phytocompounds** | **Water solubility** | **Caco2 permeability** | **Human intestinal absorption (% absorbed)** | **P-glycoprotein substrate** | **P-glycoprotein I inhibitor** | **P-glycoprotein II inhibitor** | **BBB permeability** | **CYP2D6 substrate** | **CYP3A4 substrate** | **CYP1A2 inhibitor** | **CYP2C19 inhibitior** | **CYP2C9 inhibitior** | **CYP2D6 inhibitior** | **CYP3A4 inhibitior** | **AMES toxicity** | **Max. tolerated dose (human)** | **hERG I inhibitor** | **hERG II inhibitor** | **Hepatotoxicity** |
| --- | --- | --- | --- | --- | --- | --- | --- | --- | --- | --- | --- | --- | --- | --- | --- | --- | --- | --- | --- | --- |
| 1 | Nimbinin | -4.3 | 0.906 | 98.649 | No | Yes | No | -0.431 | No | Yes | No | No | No | No | Yes | No | -0.658 | No | No | No |
| 2 | Azadiradione | -5.105 | 0.852 | 98.698 | No | Yes | Yes | -0.194 | No | Yes | No | No | No | No | Yes | No | -0.224 | No | Yes | No |
| 3 | Vasicolinone | -4.362 | 1.71 | 98.734 | No | Yes | Yes | 0.568 | No | Yes | Yes | Yes | No | No | No | No | -0.164 | No | No | Yes |
| 4 | Nimbinone | -3.233 | 1.308 | 93.587 | Yes | No | No | -0.2 | No | No | Yes | Yes | No | No | No | No | -0.683 | No | No | No |
| 5 | 6-Deacetylnimbinene | -4.733 | 1.31 | 97.591 | No | Yes | Yes | -0.347 | No | Yes | No | No | No | No | Yes | No | -0.511 | No | No | Yes |
| 6 | Anisotine | -4.082 | 0.906 | 94.97 | Yes | Yes | Yes | -0.381 | No | Yes | Yes | Yes | Yes | No | Yes | No | 0.158 | No | No | Yes |
| 7 | Aloe-emodin | -3.104 | -0.233 | 74.179 | Yes | No | No | -0.729 | No | No | Yes | No | No | No | No | Yes | -0.089 | No | No | No |
| 8 | Nimbandiol | -4.882 | 0.823 | 93.361 | Yes | Yes | No | -0.643 | No | Yes | No | No | No | No | Yes | No | -0.604 | No | No | No |
| 9 | 1,8-Dihydroxyanthracene | -4.276 | 1.523 | 92.882 | Yes | No | No | 0.22 | No | Yes | Yes | Yes | Yes | No | No | Yes | 0.257 | No | No | No |
| 10 | 1,3-Dihydroxy-6,7-dimethoxyxanthone | -3.229 | 1.234 | 95.48 | Yes | No | No | -0.414 | No | Yes | Yes | Yes | No | No | No | Yes | 0.044 | No | No | No |
| 11 | Capsaicin | -4.185 | 1.364 | 90.075 | Yes | No | No | -0.241 | No | Yes | Yes | Yes | Yes | No | No | No | 0.46 | No | Yes | Yes |
| 12 | Vasicinol | -2.537 | 1.137 | 80.398 | No | No | No | -0.294 | No | No | No | No | No | No | No | No | -0.391 | No | No | No |
| 13 | Cis-linalool-oxide | -1.968 | 1.642 | 97.294 | No | No | No |  | No | No | No | No | No | No | No | No | 0.539 | No | No | No |
| 14 | Vasicoline | -4.848 | -4.848 | 93.064 | No | No | No | 0.614 | Yes | Yes | Yes | Yes | Yes | Yes | No | Yes | 0.088 | No | Yes | Yes |
| 15 | Vasicol | -2.002 | 0.077 | 65.276 | No | No | No | -0.232 | No | No | No | No | No | No | No | No | 0.039 | No | No | No |
| 16 | N-heptane | -3.639 | 1.383 | 94.145 | No | No | No | 0.77 | No | No | No | No | No | No | No | No | 0.749 | No | No | No |
| 17 | Thiamine | -2.794 | 0.867 | 100 | Yes | No | No | -0.368 | No | No | Yes | No | No | No | No | No | -0.251 | No | No | Yes |
| 18 | Citronellyl isobutyrate | -4.344 | 1.541 | 95.948 | No | No | No | 0.625 | No | No | No | No | No | No | No | No | 0.691 | No | No | No |
| 19 | Niazimin A | -2.881 | 0.505 | 58.312 | Yes | No | No | -1.092 | No | No | No | No | No | No | No | No | 0.361 | No | No | Yes |
| 20 | Apigenin | -3.329 | 1.007 | 93.25 | Yes | No | No | -0.734 | No | No | Yes | Yes | No | No | No | No | 0.328 | No | No | No |
| 21 | 7-Hydroxy-chromone | -3.647 | 1.054 | 93.455 | Yes | Yes | Yes | -0.103 | No | Yes | Yes | Yes | No | Yes | No | No | -0.33 | Yes | Yes | Yes |
| 22 | Aloechrysone | -3.286 | 1.131 | 94.802 | Yes | No | No | 0.201 | No | No | Yes | Yes | No | No | No | No |  | No | No | No |
| 23 | Vasicinone | -2.075 | 1.172 | 92.532 | Yes | No | No | -0.206 | No | No | Yes | No | No | No | No | No | 0.332 | No | No | No |
| 24 | Vasicinolone | -2.577 | 0.083 | 98.31 | Yes | No | No | 0.212 | No | No | Yes | No | No | No | No | No | -0.244 | No | No | No |
| 25 | Quercetin | -2.925 | -0.229 | 77.207 | Yes | No | No | -1.098 | No | No | Yes | No | No | No | No | No | 0.499 | No | No | No |
| 26 | 17-Hydroxyazadiradione | -4.186 | 1.197 | 100 | No | Yes | Yes | -0.302 | No | Yes | No | No | No | No | Yes | No | -1.072 | No | No | No |
| 27 | Epoxyazadiradione | -4.3 | 0.906 | 98.649 | No | Yes | No | -0.431 | No | Yes | No | No | No | No | Yes | No | -0.658 | No | No | No |
| 28 | Nimbolide | -5.166 | 0.92 | 100 | No | Yes | Yes | -0.675 | No | Yes | No | No | No | No | No | No | -0.476 | No | No | No |
| 29 | 7-Acetyl-16,17-Dehydro-16-Hydroxyneotrichilenone | -4.264 | 1.059 | 100 | No | Yes | Yes | -0.364 | No | Yes | No | No | No | No | Yes | No | -0.899 | No | No | Yes |
| 30 | Beta-Nimolactone | -4.618 | 1.108 | 97.415 | No | Yes | No | -0.231 | No | Yes | No | No | No | No | No | No | -0.57 | No | No | No |
| 31 | Alpha-Nimolactone | -4.618 | 1.108 | 97.415 | No | Yes | No | -0.231 | No | Yes | No | No | No | No | No | No | -0.57 | No | No | No |
| 32 | 17-Epi-17-Hydroxyazadiradione | -4.186 | 1.197 | 100 | No | Yes | Yes | -0.302 | No | Yes | No | No | No | No | Yes | No | -1.072 | No | No | No |
| 33 | Desfuranoazadiradione | -4.728 | 1.352 | 98.361 | No | Yes | Yes | -0.355 | No | Yes | No | No | No | No | No | No | -0.472 | No | Yes | No |
| 34 | 15-Hydroxyazadiradione | -4.306 | 1.127 | 100 | No | Yes | Yes | -0.325 | No | Yes | No | No | No | No | Yes | No | -0.876 | No | No | Yes |
| 35 | Ethinyl Estradiol | -3.836 | 1.616 | 94.884 | Yes | No | No | -0.024 | No | Yes | Yes | Yes | No | No | No | No | -0.446 | No | Yes | No |
| 36 | Genistein | -3.595 | 0.9 | 93.387 | Yes | No | No | -0.71 | No | No | Yes | Yes | No | No | No | No | 0.478 | No | No | No |
| 37 | Kaempferol | -3.04 | 0.032 | 74.29 | Yes | No | No | -0.939 | No | No | Yes | No | No | No | No | No | 0.531 | No | No | No |
| 38 | Paraben | -3.228 | 0.821 | 93.522 | Yes | No | No | -0.626 | No | No | No | No | No | No | No | No | 0.399 | No | No | No |
| 39 | Ajoene | -3.54 | 1.329 | 95.186 | No | No | No | 0.703 | No | No | No | No | No | No | No | No | 0.462 | No | No | No |
| 40 | Sinapinate | -2.869 | 0.272 | 93.064 | Yes | No | No | -0.247 | No | No | No | No | No | No | No | No | 1.193 | No | No | No |
| 41 | Biotin | -2 | 0.698 | 71.182 | No | No | No | -0.679 | No | No | No | No | No | No | No | No | 0.11 | No | No | Yes |
| 42 | Pyridoxal Phosphate | -1.705 | -0.132 | 66.135 | No | No | No | -1.282 | No | No | No | No | No | No | No | No | 0.423 | No | No | No |
| 43 | Lariciresinol | -3.984 | 1.035 | 91.656 | Yes | No | No | -0.834 | No | Yes | No | Yes | No | No | Yes | No | -0.099 | No | Yes | No |
| 44 | Matairesinol | -3.685 | 1.115 | 93.527 | Yes | Yes | Yes | -0.49 | No | Yes | Yes | Yes | Yes | No | Yes | No | -0.164 | No | Yes | No |
| 45 | Pyridoxine Hydrochloride | -2.264 | 0.571 | 97.949 | No | No | No | -1.029 | No | No | No | No | No | No | No | No | -0.197 | No | No | No |
| 46 | Isoeugenitol | -2.725 | 1.002 | 93.126 | Yes | No | No | -0.048 | No | No | Yes | No | No | No | No | No | 0.241 | No | No | No |
| 47 | Isoflavone | -4.351 | 1.767 | 96.388 | Yes | No | Yes | 0.16 | No | Yes | Yes | Yes | Yes | No | No | No | 0.107 | No | No | No |
| 48 | Naringenin | -3.224 | 1.029 | 91.31 | Yes | No | No | -0.578 | No | No | Yes | No | No | No | No | No | -0.176 | No | No | No |
| 49 | Fisetin | -3.181 | 0.058 | 83.752 | Yes | No | No | -1.039 | No | No | Yes | No | Yes | No | No | No | 0.579 | No | No | No |
| 50 | Cianidanol | -3.117 | -0.283 | 68.829 | Yes | No | No | -1.054 | No | No | No | No | No | No | No | No | 0.438 | No | No | No |
| 51 | Morin | -2.978 | -0.294 | 75.408 | Yes | No | No | -1.18 | No | No | Yes | No | No | No | No | No | 0.537 | No | No | No |
| 52 | 6-Paradol | -4.083 | 1.401 | 92.18 | No | Yes | No | -0.223 | No | Yes | Yes | Yes | Yes | No | No | No | 0.819 | No | Yes | No |
| 53 | Diethyl Phthalate | -2.428 | 1.306 | 96.593 | No | No | No | 0.138 | No | No | Yes | No | No | No | No | No | 1.37 | No | No | No |
| 54 | Pinostrobin | -3.445 | 1.296 | 93.762 | No | No | No | 0.085 | No | Yes | Yes | Yes | No | No | No | Yes | 0.26 | No | No | No |
| 55 | Eugenyl Acetate | -2.846 | 1.659 | 94.755 | No | No | No | 0.401 | No | No | Yes | No | No | No | No | No | 1.102 | No | No | No |
| 56 | Aframodial | -4.984 | 1.398 | 97.783 | No | Yes | No | -0.197 | No | Yes | No | Yes | No | No | No | No | -0.675 | No | No | No |
| 57 | Santamarin | -2.954 | 1.282 | 96.824 | No | No | No | 0.083 | No | No | Yes | No | No | No | No | No | 0.283 | No | No | No |
| 58 | 3'-Hydroxy-4'-methoxyglabridin | -4.178 | 0.875 | 93.965 | Yes | Yes | No | -0.234 | No | Yes | Yes | Yes | Yes | No | Yes | No | 0.045 | No | Yes | No |
| 59 | Hexahydrocurcumin | -3.88 | -0.094 | 72.703 | Yes | Yes | Yes | -0.964 | No | Yes | No | Yes | No | No | Yes | No | 0.243 | No | No | No |
| 60 | 6-Dehydrogingerdione | -3.48 | 0.913 | 93.152 | No | Yes | No | -0.318 | No | Yes | Yes | Yes | No | No | No | No | 0.662 | No | No | No |
| 61 | Cubebin | -4.516 | 1.236 | 97.749 | Yes | Yes | No | -1.111 | No | Yes | No | Yes | No | No | Yes | Yes | -0.273 | No | Yes | No |
| 62 | Paravallarine | -3.9 | 1.168 | 94.132 | No | Yes | No | 0.282 | No | Yes | No | No | No | Yes | No | No | -0.237 | No | No | Yes |
| 63 | Papaverine | -5.118 | 1.414 | 95.909 | No | Yes | Yes | -0.348 | No | Yes | Yes | Yes | No | No | No | No | 0.682 | No | Yes | Yes |
| 64 | Farrerol | -3.327 | 1.22 | 91.927 | Yes | No | No | -0.777 | No | No | Yes | Yes | Yes | No | No | No | -0.207 | No | No | No |
| 65 | Diosmetin | -3.238 | 0.326 | 79.898 | Yes | No | No | -0.954 | No | No | Yes | Yes | Yes | No | No | No | 0.42 | No | No | No |
| 66 | Isoimperatorin | -3.754 | 1.343 | 97.745 | No | No | No | 0.149 | No | Yes | Yes | Yes | Yes | Yes | Yes | Yes | -0.317 | No | No | No |
| 67 | Elemicin | -2.665 | 1.859 | 95.971 | No | No | No | 0.368 | No | No | Yes | No | No | No | No | Yes | 1.233 | No | No | No |
| 68 | Apiole | -2.355 | 1.837 | 95.196 | No | No | No | -0.513 | No | Yes | Yes | No | No | No | No | Yes | 0.561 | No | No | No |
| 69 | Cnidilin | -3.955 | 1.37 | 97.79 | No | No | No | 0.07 | No | Yes | Yes | Yes | Yes | No | No | No | -0.265 | No | No | No |
| 70 | Hydrocortisone | -3.709 | 0.411 | 75.265 | Yes | No | No | -0.48 | No | No | No | No | No | No | No | No | -0.183 | No | No | No |
| 71 | Rhein | -2.843 | -0.241 | 55 | Yes | No | No | -0.807 | No | No | No | No | No | No | No | No | 0.716 | No | No | No |
| 72 | Galangin | -3.34 | 0.999 | 93.985 | Yes | No | No | -0.748 | No | No | Yes | Yes | Yes | No | No | No | 0.333 | No | No | No |
| 73 | Nataloe-Emodin | -3.192 | 0.055 | 74.485 | Yes | No | No | -0.727 | No | No | Yes | No | No | No | No | No | 0.158 | No | No | No |
| 74 | Madagascin | -3.702 | 1.514 | 95.769 | Yes | No | No | 0.039 | No | Yes | Yes | Yes | Yes | No | No | No | -0.27 | No | No | No |
| 75 | Cheilanthifoline | -3.373 | 1.236 | 93.903 | Yes | Yes | No | 0.665 | No | Yes | Yes | No | No | Yes | No | No | -0.343 | No | Yes | No |
| 76 | Rhein | -2.843 | -0.241 | 55 | Yes | No | No | -0.807 | No | No | No | No | No | No | No | No | 0.716 | No | No | No |
| 77 | Physcion | -3.143 | 1.438 | 96.356 | Yes | No | No | -0.023 | No | No | No | No | No | No | No | Yes | -0.256 | No | No | No |
| 78 | Chrysophanol | -3.077 | 1.298 | 96.558 | Yes | No | No | 0.212 | No | No | Yes | No | No | No | No | Yes | -0.256 | No | No | No |
| 79 | Isorhamnetin | -3 | -0.003 | 76.014 | Yes | No | No | -1.135 | No | No | Yes | No | No | No | No | No | 0.576 | No | No | No |
| 80 | Ayanin | -3.48 | 1.374 | 91.735 | Yes | No | Yes | -0.841 | No | Yes | Yes | Yes | No | No | Yes | No | 0.457 | No | No | No |
| 81 | 7-Hydroxy-2-(4-Hydroxyphenyl)Chromen-4-One | -3.588 | 1.037 | 93.952 | Yes | No | No | -0.05 | No | No | Yes | Yes | Yes | No | No | No | 0.175 | No | No | No |
| 82 | Androstenedione | -4.799 | 1.575 | 98.492 | No | Yes | Yes | 0.231 | No | Yes | No | No | No | No | No | No | -0.444 | No | Yes | No |
| 83 | Pinosylvin | -3.286 | 1.708 | 90.842 | Yes | No | No | 0.391 | No | Yes | Yes | Yes | Yes | No | No | No | 0.6 | No | No | No |
| 84 | Isolariciresinol | -3.84 | -0.049 | 73.973 | Yes | No | No | -0.915 | No | Yes | No | Yes | No | No | No | No | 0.134 | Yes | No | No |
| 85 | 12-Hydroxydehydroabietic Acid | -3.694 | 1.336 | 95.854 | No | No | No | -0.339 | No | Yes | No | No | No | No | No | No | 0.027 | No | No | No |
| 86 | Pinifolic Acid | -2.818 | 0.494 | 99.843 | No | No | No | -0.108 | No | Yes | No | No | No | No | No | No | -0.004 | No | No | Yes |
| 87 | 7-Oxodehydroabietinol | -5.273 | 1.691 | 92.565 | No | No | No | -0.029 | No | Yes | No | Yes | No | No | No | No | -0.242 | No | No | No |
| 88 | Syringetin | -3.042 | -0.204 | 77.277 | Yes | No | No | -1.299 | No | No | Yes | No | No | No | No | No | 0.532 | No | No | No |
| 89 | 7Beta,18-Dihydroxydehydroabietanol | -4.533 | 1.645 | 91.524 | Yes | No | No | -0.043 | No | Yes | Yes | Yes | No | No | No | No | 0.152 | No | No | No |
| 90 | Xanthoxyletin | -3.744 | 1.273 | 97.064 | No | No | No | 0.17 | No | Yes | Yes | Yes | No | No | No | No | 0.16 | No | No | No |
| 91 | Sesamin | -4.223 | 1.399 | 97.81 | No | Yes | No | -0.862 | No | Yes | Yes | Yes | Yes | No | Yes | Yes | 0.089 | No | No | No |
| 92 | Lasiodiplodin | -3.32 | 1.349 | 91.456 | No | No | No | 0.307 | No | Yes | No | No | No | No | No | No | -0.156 | No | No | No |
| 93 | Sesalin | -3.041 | 1.128 | 97.946 | No | No | No | 0.272 | No | Yes | No | No | No | No | No | No | 0.033 | No | No | No |
| 94 | Dentatin | -4.962 | 1.373 | 96.22 | No | Yes | No | 0.111 | No | Yes | Yes | Yes | Yes | No | No | No | -0.104 | No | No | No |
| 95 | Nordentatin | -4.424 | 1.269 | 94.497 | Yes | No | No | -0.016 | No | No | Yes | Yes | Yes | No | No | No | -0.077 | No | No | No |
| 96 | Sesamolin | -4.158 | 1.412 | 98.021 | No | Yes | No | -0.49 | No | Yes | No | Yes | No | No | Yes | Yes | 0.459 | No | No | No |
| 97 | Niazirin | -2.237 | 0.48 | 58.333 | Yes | No | No | -0.65 | No | No | No | No | No | No | No | No | 0.533 | No | No | No |
| 98 | 1,3-Dibenzylurea | -3.37 | 1.699 | 91.224 | Yes | No | No | 0.32 | No | Yes | Yes | Yes | No | No | No | No | 0.596 | No | No | No |
| 99 | Luteolin | -3.094 | 0.096 | 81.13 | Yes | No | No | -0.907 | No | No | Yes | No | Yes | No | No | No | 0.499 | No | No | No |
| 100 | Diffractaic Acid | -3.287 | 0.976 | 64.266 | Yes | No | No | -0.464 | No | No | No | No | No | No | No | No | 1.066 | No | No | No |
| 101 | Dextrose | -1.492 | 0.623 | 100 | Yes | No | No | -3.065 | No | No | No | No | No | No | No | No | -0.162 | No | No | Yes |
| 102 | Peonidin | -3.508 | -0.133 | 89.163 | Yes | No | No | -2.297 | No | Yes | Yes | No | Yes | No | No | No | 0.568 | No | No | No |
| 103 | Desacetyl-Beta-Cyclopyrethrosin | -2.635 | 0.532 | 77.504 | No | No | No | -0.208 | No | No | No | No | No | No | No | No | 0.743 | No | No | No |
| 104 | Tatridin B | -2.391 | 0.618 | 96.841 | No | No | No | -0.252 | No | No | No | No | No | No | No | No | 0.663 | No | No | No |
| 105 | Sinugibberodiol | -2.922 | 1.612 | 93.726 | No | No | No | 0.358 | No | No | No | No | No | No | No | No | 0.862 | No | No | No |
| 106 | Elegalactone A | -2.635 | 0.532 | 77.504 | No | No | No | -3.324 | No | No | No | No | No | No | No | No | 0.743 | No | No | No |
| 107 | 1-Epitatridin B | -2.391 | 0.618 | 96.841 | No | No | No | -0.252 | No | No | No | No | No | No | No | No | 0.663 | No | No | No |
| 108 | Tamirin | -2.59 | 0.643 | 98.043 | No | No | No | -0.189 | No | No | No | No | No | No | No | No | 0.58 | No | No | No |
| 109 | Aegeline | -3.311 | 1.245 | 93.228 | Yes | No | No | -0.098 | No | Yes | Yes | Yes | No | No | No | Yes | -0.084 | No | Yes | No |
| 110 | Scoparone | -1.976 | 1.298 | 97.879 | No | No | No | -2.328 | No | No | Yes | No | No | No | No | No | 0.494 | No | No | No |
| 111 | Gamma-Fagarine | -2.967 | 1.352 | 98.555 | No | No | No | -1.782 | No | Yes | Yes | Yes | Yes | No | No | Yes | -0.049 | No | No | No |
| 112 | Marmesin | -3.316 | 1.155 | 96.484 | No | No | No | -2.824 | No | No | Yes | No | No | No | No | No | 0.272 | No | No | No |
| 113 | Anhydroaegeline | -4.577 | 1.424 | 91.91 | No | No | No | 0.265 | No | Yes | Yes | Yes | Yes | No | No | No | 0.586 | No | Yes | Yes |
| 114 | Rhamnetin | -3.312 | -0.361 | 80.214 | Yes | No | No | -1.345 | No | No | Yes | No | No | No | No | No | 0.56 | No | No | No |
| 115 | 1,3,7-Trihydroxyxanthone | -3.365 | 1.111 | 93.733 | Yes | No | No | -0.764 | No | No | Yes | No | No | No | No | Yes | 0.166 | No | No | No |
| 116 | 5-Hydroxy-1,3-Dimethoxyxanthone | -3.244 | 1.171 | 96.377 | No | No | Yes | -0.294 | No | Yes | Yes | Yes | Yes | No | No | Yes | 0.036 | No | No | No |
| 117 | Eudesmane-4Alpha,11-Diol | -3.322 | 1.593 | 93.151 | No | No | No | 0.116 | No | No | No | No | No | No | No | No | 0.266 | No | No | No |
| 118 | Chrysin | -3.538 | 0.945 | 93.761 | Yes | No | No | 0.047 | No | No | Yes | Yes | Yes | No | No | No | 0.016 | No | No | No |
| 119 | 3-O-Methylquercetin | -3.16 | -0.615 | 76.069 | Yes | No | No | -1.16 | No | Yes | No | No | No | No | No | No | 0.519 | No | No | No |
| 120 | 3-Methoxy-5-Phenethylphenol | -4.689 | 1.382 | 94.26 | Yes | Yes | No | 0.081 | No | No | Yes | Yes | Yes | No | No | No | 1.514 | No | Yes | No |
| 121 | Dihydropinosylvin | -3.311 | 1.484 | 93.653 | Yes | No | No | 0.239 | No | No | Yes | No | No | No | No | No | 1.735 | No | No | No |
| 122 | Batatasin Iv | -3.421 | 1.315 | 93.65 | Yes | No | No | 0.11 | No | No | Yes | No | No | No | No | No | 1.631 | No | No | No |
| 123 | 5,7-Dihydroxy-6-Methyl-2-Phenylchromen-4-One | -3.753 | 1.052 | 92.822 | Yes | No | No | 0.013 | No | No | Yes | Yes | No | No | No | Yes | 1.195 | No | No | No |
| 124 | Bauhinoxepin H | -3.147 | 0.991 | 98.154 | Yes | No | No | -0.225 | No | No | Yes | No | No | No | No | Yes | 0.588 | No | No | No |
| 125 | Bauhinoxepin F | -4.055 | 1.016 | 92.788 | Yes | No | No | -0.265 | No | Yes | Yes | Yes | No | No | No | Yes | 0.983 | No | No | No |
| 126 | Bauhiniastatin 1 | -3.189 | 1.006 | 98.269 | Yes | No | No | -0.168 | No | No | Yes | No | No | No | No | Yes | 0.6 | No | No | No |
| 127 | Bauhinoxepin E | -3.87 | 1.179 | 92.727 | Yes | No | No | -0.238 | No | Yes | Yes | Yes | Yes | No | No | No | 0.58 | No | No | No |
| 128 | Bauhiniastatin 2 | -3.874 | 1.122 | 92.887 | Yes | No | No | -0.139 | No | Yes | Yes | Yes | Yes | No | No | No | 0.491 | No | No | No |
| 129 | Pacharin | -3.642 | 1.221 | 92.979 | Yes | No | No | 0.244 | No | Yes | Yes | Yes | No | No | No | No | 0.516 | No | Yes | No |
| 130 | Strobopinin | -3.26 | 1.051 | 92.736 | Yes | No | No | -0.44 | No | Yes | Yes | Yes | Yes | No | No | No | -0.558 | No | No | No |
| 131 | Bauhinoxepin J | -2.372 | 1.316 | 98.934 | No | No | No | 0.23 | No | Yes | Yes | Yes | No | No | No | No | 0.387 | No | No | No |
| 132 | 2-(3,5-Dimethoxyphenethyl)Phenol | -3.766 | 1.717 | 93.278 | Yes | No | No | 0.285 | No | Yes | Yes | Yes | No | No | No | Yes | 0.582 | No | No | No |
| 133 | Bauhinol E | -3.547 | 1.223 | 93.5 | Yes | No | No | -0.043 | No | Yes | Yes | Yes | No | No | No | No | 0.63 | No | No | No |
| 134 | Bauhibenzofurin A | -3.681 | 1.303 | 94.741 | No | No | No | -0.087 | No | Yes | Yes | Yes | No | No | No | No | 0.37 | No | No | No |
| 135 | Bauhinoxepin C | -3.573 | 1.2 | 93.893 | Yes | No | No | 0.24 | No | Yes | Yes | Yes | No | No | No | No | 0.502 | No | Yes | No |
| 136 | Bauhinoxepin D | -3.573 | 1.2 | 93.893 | Yes | No | No | 0.24 | No | Yes | Yes | Yes | No | No | No | No | 0.502 | No | Yes | No |
| 137 | Bauhinoxepin G | -3.795 | 1.223 | 93.21 | Yes | No | No | 0.252 | No | No | Yes | Yes | Yes | No | No | No | 0.66 | No | No | No |
| 138 | 5,7-Dihydroxychromone | -2.479 | 0.637 | 92.688 | No | No | No | 0.084 | No | Yes | No | No | No | No | No | No | 0.563 | No | No | No |
| 139 | Bauhiniastatin 4 | -3.95 | 1.248 | 91.696 | Yes | No | No | 0.369 | No | Yes | Yes | Yes | Yes | No | No | No | 0.615 | No | No | No |
| 140 | Daidzein | -3.793 | 0.903 | 94.839 | Yes | No | No | -0.064 | No | No | Yes | Yes | Yes | No | No | No | 0.187 | No | No | No |
| 141 | Santin | -3.326 | 0.368 | 78.996 | Yes | No | Yes | -0.809 | No | No | Yes | Yes | Yes | No | No | No | 0.328 | No | No | No |
| 142 | Epiafzelechin | -3.254 | 1.077 | 91.482 | Yes | No | No | -0.818 | No | No | No | No | No | No | No | No | 0.136 | No | No | No |
| 143 | Egonol | -4.772 | 1.335 | 93.803 | Yes | Yes | Yes | -0.33 | No | Yes | Yes | Yes | Yes | No | No | No | 0.376 | No | Yes | No |
| 144 | Tricin | -3.276 | 0.12 | 89.713 | Yes | No | No | -1.115 | No | No | Yes | Yes | No | No | No | No | 0.351 | No | No | No |
| 145 | 1,8-Dihydroxy-3-Methyl-4A,9A-Dihydroanthracene-9,10-Dione | -3.301 | 1.249 | 95.266 | Yes | No | No | 0.197 | No | No | No | No | No | No | No | No | 0.011 | No | No | No |
| 146 | (S)-Reticuline | -3.856 | 0.919 | 91.276 | Yes | No | Yes | -0.502 | Yes | Yes | Yes | No | No | No | No | No | 0.232 | No | Yes | No |
| 147 | Norboldine | -3.078 | 1.02 | 92.61 | Yes | No | Yes | -0.824 | Yes | Yes | Yes | No | No | Yes | No | Yes | 0.562 | No | Yes | No |
| 148 | Paulownin | -3.503 | 1.375 | 97.121 | No | Yes | No | -0.566 | No | Yes | No | No | No | No | Yes | No | -0.035 | No | No | No |
| 149 | Xanthyletin | -3.419 | 1.192 | 97.891 | No | No | No | 0.283 | No | No | Yes | No | No | No | No | No | -0.01 | No | No | No |
| 150 | Farnesol | -5.393 | 1.495 | 91.531 | No | No | No | 0.66 | No | No | No | No | No | No | No | No | 0.096 | No | No | No |
| 151 | Cyclovalone | -4.417 | 0.709 | 90.027 | No | Yes | No | -0.251 | No | Yes | No | Yes | Yes | No | Yes | No | 0.131 | No | Yes | No |
| 152 | Bisdemethoxycurcumin | -3.38 | 0.957 | 91.159 | Yes | No | No | -0.089 | No | Yes | Yes | Yes | Yes | No | Yes | No | -0.08 | No | Yes | No |
| 153 | Flavanone | -3.89 | 1.219 | 97.7 | No | No | No | -1.472 | No | Yes | Yes | Yes | No | No | No | No | 0.678 | No | No | No |
| 154 | Zerumbone | -4.027 | 1.432 | 95.781 | No | No | No | 0.522 | No | No | No | No | No | No | No | No | 0.534 | No | No | No |
| 155 | Tetramethoxycurcumin | -6.248 | 1.13 | 95.048 | No | Yes | Yes | -0.783 | No | Yes | Yes | Yes | Yes | No | Yes | No | 0.671 | No | No | No |
| 156 | Labdadienedial | -6.024 | 1.627 | 98.235 | No | No | No | 0.058 | No | Yes | Yes | No | No | No | No | No | -0.869 | No | No | Yes |
| 157 | Dehydrodeguelin | -4.869 | 1.192 | 99.522 | No | Yes | Yes | -0.286 | No | Yes | Yes | Yes | Yes | No | Yes | No | -0.073 | No | No | No |
| 158 | 1,7-Bis(4-Hydroxyphenyl)-1,4,6-Heptatrien-3-One | -3.716 | 1.308 | 90.803 | Yes | No | No | -0.175 | No | Yes | Yes | Yes | Yes | No | No | No | 0.177 | No | Yes | No |
| 159 | O-Demethyldemethoxycurcumin | -3.212 | -0.28 | 76.46 | Yes | Yes | No | -0.947 | No | Yes | Yes | Yes | Yes | No | No | No | -0.237 | No | No | No |
| 160 | Mono-O-Demethylcurcumin | -3.314 | -0.463 | 75.291 | Yes | Yes | Yes | -1.158 | No | Yes | No | Yes | No | No | Yes | No | -0.166 | No | Yes | No |
| 161 | Letestuianin C | -3.394 | 0.947 | 91.536 | Yes | Yes | No | -0.144 | No | Yes | Yes | Yes | Yes | No | No | No | -0.024 | No | Yes | No |
| 162 | Curcumenol | -3.421 | 1.525 | 95.613 | No | No | No | 0.545 | No | No | No | No | No | No | No | No | 0.321 | No | No | No |
| 163 | Procurcumadiol | -2.486 | 1.267 | 95.526 | No | No | No | -0.053 | No | No | No | No | No | No | No | No | 0.597 | No | No | No |
| 164 | Skimmianine | -3.021 | 1.398 | 97.355 | No | No | No | -0.036 | No | Yes | Yes | Yes | No | No | No | Yes | -3.042 | No | No | No |
| 165 | Hydroxy-Alpha-Sanshool | -3.972 | 1.429 | 92.828 | No | No | No | -0.263 | No | No | No | No | No | No | No | No | -0.01 | No | No | No |
| 166 | Hydroxy-Beta-Sanshool | -3.972 | 1.429 | 92.828 | No | No | No | -0.263 | No | No | No | No | No | No | No | No | -0.01 | No | No | No |
| 167 | Piperitol | -4.005 | 1.097 | 94.653 | No | Yes | No | -0.984 | No | Yes | No | Yes | Yes | No | Yes | No | -0.07 | No | No | No |
| 168 | Pluviatilol | -4.485 | 1.003 | 97.056 | Yes | Yes | Yes | -0.06 | No | Yes | Yes | Yes | No | No | Yes | No | 0.051 | No | Yes | No |
| 169 | Laurifoline | -3.847 | 1.568 | 95.833 | Yes | Yes | Yes | -0.416 | No | Yes | Yes | No | No | No | No | No | 0.131 | No | Yes | No |
| 170 | Wighteone | -3.743 | 0.844 | 92.119 | Yes | No | Yes | -0.964 | No | Yes | Yes | Yes | Yes | No | No | No | 0.377 | No | No | No |
| 171 | Alpinumisoflavone | -3.603 | 1.163 | 94.521 | Yes | No | Yes | 0.074 | No | Yes | Yes | Yes | Yes | No | Yes | No | 0.014 | No | Yes | Yes |
| 172 | Erysovine | -2.771 | 1.892 | 94.3 | No | No | No | 0.357 | No | Yes | No | No | No | No | No | No | -0.946 | No | No | Yes |
| 173 | (-)-Epicatechin | -3.117 | -0.283 | 68.829 | Yes | No | No | -1.054 | No | No | No | No | No | No | No | No | 0.483 | No | No | No |
| 174 | Norathyriol | -3.163 | -0.295 | 78.127 | Yes | No | No | -1.091 | No | No | Yes | No | No | No | No | Yes | 0.496 | No | No | No |
| 175 | 1,7-Dihydroxyxanthone | -3.54 | 1.032 | 95.285 | Yes | No | No | -0.039 | No | No | Yes | No | No | No | No | Yes | 0.233 | No | No | No |
| 176 | Garcinone D | -3.54 | 1.032 | 95.285 | Yes | No | No | -0.039 | No | No | Yes | No | No | No | No | Yes | 0.233 | No | No | No |
| 177 | 1,3,7-Trihydroxy-2-Prenylxanthone | -3.676 | 1.068 | 93.864 | Yes | No | Yes | -0.94 | No | Yes | Yes | Yes | Yes | No | No | Yes | 0.356 | No | Yes | No |
| 178 | Br-Xanthone A | -3.909 | 1.313 | 92.919 | Yes | Yes | Yes | -0.277 | No | Yes | Yes | Yes | Yes | No | No | No | -0.198 | No | Yes | Yes |
| 179 | Maclurin | -3.056 | -0.383 | 55.358 | Yes | No | No | -1.094 | No | No | No | No | No | No | No | No | 0.505 | No | No | No |
| 180 | 2,3,6,8-Tetrahydroxy-1-(3-Methylbut-2-Enyl)Xanthen-9-One | -2.892 | -0.144 | 65.383 | Yes | Yes | Yes | -1.819 | No | Yes | No | No | No | No | No | No | 0.438 | No | Yes | No |
| 181 | Cudraxanthone | -3.928 | 1.115 | 93.703 | Yes | No | Yes | -0.104 | No | Yes | Yes | Yes | Yes | No | No | Yes | -0.068 | No | Yes | No |
| 182 | 2,4,6,3',5'-Pentahydroxybenzophenone | -3.107 | -0.434 | 65.713 | Yes | No | No | -1.18 | No | No | No | No | No | No | No | No | 0.419 | No | No | No |
| 183 | 11Alpha-Mangostanin | -3.964 | 0.535 | 88.838 | Yes | Yes | Yes | -1.156 | No | Yes | No | Yes | Yes | No | Yes | Yes | 0.208 | No | Yes | No |
| 184 | 3,4,5,3'-Tetrahydroxybenzophenone | -3.193 | -0.391 | 69.848 | Yes | No | No | -1.095 | No | No | No | No | No | No | No | No | 0.37 | No | No | No |
| 185 | Aminoglutethimide | -2.465 | 0.143 | 95.665 | No | No | No | 0.236 | No | No | No | No | No | No | No | Yes | 0.131 | No | No | No |
| 186 | Euxanthone | -3.54 | 1.032 | 95.285 | Yes | No | No | -0.039 | No | No | Yes | No | No | No | No | Yes | 0.233 | No | No | No |
| 187 | Xanthene-9-Thione | -5.235 | 1.507 | 95.389 | Yes | No | No | 0.446 | No | Yes | Yes | Yes | Yes | Yes | No | Yes | 0.206 | No | No | No |
| 188 | 11-Hydroxy-1-Isomangostin | -3.862 | 0.404 | 87.546 | Yes | Yes | Yes | -1.167 | No | Yes | No | No | Yes | No | No | Yes | 0.417 | No | No | No |
| 189 | 11-hydroxy-3-O-methyl-1-isomangostin | -4.939 | 0.663 | 96.923 | Yes | Yes | Yes | -0.403 | No | Yes | No | Yes | Yes | No | Yes | No | 0.107 | No | Yes | No |
| 190 | Mangostanol | -3.751 | 0.534 | 87.526 | Yes | Yes | Yes | -1.243 | No | Yes | No | Yes | Yes | No | No | Yes | 0.308 | No | Yes | No |
| 191 | Diphyllin | -4.392 | 1.351 | 96.596 |  | Yes | Yes | -0.693 | No | Yes | Yes | Yes | Yes | No | Yes | Yes | -0.336 | No | Yes | No |
| 192 | Berkeleyone A | -5.03 | 0.872 | 76.913 | Yes | Yes | Yes | -0.268 | No | Yes | No | No | No | No | No | No | -0.53 | No | No | No |
| 193 | Berkazaphilone C | -3.073 | 0.874 | 73.105 | Yes | No | Yes | -0.892 | No | No | No | No | No | No | No | No | -0.19 | No | No | Yes |
| 194 | Berkeleyone C | -3.929 | 0.685 | 51.84 | Yes | No | No | -0.282 | No | Yes | No | No | No | No | No | No | 0.034 | No | No | No |
| 195 | Berkeleyamide A | -2.946 | 1.039 | 93.948 | Yes | No | No | -0.332 | No | Yes | No | No | No | No | No | No | 0.153 | No | No | No |
| 196 | Berkazaphilone B | -2.972 | 0.689 | 84.159 | Yes | No | No | -0.921 | No | No | No | No | No | No | No | No | -0.064 | No | No | Yes |
| 197 | Preaustinoid A | -5.177 | 0.903 | 82.197 | No | Yes | No | -0.329 | No | Yes | No | No | No | No | No | No | -0.416 | No | No | No |
| 198 | Mitorubrinol | -3.396 | 0.641 | 57.033 | Yes | No | No | -0.859 | No | No | No | No | No | No | No | Yes | 0.079 | No | No | No |
| 199 | T-Zeatin | -2.604 | 1.263 | 79.581 | Yes | No | No | -1.376 | No | No | No | No | No | No | No | Yes | 0.983 | No | No | Yes |
| 200 | Propyl Gallate | -2.113 | 0.052 | 92.439 | Yes | No | No | -1.132 | No | No | No | No | No | No | No | Yes | -0.294 | No | No | No |
| 201 | Arctigenin | -4.261 | 1.164 | 94.176 | No | Yes | Yes | -0.582 | No | Yes | Yes | Yes | Yes | No | Yes | No | -0.1 | No | No | No |
| 202 | Damnacanthal | -3.373 | 1.183 | 98.335 | No | No | No | -0.297 | No | Yes | No | No | No | No | No | Yes | 0.285 | No | No | No |
| 203 | Rubrofusarin | -3.223 | 1.026 | 93.479 | Yes | No | No | -0.304 | No | Yes | Yes | Yes | Yes | No | No | Yes | 0.191 | No | No | No |
| 204 | Alpinetin | -3.521 | 1.298 | 93.897 | No | No | No | 0.069 | No | Yes | Yes | Yes | Yes | No | No | Yes | 0.136 | No | No | No |
| 205 | Dihydromikanolide | -3.283 | 0.781 | 100 | No | No | No | -0.493 | No | Yes | No | No | No | No | No | No | 0.413 | No | No | No |
| 206 | Mikanin | -3.318 | 0.589 | 99.538 | Yes | No | Yes | -0.749 | No | No | Yes | Yes | No | No | Yes | No | 0.262 | No | No | No |
| 207 | Xerantolide | -3.137 | 1.287 | 99.945 | No | No | No | 0.085 | No | No | Yes | No | No | No | No | No | 0.297 | No | No | No |
| 208 | Scandenolide | -3.467 | 0.82 | 100 | No | No | No | -0.724 | No | Yes | No | No | No | No | No | No | -0.022 | No | No | No |
| 209 | Resveratrol | -3.178 | 1.17 | 90.935 | Yes | No | No | -0.048 | No | Yes | Yes | Yes | No | No | No | Yes | 0.0331 | No | No | No |
| 210 | Morin | -2.978 | -0.294 | 75.408 | Yes | No | No | -1.18 | No | No | Yes | No | No | No | No | No | 0.537 | No | No | No |
| 211 | Taxifolin | -3.042 | 0.924 | 64.709 | Yes | No | No | -0.725 | No | No | No | No | No | No | No | No | 0.345 | No | No | No |
| 212 | Butein | -3.177 | -0.021 | 72.567 | Yes | No | No | -0.895 | No | No | Yes | No | No | No | No | No | 0.117 | No | No | No |
| 213 | Cis-Resveratrol | -3.178 | 1.17 | 90.935 | Yes | No | No | -0.048 | No | Yes | Yes | Yes | No | No | No | Yes | 0.331 | No | No | No |
| 214 | Oxyresveratrol | -3.293 | 1.005 | 87.586 | Yes | No | No | -0.899 | No | Yes | Yes | Yes | No | No | No | No | 0.249 | No | No | No |
| 215 | Moracin M | -3.688 | 0.902 | 90.508 | Yes | No | No | -0.884 | No | Yes | Yes | Yes | Yes | No | Yes | No | 0.311 | No | No | No |
| 216 | Moracin C | -3.885 | 0.806 | 91.206 | Yes | No | No | -0.95 | No | Yes | Yes | Yes | Yes | Yes | Yes | Yes | 0.372 | No | Yes | No |
| 217 | Moracin P | -3.525 | 1.009 | 94.448 | Yes | No | Yes | -0.992 | No | Yes | Yes | Yes | Yes | No | No | No | 0.369 | No | No | No |
| 218 | Glabrone | -3.635 | 1.093 | 96.194 | Yes | No | Yes | 0.031 | No | Yes | Yes | Yes | Yes | No | Yes | No | 0.016 | No | Yes | Yes |
| 219 | Morusinol | -3.536 | -0.04 | 86.917 | Yes | Yes | Yes | -1.384 | No | Yes | No | Yes | Yes | No | No | No | 0.476 | No | Yes | No |
| 220 | Moscatin | -3.817 | 1.194 | 94.766 | Yes | No | Yes | 0.117 | No | Yes | Yes | Yes | Yes | No | No | Yes | -0.052 | No | Yes | No |
| 221 | Norartocarpetin | -3.191 | 0.03 | 87.843 | Yes | No | No | -1.066 | No | No | Yes | No | Yes | No | No | No | 0.432 | No | No | No |
| 222 | Epitaxifolin | -3.042 | 0.924 | 64.079 | Yes | No | No | -0.725 | No | No | No | No | No | No | No | No | -0.345 | No | No | No |
| 223 | Atalantoflavone | -3.614 | 1.076 | 93.839 | Yes | No | Yes | -0.283 | No | Yes | Yes | Yes | Yes | No | No | No | -0.033 | No | No | No |
| 224 | Moracin D | -4.406 | 0.82 | 92.362 | Yes | No | Yes | 0.266 | No | Yes | Yes | Yes | Yes | No | No | Yes | 0.087 | No | Yes | No |
| 225 | (2R,3S)-2-(3,4-Dihydroxyphenyl)-3,5,7-Trihydroxy-2,3-Dihydrochromen-4-One | -3.042 | 0.924 | 64.709 | Yes | No | No | -0.725 | No | No | No | No | No | No | No | No | 0.345 | No | No | No |
| 226 | Moracin N | -3.918 | 0.953 | 91.579 | Yes | No | Yes | -0.946 | No | Yes | Yes | Yes | Yes | No | No | No | 0.51 | No | Yes | No |
| 227 | Morachalcone A | -3.324 | -0.192 | 74.973 | Yes | Yes | Yes | -1.055 | No | Yes | Yes | Yes | Yes | No | Yes | No | 0.214 | No | Yes | No |
| 228 | 5-(6-Hydroxy-1-Benzofuran-2-Yl)-2,2-Dimethylchromen-7-Ol | -4.362 | 0.638 | 91.395 | Yes | No | Yes | 0.126 | No | Yes | Yes | Yes | Yes | Yes | Yes | No | -0.079 | No | No | No |
| 229 | Moracin O | -3.526 | 1 | 93.959 | Yes | No | Yes | -1.021 | No | Yes | Yes | Yes | Yes | No | No | No | 0.379 | No | No | No |
| 230 | 5-(4-Methoxy-8-Methyl-7,10-Dihydrofuro[2,3-G][1]Benzoxepin-2-Yl)Benzene-1,3-Diol | -4.242 | 0.754 | 92.961 | Yes | No | Yes | -0.313 | No | Yes | Yes | Yes | Yes | No | Yes | No | 0.046 | No | Yes | No |
| 231 | Moracin X | -4.64 | 0.701 | 91.177 | Yes | No | Yes | 0.408 | Yes | Yes | Yes | Yes | Yes | No | Yes | No | 0.371 | No | Yes | No |
| 232 | 5-(8-Methyl-7,10-Dihydrofuro[2,3-G][1]Benzoxepin-2-Yl)Benzene-1,3-Diol | -4.455 | 0.799 | 92.837 | Yes | No | Yes | 0.11 | No | Yes | Yes | Yes | Yes | No | No | No | 0.173 | No | Yes | No |
| 233 | Steppogenin | -3.253 | -0.094 | 74.687 | Yes | No | No | -0.827 | No | No | No | No | No | No | No | No | 0.014 | No | No | No |
| 234 | Dihydromorin | -3.079 | -0.216 | 65.43 | Yes | No | No | -0.839 | No | No | No | No | No | No | No | No | 0.206 | No | No | No |
| 235 | (2R)-2-(3,4-Dihydroxyphenyl)-3,5,7-Trihydroxy-2,3-Dihydrochromen-4-One | -3.042 | 0.924 | 64.709 | Yes | No | No | -0.725 | No | No | No | No | No | No | No | No | 0.345 | No | No | No |
| 236 | Licarine A | -4.438 | 1.389 | 93.83 | Yes | Yes | No | 0.061 | No | Yes | Yes | Yes | Yes | No | Yes | Yes | 0.174 | No | Yes | No |
| 237 | Acuminatin | -5.518 | 1.045 | 96.407 | Yes | Yes | Yes | -0.168 | No | Yes | Yes | Yes | Yes | No | No | No | 0.202 | No | No | No |
| 238 | Demethoxymatteucinol | -3.325 | 1.124 | 91.366 | Yes | No | No | -0.143 | No | Yes | Yes | Yes | Yes | No | No | Yes | -0.449 | No | No | No |
| 239 | Dihydroguaiareticacid | -3.713 | 1.231 | 91.337 | Yes | Yes | Yes | 0.008 | No | Yes | Yes | Yes | Yes | No | Yes | No | 0.029 | No | Yes | No |
| 240 | Oleiferin C | -4.345 | 1.345 | 93.444 | Yes | Yes | Yes | -0.42 | No | Yes | Yes | Yes | Yes | No | Yes | No | 0.198 | No | Yes | No |
| 241 | Dihydrokaempferol | -2.967 | 0.996 | 59.072 | Yes | No | No | -0.588 | No | No | No | No | No | No | No | No | 0.353 | No | No | No |
| 242 | 3-Deoxysappanchalcone | -3.206 | 1.177 | 91.809 | Yes | No | No | -0.183 | No | Yes | Yes | Yes | Yes | No | Yes | Yes | -0.364 | No | No | No |
| 243 | 7-Trimethylkaempferol | -3.935 | 1.161 | 95.355 | Yes | No | Yes | -0.711 | No | Yes | Yes | Yes | No | No | Yes | No | 0.249 | No | No | No |
| 244 | Rhamnocitrin | -3.433 | -0.005 | 87.511 | Yes | No | No | -1.101 | No | Yes | Yes | No | No | No | No | No | 0.305 | No | No | No |
| 245 | 2-(4-Hydroxyphenyl)-3,4-Dihydro-2H-Chromene-3,5,7-Triol | -3.254 | 1.077 | 91.482 | Yes | No | No | -0.818 | No | No | No | No | No | No | No | No | 0.136 | No | No | No |
| 246 | Phyllaemblic acid methy ester | -3.99 | 1.089 | 71.896 | Yes | No | No | -0.524 | No | No | No | No | No | No | No | No | 0.438 | No | No | No |
| 247 | Digiferruginol | -2.748 | -0.027 | 97.552 | Yes | No | No | -0.115 | No | No | Yes | No | No | No | No | Yes | 0.704 | No | No | No |
| 248 | Sakuranetin | -3.14 | 1.364 | 92.601 | Yes | No | No | -0.216 | No | No | Yes | Yes | No | No | No | No | -0.031 | No | No | No |
| 249 | Evofolin B | -3.396 | 0.33 | 82.72 | Yes | No | No | -0.956 | No | Yes | No | No | No | No | No | Yes | 0.353 | No | No | No |
| 250 | Decussatin | -3.692 | 1.102 | 96.002 | Yes | No | Yes | -0.582 | No | Yes | Yes | Yes | No | No | No | Yes | 0.227 | No | No | No |
| 251 | 1,2,6,8-Tetrahydroxyxanthen-9-One | -3.214 | -0.246 | 75.148 | Yes | No | No | -1.006 | No | No | Yes | No | No | No | No | Yes | 0.344 | No | No | No |
| 252 | Deacetylgedunin | -4.756 | 0.744 | 97.515 | No | No | No | -0.351 | No | Yes | No | No | No | No | Yes | No | -0.671 | No | No | Yes |
| 253 | Methylangolensate | -5.504 | 0.972 | 100 | No | Yes | No | -0.681 | No | Yes | No | No | No | No | Yes | No | -0.6 | No | No | No |
| 254 | Methyl (1S,4As,5As,6S,10As)-1-Methyl-2'-Oxospiro[1,4A,5,5A,7,8,10,10A-Octahydropyrano[3,4-F]Indolizine-6,3'-1H-Indole]-4-Carboxylate | -3.521 | 1.119 | 96.483 | Yes | No | No | 0.035 | No | Yes | No | No | No | No | No | No | -1.088 | No | No | Yes |
| 255 | Pinocembrin | -3.538 | 1.152 | 92.417 | Yes | No | No | 0.42 | No | No | Yes | Yes | Yes | No | No | No | 0.269 | No | No | No |
| 256 | Sandaracopimaradienediol | -4.751 | 1.734 | 95.006 | No | Yes | No | 0.299 | No | Yes | No | No | No | No | No | No | 0.343 | No | No | No |
