# Supplementary material for: Computational and immunoinformatics approaches for designing phytocompound-based drugs and a multi-epitope vaccine targeting FemA, a cell wall protein of Staphylococcus aureus
Source: PLoS One. 2026 Apr 7;21(4):e0346271. doi: 10.1371/journal.pone.0346271 (PMC13056209; doi:10.1371/journal.pone.0346271)
Supplement: S3 Table — (DOCX) [file pone.0346271.s003.docx]

**S3 Table. The binding affinity of the phytocompounds.**

| **SL No** | **FemA docking with** | **Read-1** | **Read-2** | **Read-3** | **Average** |
| --- | --- | --- | --- | --- | --- |
| 1 | Nimbinin | -8.6 | -8.6 | -8.6 | -8.6 |
| 2 | Epoxyazadiradione | -8.6 | -8.6 | -8.6 | -8.6 |
| 3 | Dehydrodeguelin | -8.2 | -8.7 | -8.5 | -8.5 |
| 4 | 17-Hydroxyazadiradione | -8.5 | -8.3 | -8.5 | -8.4 |
| 5 | 17-Epi-17-Hydroxyazadiradione | -8.4 | -8.3 | -8.4 | -8.4 |
| 6 | Nimbolide | -8.2 | -8.2 | -8.3 | -8.2 |
| 7 | Paulownin | -8.3 | -8.2 | -8.1 | -8.2 |
| 8 | Preaustinoid A | -8.1 | -8.1 | -8.1 | -8.1 |
| 9 | Moracin P | -8 | -8.2 | -8.1 | -8.1 |
| 10 | Moracin N | -8.1 | -8 | -8.1 | -8.1 |
| 11 | Ethinyl Estradiol | -8 | -8 | -8.1 | -8.0 |
| 12 | 7-Hydroxy-2-(4-Hydroxyphenyl)Chromen-4-One | -7.9 | -7.9 | -8 | -7.9 |
| 13 | Pluviatilol | -7.7 | -8 | -8 | -7.9 |
| 14 | Androstenedione | -7.8 | -7.7 | -7.7 | -7.7 |
| 15 | Beta-Nimolactone | -7.7 | -7.7 | -7.7 | -7.7 |
| 16 | Farrerol | -7.7 | -7.7 | -7.7 | -7.7 |
| 17 | 7Beta,18-Dihydroxydehydroabietanol | -7.7 | -7.7 | -7.7 | -7.7 |
| 18 | Moracin X | -7.6 | -7.9 | -7.6 | -7.7 |
| 19 | Atalantoflavone | -7.5 | -8 | -7.5 | -7.7 |
| 20 | Strobopinin | -7.3 | -7.8 | -7.8 | -7.6 |
| 21 | Desfuranoazadiradione | -7.5 | -7.5 | -7.7 | -7.6 |
| 22 | Nordentatin | -7.6 | -7.6 | -7.5 | -7.6 |
| 23 | Piperitol | -7.7 | -7.5 | -7.5 | -7.6 |
| 24 | Madagascin | -7.6 | -7.4 | -7.6 | -7.5 |
| 25 | Cheilanthifoline | -7.4 | -7.4 | -7.5 | -7.4 |
| 26 | Egonol | -7.5 | -7.4 | -7.4 | -7.4 |
| 27 | Bauhinoxepin C | -7.4 | -7.4 | -7.4 | -7.4 |
| 28 | Pinocembrin | -7.4 | -7.4 | -7.4 | -7.4 |
| 29 | Apigenin | -7.4 | -7.3 | -7.4 | -7.4 |
| 30 | 7-Oxodehydroabietinol | -6.9 | -7.7 | -7.4 | -7.3 |
| 31 | Alpha-Nimolactone | -7.4 | -7.1 | -7.4 | -7.3 |
| 32 | Naringenin | -7.4 | -7.1 | -7.4 | -7.3 |
| 33 | Moracin M | -7.3 | -7.3 | -7.3 | -7.3 |
| 34 | Sakuranetin | -7.4 | -7.1 | -7.4 | -7.3 |
| 35 | Sesalin | -6.8 | -7.5 | -7.5 | -7.3 |
| 36 | Chrysin | -7.2 | -7.2 | -7.2 | -7.2 |
| 37 | Bauhinoxepin D | -7.2 | -7.2 | -7.2 | -7.2 |
| 38 | Bauhiniastatin 4 | -7.2 | -7.2 | -7.2 | -7.2 |
| 39 | Rhamnocitrin | -7.2 | -7.2 | -7.2 | -7.2 |
| 40 | Sandaracopimaradienediol | -7 | -7.4 | -7.1 | -7.2 |
| 41 | Methylangolensate | -7.1 | -7.1 | -7.1 | -7.1 |
| 42 | Xanthyletin | -6.8 | -7.6 | -6.8 | -7.1 |
| 43 | Dihydromikanolide | -7 | -7.1 | -7.1 | -7.1 |
| 44 | Acuminatin | -6.6 | -7.9 | -6.6 | -7.0 |
| 45 | Nimbinone | -7 | -7 | -7 | -7.0 |
| 46 | Xanthoxyletin | -7.1 | -6.8 | -7.1 | -7.0 |
| 47 | Bauhinoxepin J | -6.8 | -7.1 | -7.1 | -7.0 |
| 48 | Santamarin | -7 | -6.9 | -7 | -7.0 |
| 49 | Daidzein | -7.1 | -6.9 | -6.9 | -7.0 |
| 50 | Epiafzelechin | -7.1 | -6.9 | -6.9 | -7.0 |
| 51 | 2-(4-Hydroxyphenyl)-3,4-Dihydro-2H-Chromene-3,5,7-Triol | -6.9 | -7.1 | -6.9 | -7.0 |
| 52 | Ayanin | -6.9 | -6.9 | -6.9 | -6.9 |
| 53 | Isoflavone | -6.7 | -7.2 | -6.7 | -6.9 |
| 54 | Xerantolide | -6.8 | -7 | -6.8 | -6.9 |
| 55 | Lariciresinol | -6.9 | -6.7 | -6.8 | -6.8 |
| 56 | Galangin | -6.9 | -6.7 | -6.8 | -6.8 |
| 57 | Genistein | -6.8 | -6.8 | -6.8 | -6.8 |
| 58 | Curcumenol | -6.8 | -6.8 | -6.8 | -6.8 |
| 59 | 1,3-Dibenzylurea | -6.5 | -6.9 | -6.9 | -6.8 |
| 60 | Bauhiniastatin 2 | -6.7 | -6.8 | -6.8 | -6.8 |
| 61 | Mikanin | -6.6 | -7 | -6.6 | -6.7 |
| 62 | 7-Trimethylkaempferol | -6.7 | -6.8 | -6.7 | -6.7 |
| 63 | 12-Hydroxydehydroabietic Acid | -6.7 | -6.7 | -6.7 | -6.7 |
| 64 | Lasiodiplodin | -6.7 | -6.7 | -6.7 | -6.7 |
| 65 | Laurifoline | -6.8 | -6.6 | -6.6 | -6.7 |
| 66 | 1,8-Dihydroxy-3-Methyl-4A,9A-Dihydroanthracene-9,10-Dione | -6.6 | -6.6 | -6.6 | -6.6 |
| 67 | 1,7-Bis(4-Hydroxyphenyl)-1,4,6-Heptatrien-3-One | -6.7 | -6.4 | -6.6 | -6.6 |
| 68 | Bauhibenzofurin A | -6.5 | -6.6 | -6.6 | -6.6 |
| 69 | Aloechrysone | -6.6 | -6.4 | -6.6 | -6.5 |
| 70 | Pinosylvin | -6.7 | -6.4 | -6.5 | -6.5 |
| 71 | Bauhinoxepin E | -6 | -6.8 | -6.8 | -6.5 |
| 72 | Bauhinoxepin G | -6.3 | -6.6 | -6.5 | -6.5 |
| 73 | Arctigenin | -6.4 | -6.6 | -6.4 | -6.5 |
| 74 | Aframodial | -5.9 | -6.6 | -6.7 | -6.4 |
| 75 | Dentatin | -6.7 | -6.2 | -6.2 | -6.4 |
| 76 | Dihydropinosylvin | -6.3 | -6.3 | -6.3 | -6.3 |
| 77 | Procurcumadiol | -6.3 | -6.2 | -6.4 | -6.3 |
| 78 | Oxyresveratrol | -6.3 | -6.3 | -6.3 | -6.3 |
| 79 | Pacharin | -6.2 | -6.3 | -6.2 | -6.2 |
| 80 | Cnidilin | -5.9 | -6.8 | -5.9 | -6.2 |
| 81 | Vasicinolone | -6.2 | -6.3 | -6 | -6.2 |
| 82 | Matairesinol | -6.3 | -5.9 | -6.3 | -6.2 |
| 83 | 3-Methoxy-5-Phenethylphenol | -6.8 | -5.8 | -5.8 | -6.1 |
| 84 | Reticuline | -4.9 | -6.7 | -6.6 | -6.1 |
| 85 | Isoeugenitol | -6.2 | -5.8 | -6.2 | -6.1 |
| 86 | Eudesmane-4Alpha,11-Diol | -6 | -5.9 | -6.1 | -6.0 |
| 87 | Bauhinol E | -5.6 | -6.2 | -6.2 | -6.0 |
| 88 | Berkeleyamide A | -6 | -6 | -6 | -6.0 |
| 89 | Sinugibberodiol | -6.2 | -5.8 | -5.9 | -6.0 |
| 90 | Zerumbone | -6 | -5.9 | -6 | -6.0 |
| 91 | Oleiferin C | -5.4 | -6.9 | -5.4 | -5.9 |
| 92 | Vasicinone | -5.4 | -6.1 | -6.1 | -5.9 |
| 93 | Bisdemethoxycurcumin | -5.3 | -6.6 | -5.3 | -5.7 |
| 94 | Batatasin Iv | -5.2 | -5.9 | -5.9 | -5.7 |
| 95 | Vasicinol | -5.3 | -5.7 | -5.5 | -5.5 |
| 96 | Tetramethoxycurcumin | -4.9 | -6.3 | -4.9 | -5.4 |
| 97 | Letestuianin C | -5.1 | -5.7 | -5.2 | -5.3 |
| 98 | Cis-linalool-oxide | -4.9 | -5.1 | -5 | -5.0 |
| 99 | 6-Dehydrogingerdione | -4.6 | -5.6 | -4.6 | -4.9 |
| 100 | Eugenyl Acetate | -4.8 | -4.7 | -4.8 | -4.8 |
| 101 | Diethyl Phthalate | -4.9 | -4.2 | -4.9 | -4.7 |
| 102 | 6-Paradol | -5.3 | -4.2 | -4.3 | -4.6 |
| 103 | Pyridoxine Hydrochloride | -4.8 | -4.4 | -4.5 | -4.6 |
| 104 | Dihydroguaiareticacid | -4.5 | -4.7 | -4.5 | -4.6 |
| 105 | Sinapinate | -4.3 | -4.9 | -4.3 | -4.5 |
| 106 | Hydroxy-Beta-Sanshool | -4.3 | -4.7 | -4.5 | -4.5 |
| 107 | Farnesol | -4.3 | -4.8 | -4.3 | -4.5 |
| 108 | Hydroxy-Alpha-Sanshool | -4 | -4.6 | -4.6 | -4.4 |
| 109 | Citronellyl isobutyrate | -4.3 | -4.5 | -4.3 | -4.4 |
| 110 | Ajoene | -3.4 | -3.8 | -3.4 | -3.5 |
| 111 | N-heptane | -3.5 | -3.4 | -3.5 | -3.5 |
