# Supplementary material for: Computational and immunoinformatics approaches for designing phytocompound-based drugs and a multi-epitope vaccine targeting FemA, a cell wall protein of Staphylococcus aureus
Source: PLoS One. 2026 Apr 7;21(4):e0346271. doi: 10.1371/journal.pone.0346271 (PMC13056209; doi:10.1371/journal.pone.0346271)
Supplement: S4 Table — (DOCX) [file pone.0346271.s004.docx]

**S4 Table. Predicted CTL epitopes and their physicochemical properties.**

| **Serial No.** | **Residue Number** | **Epitope** | **Supertypes** | **Combined Score** | **Antigenicity** | **Allergenicity** | **Toxicity** | **Homology** | **Immunogenicity** |
| --- | --- | --- | --- | --- | --- | --- | --- | --- | --- |
| 1 | 314 | GYNAEIIEY | A1 | 0.6871 | 0.8839 | No | Non-toxin | Non-homologue | 0.43087 |
| 2 | 43 | NQELVHFFF | A1 | 0.6114 | 1.1277 | No | Non-toxin | Non-homologue | 0.25919 |
| 3 | 54 | LHIDPYLPY | A1 | 0.5858 | 1.9037 | No | Non-toxin | Non-homologue | 0.0354 |
| 4 | 187 | SYAVQWEMI | A24 | 1.74 | 1.2519 | No | Non-toxin | Non-homologue | 0.13185 |
| 5 | 253 | NQELVHFFF | A24 | 1.1481 | 1.1277 | No | Non-toxin | Non-homologue | 0.25919 |
| 6 | 387 | GYETHLVGI | A24 | 0.8586 | 0.8216 | No | Non-toxin | Non-homologue | 0.14726 |
| 7 | 120 | LHIDPYLPY | A26 | 0.5286 | 1.9037 | No | Non-toxin | Non-homologue | 0.0354 |
| 8 | 59 | FFINPFEVV | B8 | 0.6915 | 1.3766 | No | Non-toxin | Non-homologue | 0.24471 |
| 9 | 290 | TQTVGHYEL | B39 | 1.7019 | 0.6697 | No | Non-toxin | Non-homologue | 0.17297 |
| 10 | 324 | LQEEHGNEL | B39 | 1.3922 | 1.1759 | No | Non-Toxin | Non-homologue | 0.24969 |
| 11 | 110 | LHIDPYLPY | B39 | 0.63 | 1.9037 | No | Non-Toxin | Non-homologue | 0.0354 |
| 12 | 67 | NQELVHFFF | B39 | 0.5939 | 1.1277 | No | Non-Toxin | Non-homologue | 0.25919 |
| 13 | 45 | EEHGNELPI | B44 | 1.415 | 1.2659 | No | Non-Toxin | Non-homologue | 0.11671 |
| 14 | 299 | TQTVGHYEL | B44 | 0.9471 | 0.6697 | No | Non-Toxin | Non-homologue | 0.17297 |
| 15 | 154 | LQEEHGNEL | B44 | 0.8676 | 1.1759 | No | Non-Toxin | Non-homologue | 0.24969 |
| 16 | 22 | TEDAEDAGV | B44 | 0.7266 | 1.1469 | No | Non-Toxin | Non-homologue | 0.21777 |
| 17 | 97 | NQELVHFFF | B44 | 0.6142 | 1.1277 | No | Non-Toxin | Non-homologue | 0.25919 |
| 18 | 65 | LAEGYETHL | B44 | 0.6065 | 0.8408 | No | Non-Toxin | Non-homologue | 0.20891 |
| 19 | 111 | LHIDPYLPY | B62 | 1.1347 | 1.9037 | No | Non-Toxin | Non-homologue | 1.6955333 |
| 20 | 214 | LQEEHGNEL | B62 | 1.0663 | 1.1759 | No | Non-Toxin | Non-homologue | 1.6670375 |
| 21 | 59 | TQTVGHYEL | B62 | 0.9073 | 0.6697 | No | Non-Toxin | Non-homologue | 1.6607211 |
| 22 | 76 | KLAEGYETH | B62 | 0.8047 | 1.0336 | No | Non-Toxin | Non-homologue | 1.6496116 |
| 23 | 45 | GYNAEIIEY | B62 | 0.7878 | 0.8839 | No | Non-Toxin | Non-homologue | 1.6395218 |
| 24 | 44 | NQELVHFFF | B62 | 0.7557 | 1.1277 | No | Non-Toxin | Non-homologue | 1.6221428 |
