# Supplementary material for: Computational and immunoinformatics approaches for designing phytocompound-based drugs and a multi-epitope vaccine targeting FemA, a cell wall protein of Staphylococcus aureus
Source: PLoS One. 2026 Apr 7;21(4):e0346271. doi: 10.1371/journal.pone.0346271 (PMC13056209; doi:10.1371/journal.pone.0346271)
Supplement: S5 Table — (DOCX) [file pone.0346271.s005.docx]

**S5 Table. Predicted HTL epitopes and their physicochemical properties.**

| **SL No.** | **Epitope** | **Antigenicity** | **Allergenicity** | **Toxicity** | **Homology** | **IL-4** | **IL-10** | **Immunogenicity** |
| --- | --- | --- | --- | --- | --- | --- | --- | --- |
| 1 | YVKKHRCLYLHIDPY | 0.9087 | No | Non-toxin | Non-homologue | Yes | Yes | -0.31626406 |
| 2 | YLPYQYLNHDGEITG | 0.8107 | No | Non-toxin | Non-homologue | Yes | Yes | -0.37958275 |
| 3 | YIKELNEERDILNKD | 0.7883 | No | Non-toxin | Non-homologue | Yes | Yes | -0.19674044 |
| 4 | YENQELVHFFFNELS | 0.2057 | No | Non-toxin | Non-homologue | Yes | Yes | -0.031588895 |
| 5 | VPVMKVFKYFYSNRG | 0.0099 | No | Non-toxin | Non-homologue | Yes | Yes | -0.39562828 |
| 6 | VMKVFKYFYSNRGPV | 0.7339 | No | Non-toxin | Non-homologue | Yes | Yes | -0.27067635 |
| 7 | VLQIRYHSVLDLKDK | 0.8764 | No | Non-toxin | Non-homologue | Yes | Yes | -0.16685681 |
| 8 | VKVRFLSEEELPIFR | 0.1658 | No | Non-toxin  9 | Non-homologue | Yes | Yes | -0.13461371 |
| 9 | ENQELVHFFFNELSK | 0.0367 | No | Non-toxin | Non-homologue | Yes | Yes | -0.10890283 |
| 10 | VKKHRCLYLHIDPYL | 1.1932 | No | Non-toxin | Non-homologue | Yes | Yes | -0.17147726 |
| 11 | VHFFFNELSKYVKKH | 0.0367 | No | Non-toxin | Non-homologue | Yes | Yes | -0.32538669 |
| 12 | VFKYFYSNRGPVIDY | -0.0228 | No | Non-toxin | Non-homologue | Yes | Yes | -0.014433536 |
| 13 | TAVPVMKVFKYFYSN | 0.1709 | No | Non-toxin | Non-homologue | Yes | Yes | -0.38153156 |
| 14 | TADDIIKNMDGLRKR | 0.6924 | No | Non-toxin | Non-homologue | Yes | Yes | -0.2886351 |
| 15 | QIRYHSVLDLKDKTA | 0.7968 | No | Non-toxin | Non-homologue | Yes | Yes | -0.16184102 |
| 16 | QELVHFFFNELSKYV | -0.2108 | No | Non-toxin | Non-homologue | Yes | Yes | -0.16607259 |
| 17 | PYLPYQYLNHDGEIT | 0.7384 | No | Non-toxin | Non-homologue | Yes | Yes | -0.30621212 |
| 18 | PVMKVFKYFYSNRGP | -0.0850 | No | Non-toxin | Non-homologue | Yes | Yes | -0.7149011 |
| 19 | NQELVHFFFNELSKY | -0.0165 | No | Non-toxin | Non-homologue | Yes | Yes | -0.18548596 |
| 20 | NMDGLRKRNTKKVKK | 1.3436 | No | Non-toxin | Non-homologue | Yes | Yes | -0.14947302 |
| 21 | NGVKVRFLSEEELPI | 0.6356 | No | Non-toxin | Non-homologue | Yes | Yes | -0.21882815 |
| 22 | NELSKYVKKHRCLYL | 0.1087 | No | Non-toxin | Non-homologue | Yes | Yes | 0.000540723 |
| 23 | LQIRYHSVLDLKDKT | 0.9864 | No | Non-toxin | Non-homologue | Yes | Yes | -0.32209444 |
| 24 | LVHFFFNELSKYVKK | 0.1054 | No | Non-toxin | Non-homologue | Yes | Yes | -0.075508142 |
| 25 | MKVFKYFYSNRGPVI | -0.1911 | No | Non-toxin | Non-homologue | Yes | Yes | -0.15951526 |
| 26 | NELPISAGFFFINPF | 0.7032 | No | Non-toxin | Non-homologue | Yes | Yes | 0.075788993 |
| 27 | LPISAGFFFINPFEV | 0.9504 | No | Non-toxin | Non-homologue | Yes | Yes | 0.60314136 |
| 28 | NGVKVRFLSEEELPI | 0.6356 | No | Non-toxin | Non-homologue | Yes | Yes | -0.21882815 |
| 29 | LHIDPYLPYQYLNHD | 1.2840 | No | Non-toxin | Non-homologue | Yes | Yes | -0.39422145 |
| 30 | KNMDGLRKRNTKKVK | 1.4580 | No | Non-toxin | Non-homologue | Yes | Yes | -0.44823914 |
| 31 | KKHRCLYLHIDPYLP | 1.2678 | No | Non-toxin | Non-homologue | Yes | Yes | -0.40150146 |
| 32 | KHRCLYLHIDPYLPY | 1.2739 | No | Non-toxin | Non-homologue | Yes | Yes | -0.42953239 |
| 33 | ISAGFFFINPFEVVY | 0.8806 | No | Non-toxin | Non-homologue | Yes | Yes | 0.89980367 |
| 34 | IKNMDGLRKRNTKKV | 1.0898 | No | Non-toxin | Non-homologue | Yes | Yes | -0.31736715 |
| 35 | IDPYLPYQYLNHDGE | 1.0507 | No | Non-toxin | Non-homologue | Yes | Yes | -0.46541477 |
| 36 | HRCLYLHIDPYLPYQ | 1.1692 | No | Non-toxin | Non-homologue | Yes | Yes | -0.4258334 |
| 37 | HFFFNELSKYVKKHR | -0.0990 | No | Non-toxin | Non-homologue | Yes | Yes | -0.004220358 |
| 38 | GVKVRFLSEEELPIF | 0.5200 | No | Non-toxin | Non-homologue | Yes | Yes | -0.28003202 |
| 39 | EYIKELNEERDILNK | -0.3053 | No | Non-toxin | Non-homologue | Yes | Yes | -0.15550948 |
| 40 | ENQELVHFFFNELSK | 0.0367 | No | Non-toxin | Non-homologue | Yes | Yes | -0.10890283 |
| 41 | ELPISAGFFFINPFE | 0.6442 | No | Non-toxin | Non-homologue | Yes | Yes | 0.36339264 |
| 42 | DYENQELVHFFFNEL | 0.3469 | No | Non-toxin | Non-homologue | Yes | Yes | -0.052361639 |
| 43 | DPVLQIRYHSVLDLK | 1.1573 | No | Non-toxin | Non-homologue | Yes | Yes | 0.051654201 |
| 44 | DDIIKNMDGLRKRNT | 0.5121 | No | Non-toxin | Non-homologue | Yes | Yes | -0.28204777 |
| 45 | CLLTAVPVMKVFKYF | 0.2703 | No | Non-toxin | Non-homologue | Yes | Yes | -0.21473403 |
| 46 | AVPVMKVFKYFYSNR | 0.0368 | No | Non-toxin | Non-homologue | Yes | Yes | -0.2710382 |
| 47 | ACLLTAVPVMKVFKY | 0.3198 | No | Non-toxin | Non-homologue | Yes | Yes | -0.15657478 |
| 48 | AACLLTAVPVMKVFK | 0.2127 |  |  |  | Yes | Yes | -0.36057063 |
