# Supplementary material for: Computational and immunoinformatics approaches for designing phytocompound-based drugs and a multi-epitope vaccine targeting FemA, a cell wall protein of Staphylococcus aureus
Source: PLoS One. 2026 Apr 7;21(4):e0346271. doi: 10.1371/journal.pone.0346271 (PMC13056209; doi:10.1371/journal.pone.0346271)
Supplement: S6 Table — (DOCX) [file pone.0346271.s006.docx]

**S6 Table. Predicted LBL epitopes and their physicochemical properties.**

| **Serial No** | **Epitope** | **Probability Score** | **Antigenicity** | **Allergenicity** | **Toxicity** | **Homology** | **Immunogenicity** |
| --- | --- | --- | --- | --- | --- | --- | --- |
| 1 | NMDGLRKRNTKK | 0.8681 | 1.4724 | No | Non-Toxin | Non-Homologue | 0.52846193 |
| 2 | KRNTKKVKKNGV | 0.8568 | 1.1765 | No | Non-Toxin | Non-Homologue | 0.074946237 |
| 3 | KNMDGLRKRNTK | 0.8546 | 1.444 | No | Non-Toxin | Non-Homologue | 0.50804562 |
| 4 | GLRKRNTKKVKK | 0.8472 | 1.3701 | No | Non-Toxin | Non-Homologue | 0.1237901 |
| 5 | RNTKKVKKNGVK | 0.8367 | 1.3578 | No | Non-Toxin | Non-Homologue | 0.5952853 |
| 6 | DGLRKRNTKKVK | 0.8186 | 1.587 | No | Non-Toxin | Non-Homologue | 0.65322953 |
| 7 | DPVLQIRYHSVL | 0.8244 | 1.3226 | No | Non-Toxin | Non-Homologue | 0.71553423 |
